# Computational Identification of Key Regulators in Two Different Colorectal Cancer Cell Lines

**DOI:** 10.3389/fgene.2016.00042

**Published:** 2016-04-05

**Authors:** Darius Wlochowitz, Martin Haubrock, Jetcy Arackal, Annalen Bleckmann, Alexander Wolff, Tim Beißbarth, Edgar Wingender, Mehmet Gültas

**Affiliations:** ^1^Institute of Bioinformatics, University Medical Center GöttingenGöttingen, Germany; ^2^Department of Hematology/Medical Oncology, University Medical Center GöttingenGöttingen, Germany; ^3^Department of Medical Statistics, University Medical Center GöttingenGöttingen, Germany

**Keywords:** colorectal cancer, promoter analysis, pathway analysis, master regulator analysis, Wnt pathway

## Abstract

Transcription factors (TFs) are gene regulatory proteins that are essential for an effective regulation of the transcriptional machinery. Today, it is known that their expression plays an important role in several types of cancer. Computational identification of key players in specific cancer cell lines is still an open challenge in cancer research. In this study, we present a systematic approach which combines colorectal cancer (CRC) cell lines, namely 1638N-T1 and CMT-93, and well-established computational methods in order to compare these cell lines on the level of transcriptional regulation as well as on a pathway level, i.e., the cancer cell-intrinsic pathway repertoire. For this purpose, we firstly applied the Trinity platform to detect signature genes, and then applied analyses of the geneXplain platform to these for detection of upstream transcriptional regulators and their regulatory networks. We created a CRC-specific position weight matrix (PWM) library based on the TRANSFAC database (release 2014.1) to minimize the rate of false predictions in the promoter analyses. Using our proposed workflow, we specifically focused on revealing the similarities and differences in transcriptional regulation between the two CRC cell lines, and report a number of well-known, cancer-associated TFs with significantly enriched binding sites in the promoter regions of the signature genes. We show that, although the signature genes of both cell lines show no overlap, they may still be regulated by common TFs in CRC. Based on our findings, we suggest that canonical Wnt signaling is activated in 1638N-T1, but inhibited in CMT-93 through cross-talks of Wnt signaling with the VDR signaling pathway and/or LXR-related pathways. Furthermore, our findings provide indication of several master regulators being present such as MLK3 and Mapk1 (ERK2) which might be important in cell proliferation, migration, and invasion of 1638N-T1 and CMT-93, respectively. Taken together, we provide new insights into the invasive potential of these cell lines, which can be used for development of effective cancer therapy.

## 1. Introduction

Cancer undergoes genetic and epigenetic changes through which it acquires cellular and molecular characteristics during invasive tumor growth. These changes allow the tumor cells to evade the immune response, activate the microenvironment, invade surrounding tissues and metastasize to distant sites. The microenvironment plays an important role in this context as it may trigger anti-tumor as well as pro-tumor signals (Gao et al., 2014). Malignant tumor cells stimulate the production and secretion of growth factors, cytokines and enzymes, thereby recruiting the stroma and vasculature, which altogether results in the conversion of a normal tumor-inhibiting into a tumor-promoting microenvironment (Gao et al., 2014). In that respect, tumor aggressiveness can be linked to processes such as cell proliferation, growth, invasion, metastasis, survival as well as inflammation which are regulated by multiple signal transduction pathways. It has been suggested to summarize known signal transduction reactions into about 17 signal transduction pathways (Nebert, 2002). They are usually activated by growth factor signals from the cell surface, and further transmit the signal via transmembrane receptors to their target intracellular effectors. In tumor cells, these pathways are often dysregulated and harbor alterations in key components that can function as driver mutations, i.e., either as activation mutations (Ras, PI3K, Akt) or loss of tumor-suppressor gene function (Pten). Several cancer drivers are important integral parts of these pathways, such as receptor tyrosine kinases, and can be located upstream in signal transduction cascades. Since protein kinases propagate the signals along the cascade, they are considered attractive drug targets for therapeutic intervention using specific protein kinase inhibitors (Zwick et al., 2001; Torkamani et al., 2009; Takeuchi and Ito, 2011; Casaletto and McClatchey, 2012). To this end, many anticancer agents have been used in the context of cancer therapy to account for the number of different pathways (Casaletto and McClatchey, 2012).

The signaling pathways are interconnected and form an elaborate network of pathways that receives signals from a variety of growth factors to tightly regulate processes such as transcription, cell growth, motility, differentiation, apoptosis, and cytoskeletal organization. In addition, the outcome triggered by the integrated signaling may differ between different cell types. Therefore, knowledge on the cell type-specific pathways including their architecture and complexity provides important information on the tumor cell behavior during inhibitor therapy, i.e., the inhibitor may not achieve the desired outcome due to the utilization of alternative bypass pathways in certain tumor cells.

Signal transduction pathways converge on sets of genes with similar key functions which are regulated by upstream transcription factors (TFs). TFs occupy short and specific DNA-sequences denoted as transcription factor binding sites (TFBSs). TFs and their corresponding TFBSs recruit and regulate the transcription machinery, thereby governing selective temporal and spatial activities of their target genes. Moreover, many TFs play important roles as oncogenes and they are usually activated downstream in the signaling cascades. Consequently, their deregulated expression, aberrant activation as well as mutations contribute to tumorigenesis. For example, the TP53 gene which encodes an important transcription factor with tumor suppressor function in cancer, is known to be the most commonly mutated gene in human cancer (Kandoth et al., 2013). Unsurprisingly, TFs are central to cancer and became highly desirable points of interference in cancer gene therapy (Libermann and Zerbini, 2006). In this regard, three major transcription factor families have been considered highly desirable drug targets: (i) the NF-κB and AP-1 families of TFs; (ii) the STAT family members; (iii) the steroid receptors (Libermann and Zerbini, 2006). Although other additional TF families have been implicated in cancer to this day, there is still no comprehensive library on TFs and their specific roles in cancer and, particularly, in different cancer cell types. However, given the tumor heterogeneity and cancer cell plasticity, it can be expected that many more TFs will be associated with potentially important roles in oncogenic pathways of different cancers.

The third most common cancer in the world is colorectal cancer (CRC) which originates in the epithelial cells of the gastrointestinal track and shows a high tendency to metastasize into the liver. CRC is often caused by mutations in two well-studied signal transduction pathways, namely the Wnt and the EGFR pathways (Normanno et al., 2006; Polakis, 2012). Mouse models have been extensively used in cancer studies to directly monitor the metastatic progression in CRC. The ability to study primary tumors as well as distant metastatic sites and to manipulate the spatial and temporal expression levels of certain single genes have proven the animal model technology to be a powerful tool in cancer progression research. Such studies have often made use of APC-deficient mouse models since mutations in the adenomatous polyposis coli (APC), an important component of the Wnt signaling pathway, occur in the majority of human CRC cells (Karim and Huso, 2013). It is estimated that the canonical Wnt/β-catenin signaling pathway is abnormally activated in over 90% of CRCs (Cancer Genome Atlas Network, 2012). Briefly, the canonical Wnt pathway revolves around the intracellular levels of the transcriptional coactivator β-catenin which forms a complex with TCF/LEF, thereby controlling the expression of Wnt signaling targets, such as c-Myc and cyclin D. β-Catenin is degraded by a destruction complex that includes the tumor suppressor APC and other proteins (Stamos and Weis, 2013). Loss of APC leads to a constant activation of WNT signaling, which promotes proliferation of tumor cells.

The bottleneck in cancer research has always been a lack of effective tools to comprehensively study the complex networks of signaling pathways (Kang, 2005; Gupta and Massagué, 2006). Therefore, cancer research has largely taken advantage of the integration of animal models and bioinformatic approaches. Microarrays and nowadays RNA-sequencing techniques (RNA-Seq) are used to infer reliable gene regulatory networks based on the level of all expressed transcripts (transcriptome) (Schena et al., 1995; Mortazavi et al., 2008). The result of a transcriptome profiling experiment can be summarized in a set of expressed genes or transcription units that are meaningful for a certain experimental condition, disease state or developmental process. These technologies have led to paradigm-shifting advances in cancer research. For example, gene expression profiles in combination with supervised clustering approaches were used in breast cancer studies which successfully discriminated between cancer patients with good prognosis from those with poor prognosis, thereby leading to the identification of prognostic cancer genes (van 't Veer et al., 2002; Weigelt et al., 2005). However, solely using genomic profiling of tumor samples only identifies individual genes of a set of signature genes, but does not provide a functional context for these genes, which is important for a mechanistic understanding of cancer-associated processes. Pathway analyses have therefore emerged as powerful tools by benefiting from the statistical power of entire gene sets using the overrepresentation in biologically defined pathways rather than interpreting meaningful functions based on the expression of individual genes.

Despite the presence of a variety of different approaches and rich literature on cancer research as mentioned above, to date, there is still need for comprehensive analyses to detect key regulators in different colorectal cancer cell lines. In this study, we made use of distinct murine cancer cell lines and system biology approaches to identify signature genes and pathways whose activation may specifically affect invasive tumor growth. In addition, we exhaustively covered a broad range of potentially important signaling pathways and focus our discussion selectively on the study of the roles of various classical and novel signaling pathways in CRC. Moreover, we aimed to highlight the meaning of specific TFs in the context of these pathways on the basis of enriched TFBSs in the promoter regions of the signature genes. We provide a comprehensive library on CRC-specific TFs and exemplarily discuss their roles in both CRC cell lines. Taken together, we identified potential discriminators between the two CRC cell lines as well as points of interference for targeted cancer therapy, thus providing further insights into the complexity of cancer.

## 2. Materials and methods

### 2.1. Colorectal cancer cell lines

The CMT-93 cell line, a mouse colorectal polyploid carcinoma cell line, was purchased from the American Type Culture Collection, Manassas, USA (CCL223) and was cultured in DMEM High Glucose Medium (Gibco, Darmstadt, Germany) supplemented with 10% heat inactivated fetal bovine serum (FCS; Sigma, Munich, Germany). The murine colorectal cancer cell line 1638N-T1, derived from Apc1638N adenomas, was kindly provided by Ron Smits (Smits et al., 1997). Remarkably, this cell line harbors a targeted mutation at codon 1638 of the Apc gene, Apc1638T, leading to a truncated Apc protein (Smits et al., 1999). These were cultured in DMEM High Glucose Medium supplemented with 15% not heat inactivated FCS and Insulin/Transferrin/Selenium Solution (Gibco). In contrast to Smits et al., these cells were not cultured on any fibronectin/collagen/albumin-coated plates and were passaged using 0.05% (w/v) trypsin (Biochrom, Berlin, Germany), as long as they did not show any differences in their morphology, viability and proliferation.

### 2.2. RNA isolation and sequencing

Total RNA was isolated using the TRIzol Reagent (Invitrogen, Karlsruhe, Germany) including a DNase I (Roche, Mannheim, Germany) digestion. RNA integrity and quantity was assessed with the Agilent Bioanalyzer 2100 and the NanoDrop DD-1000 UV vis spectrophotometer version 3.2.1. 2 μg of total RNA were used as start material for library preparation (TruSeq Stranded mRNA Sample Prep Kit from Illumina, Cat NRS-122-2101). Accurate quantitation of cDNA libraries was performed by using the QuantiFluor dsDNA System (Promega). The size range of cDNA libraries was determined applying the DNA 1000 chip on the Bioanalyzer 2100 from Agilent (280 bp). cDNA libraries were amplified and sequenced by using the cBot and HiSeq 2000 from Illumina (SR, 1 × 51 bp, 8–9 Gb > 40 M reads per sample). Sequence images were transformed with Illumina software BaseCaller to bcl-files, which were demultiplexed to FASTQ files with CASAVA (version 1.8.2). Quality check was done via FastQC (version 0.10.1, Babraham Bioinformatics).

### 2.3. Signature gene selection

We started our analyses based on 43433 gene annotations from Ensembl (mouse assembly GRCm38.p4), which were retrieved from RNA-seq samples (Section 2.2; three biological replicates for each cell line; GSE78696). Based on these samples, we obtained signature genes as follows:

Using the Trinity platform (Grabherr et al., 2011), we firstly performed a differentially expressed gene (DEG) analysis based on both cell lines. After that, employing the Trinity platform these DEGs were clustered into three main categories using a *p*-value cutoff for FDR of 0.05 and the default fold change (default: 2 (meaning 2^2^ or 4-fold)): (i) genes which are most significantly upregulated in 1638N-T1 (Supplementary Table S1) and, at the same time, downregulated in CMT-93; (ii) genes which are most significantly upregulated in CMT-93 (Supplementary Table S2) and, at the same time, downregulated in 1638N-T1; (iii) the remaining DEGs which did not fall in the first and second category. In our further analysis, we only considered genes as *signature genes* which fell into the first or second category.

### 2.4. Data processing

For the subsequent analyses we used the geneXplain platform (http://genexplain-platform.com/bioumlweb/), which includes the TRANSFAC and TRANSPATH databases. We used the suggested parameters from this platform if not explicitly stated otherwise.

#### 2.4.1. Enrichment of TFBSs in promoter sequences

We applied a conventional enrichment analysis to the previously identified signature gene sets in order to retrieve specific TFs whose binding sites or sequence motifs are particularly enriched in their genomic regions. For the enrichment analysis, we firstly extracted for each signature gene the corresponding promoter sequence covering the −1000 to 100 bp regions relative to transcription start sites. Second, we used position weight matrices (PWMs) from the TRANSFAC database (Wingender, 2008) to predict potential TFBSs in promoters. However, computational TFBS predictions are generally considered as being flooded with high rates of false predictions. The accurate prediction of TFBSs is still a challenging task. To minimize the rate of false predictions in our analysis, we collected a specific PWM library using literature on CRC (Supplementary Table S3). This library contains 229 colorectal cancer-related non-redundant matrices. In our further analysis, this library was used with the minFP profile (cut-offs minimizing false positive rate) that contains the adjusted thresholds for each PWM to minimize the prediction of false positive TFBSs. Using our library, we then employed the F-MATCH program described in Schmid et al. (2006) to determine the enriched TFBSs in promoters of the signature genes (foreground set) in comparison to a background set which contains genes with very small fold changes (~ 0) in both cell lines under study. For this purpose, F-MATCH program applies an iterative process where the initial thresholds in minFP profile are regularly altered until the best possible thresholds are defined which provide most significantly enriched TFBSs. This enrichment analysis yields important key TFs, which may not be mutated themselves, but their altered activation may potentially lead to a persistent expression of their target signature genes, thereby affecting tumorigenesis.

#### 2.4.2. Overrepresented pathways in colorectal cancer

To gain more insights into the functional properties of the signature genes and their transcriptional regulators in CRC, we investigated the overrepresented pathways. For this purpose, we observed the signal transduction and metabolic pathways from TRANSPATH (Krull et al., 2006) database which contains information about genes/molecules and reactions to build complete networks. In this study, we performed two distinct pathway analyses, of which the first one refers to the overrepresented pathways in the signature genes, and the second one is based on the enriched TFBSs found in the promoters of these signature genes.

#### 2.4.3. Identification of master regulators with TRANSPATH

Master regulators (MRs) are molecules which are at the very top of regulatory hierarchy and, thus, they are not affected by any of their downstream molecules. Their identification provides important knowledge to display functional relationships of genes. In this study, using the TRANSPATH database, we employed a standard workflow with a maximum radius of 10 steps upstream of TFs to identify their potential MRs.

#### 2.4.4. Transformation of PWMs to their corresponding TFs and TF family/subfamily classifications

Multiple PWMs can be assigned to a TF and several TFs belong to a TF family/subfamily. To obtain the correct assignments of the PWMs to their respective TFs and TF family/subfamily, we used the annotations integrated in the geneXplain platform. TF family/subfamily classifications are curated in TFClass (http://tfclass.bioinf.med.uni-goettingen.de/tfclass) which is a classification resource with the aim to catalog TFs based on their DNA-binding characteristics (Wingender et al., 2013). TFClass incorporates a six level classification schema which consists of superclasses, classes, families, subfamilies, genera and factor species of which subfamilies and factor species are optional. At the family level, TFs are primarily grouped on basis of sequence similarities of their DNA-binding domains. The optional subfamily level comprises two more levels which represent genes and gene products, termed genera and species, respectively. TFClass uses a digit-based classification schema which is analogous to the Enzyme Commission numbering system. The schema assigns a four-digit number for the top four classification levels or a six-digit number with respect to the two optional sublevels of the subfamily level.

## 3. Results

Classical discovery of individual markers usually involves the comparison of normal cells vs. cancer cells, which provides candidates for prognosis as well as individualized treatments. In this study, however, we focused on the *in silico* comparative analysis of two distinct cancer cell lines which serve as models to describe pathways. The cancer cell-intrinsic pathway repertoire and their activation status may differ between distinct cancer cell lines of the same cancer type, which in turn may have an impact on invasiveness and organ colonization *in vivo*. Apart from that, it still remains largely unclear as to what extent these processes are promoted or inhibited by the tumor microenvironment. Therefore, it is mandatory to first learn about the cancer cell line-specific pathway repertoire and, further, to test their functional consequences in *in vivo* models. Above all, the cell lines under study represent suitable models to investigate the molecular mechanisms by which mutations cause predisposition to the formation of multiple colorectal tumors. In addition, they can be used to screen for early disease biomarkers, and to develop therapeutic and preventive strategies.

### 3.1. Overview of the analysis workflow

**Figure 1:**
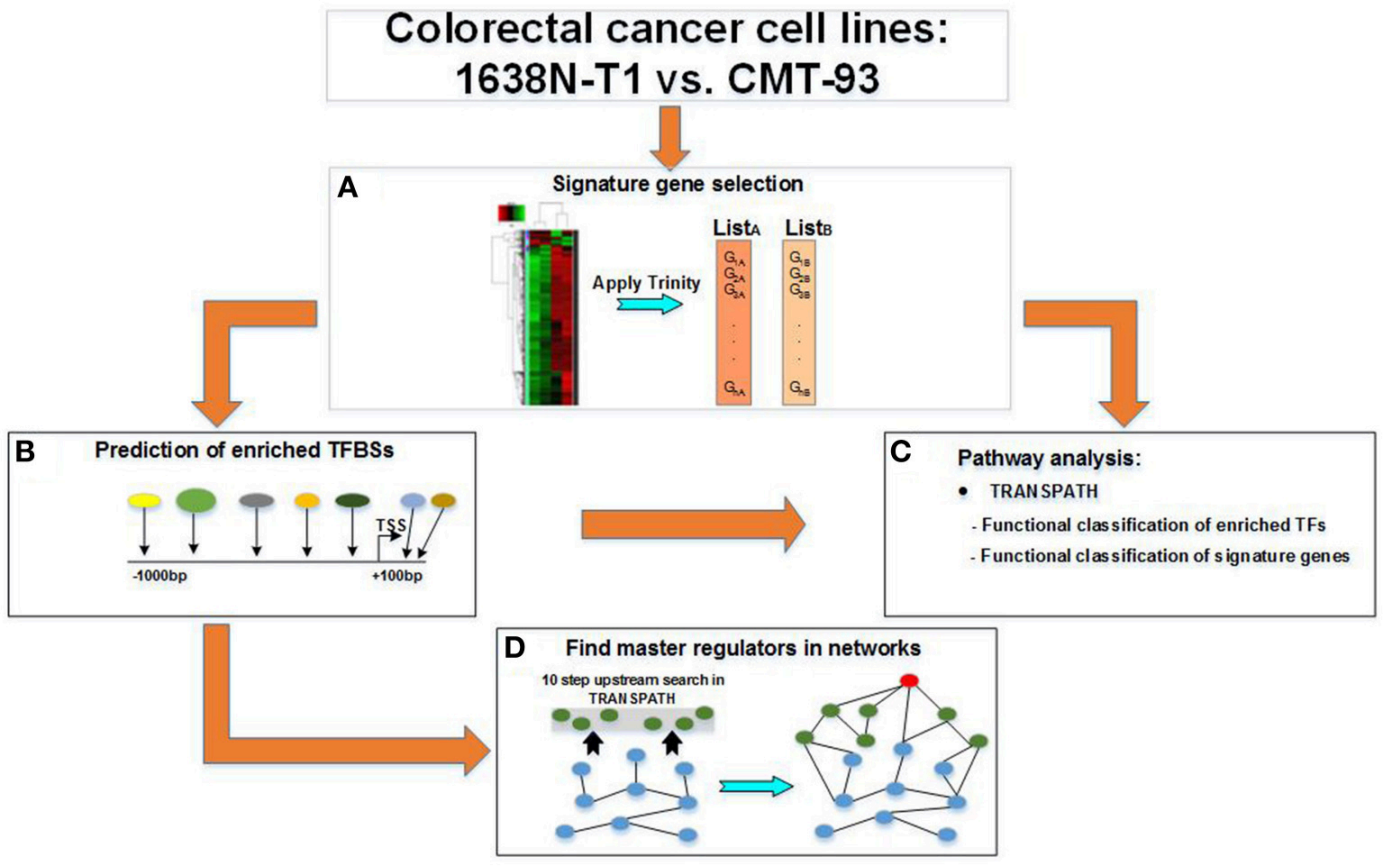
**Workflow for the study of distinct colorectal cancer cell lines**. A multi-step workflow is outlined for the comparison of the 1638N-T1 and CMT-93. **(A)** The analysis begins with the identification of signature genes based on RNA-seq samples using the Trinity platform. This step generates two disjunct lists of signature genes which are further applied to different geneXplain analyses. **(B)** The signature genes are searched for overrepresented TRANSPATH pathways. Enriched transcription factor binding sites (TFBSs) are searched within the −1 kb/+100 bp promoter regions of the signature genes to obtain transcription factors (TFs). **(C)** The TFs are then searched for overrepresented TRANSPATH pathways. **(D)** A master regulatory network is generated by searching for a master regulator (red node) up to 10 steps upstream of the TFs (blue nodes) in TRANSPATH. The master regulator is connected via intermediate molecules (green nodes) with the TFs.

**Figure 2:**
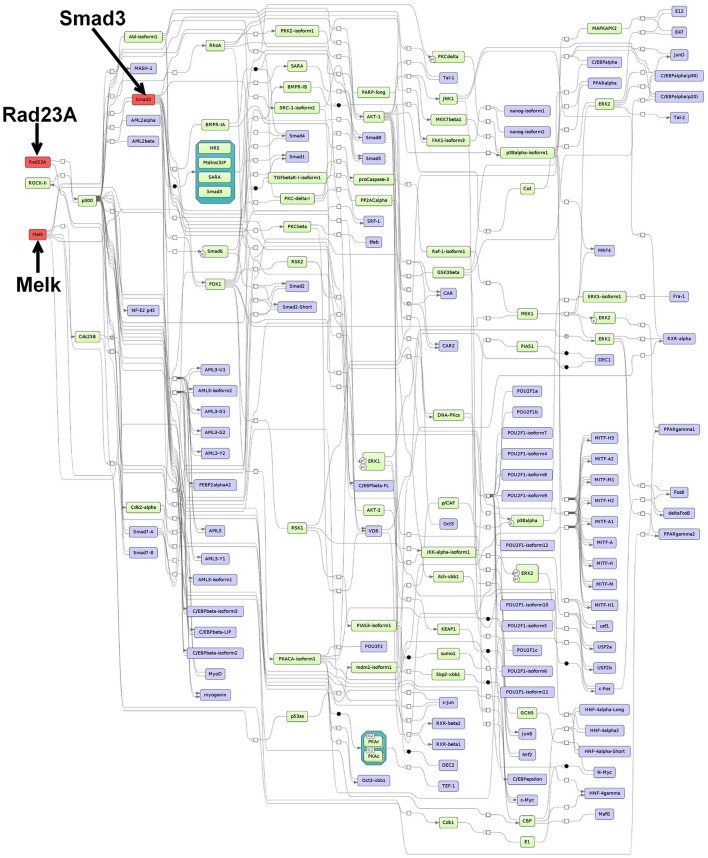
**Master regulatory network based on the intersection-specific TF set**. The color coding red, blue and green represent nodes for master regulators, regulated transcription factors and connecting molecules, respectively.

**Figure 3:**
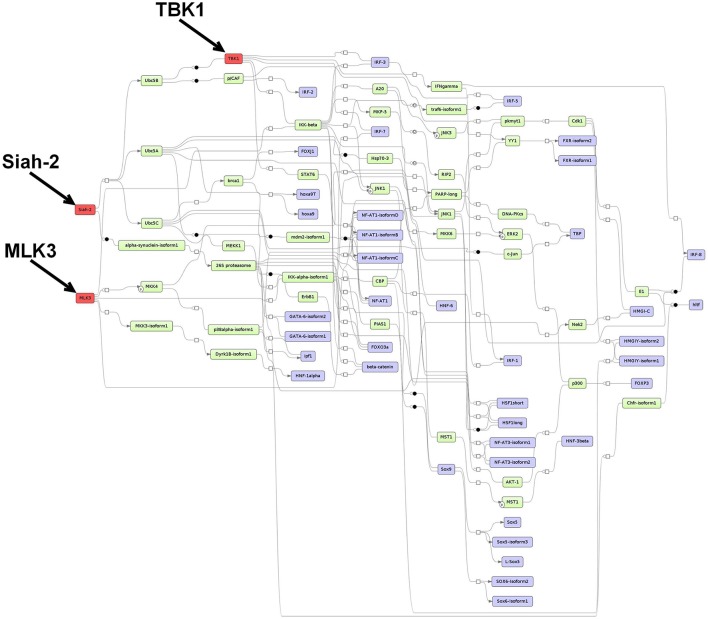
**Master regulatory network based on the 1638NT-1-specific TF set**. The color coding red, blue and green represent nodes for master regulators, regulated transcription factors and connecting molecules, respectively.

**Figure 4:**
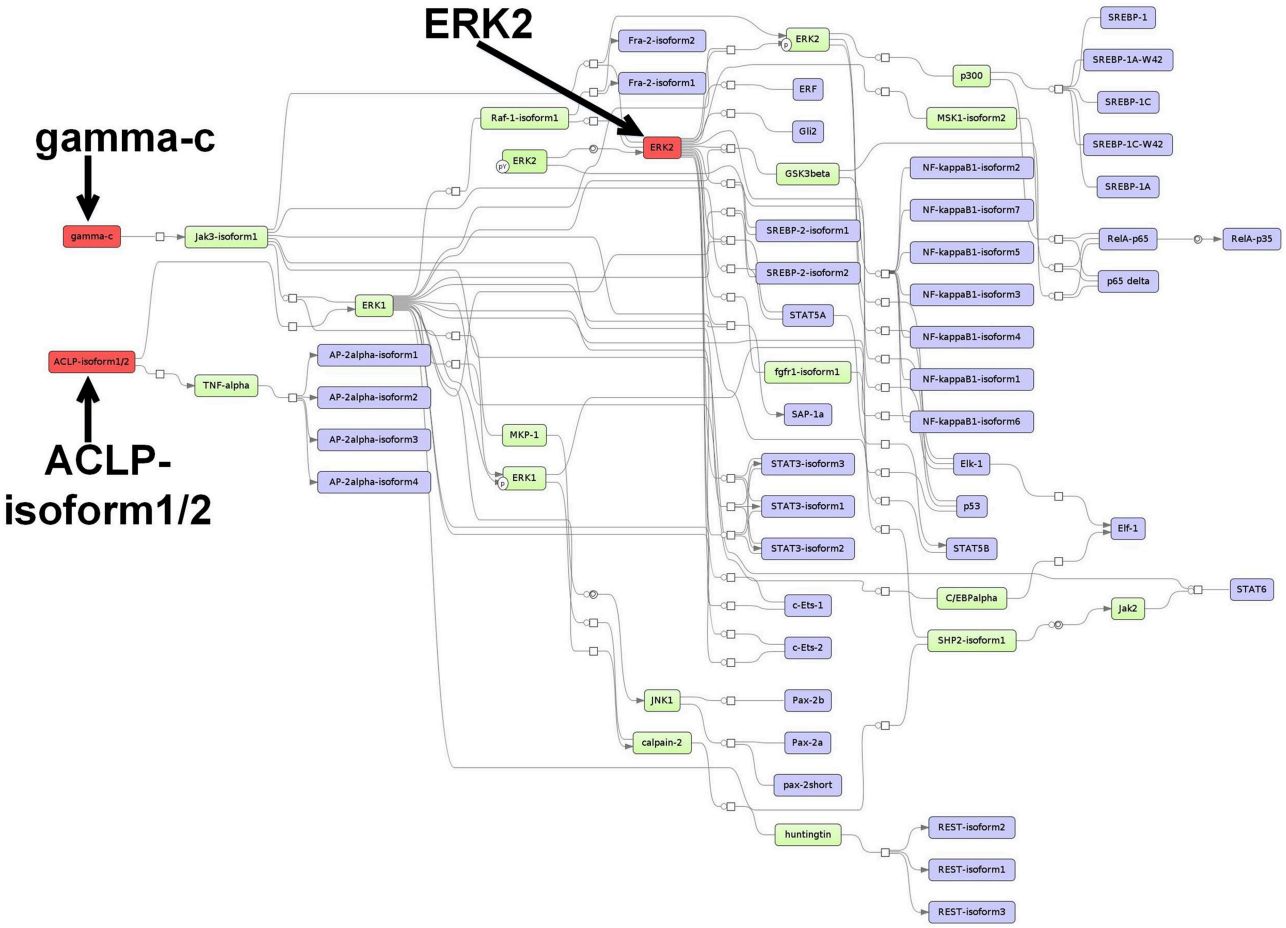
**Master regulatory network based on the CMT-93-specific TF set**. The color coding red, blue and green represent nodes for master regulators, regulated transcription factors and connecting molecules, respectively.

Our workflow involved four major steps of which the first one was performed using the Trinity platform and all following steps using the geneXplain platform as described below (see also Figure 1):

1. Selection of signature genes (Section 3.2)
  - Analysis of differentially expressed transcripts
  - Clustering of the most differentially expressed transcripts
2. Identification of overrepresented TRANSPATH pathways based on signature genes (Section 3.3)
  - Pathway analysis for 1638N-T1 (Section 3.3.1)
  - Pathway analysis for CMT-93 (Section 3.3.2)
3. Identification of transcription factors (TFs) based on signature genes (Section 3.4)
  - Prediction of enriched TFBSs in promoters using a CRC-specific PWM library
  - Mapping of TFBSs to corresponding TFs as well as TF family/subfamily classifications
  - Grouping of TFs as well as TF family/subfamily into three subsets: 1638NT-1- and CMT-93-intersection-specific TF set; 1638NT-1-specific TF set; CMT-93-specific TF set (Sections 3.4.1, 3.4.3, and 3.4.5)
  - Identification of overrepresented TRANSPATH pathways based on the three TF sets (Sections 3.4.2, 3.4.4, and 3.4.6)
4. Identification of upstream master regulators in pathways based on the three TF sets (Section 3.5)
  - Search for master regulators upstream of TRANSPATH-mapped molecules of each TF set (Sections 3.5.1, 3.5.2, and 3.5.3)
  - Merging of master regulator pathways based on the top three master regulators found for each TF set (Figures 2–**4**).

### 3.2. Signature genes

Tumor initiation, promotion and progression is generally driven by genes whose expression is changed in tumor cells. Comparing gene expression profiles and detection of differentially expressed transcripts between different cancer cell lines can reveal molecular characteristics of the tumor cells under study. Using the Trinity platform we identified signature genes based on their altered transcriptional regulation in the context of CRC. In total, 2296 and 2342 Ensembl gene IDs were identified for 1638N-T1 and CMT-93, respectively. Supplementary Tables S1, S2 provide the full sets of signature genes for 1638N-T1 and CMT-93, respectively.

### 3.3. Pathway analyses based on signature gene sets

The molecular characterization of tumor cells and the molecular mechanisms through which tumor cells acquire the capability to grow progressively, survive and metastasize are numerous and depend on genetic and environmental factors. On the other hand, tumor antigens can be recognized by host T cells, thereby triggering an immune response against the colonization of tumor cells. It is partly the activation of immune system suppressive pathways by the tumor cells which can decide whether cancer evades the anti-tumor immune responses and progresses. Moreover, the expression of various cytokines and chemokines controls the balance between anti-tumor immunity and pro-tumor inflammation. Besides cytokines and chemokines, several TFs and enzymes play critical roles in regulatory functions during tumor development. Therefore, analyzing the tumor-specific expression profiles and detection of these molecules, in particular TFs, are crucial steps in studying the molecular characteristics of tumor cells. Moreover, the knowledge about these molecules and their pathways will provide further information on the molecular mechanisms which may be linked to tumor aggressiveness. In this light, we searched for important pathways for 1638N-T1 and CMT-93 based on their signature genes and exemplarily provided references for their roles in cancer. With the previously defined signature gene sets at hand, we obtained overrepresented TRANSPATH pathways using the geneXplain platform.

#### 3.3.1. Pathway analysis for 1638N-T1

In total, 30 TRANSPATH pathways were found to be significantly overrepresented based on the signature genes of 1638N-T1 (Table 1). The top four most overrepresented pathways indicated a role for the signature genes Ugt1a1, Ugt1a2, Ugt1a6a, and Ugt1a7c which encode UDP-glucuronosyltransferases (UGTs). These detoxification enzymes are involved in the metabolism of endogenous and xenobiotic compounds (Cooley et al., 1982; Magnanti et al., 2000). Expression of UGTs has been implicated in human urinary bladder and colon cancer (Giuliani et al., 2005; Wang et al., 2012). Furthermore, the first two of the four pathways related to a mechanism for the detoxification of NNAL (the metabolized isoform of NNK) via UGTs-catalyzed glucuronidation pathways (Wiener et al., 2004). NNK is a tobacco agent widely known for promoting tumorigenesis and metastasis through its pro-inflammatory effects (Takahashi et al., 2010). The remaining two pathways related to glucuronidation pathways which are involved in heme degradation in response to oxidative stress. Heme ingestion leads to hyperproliferation and activation of oncogenes as well as the inhibition of the tumor suppressor p53 in response to increased cytotoxicity in the mouse colon (Ijssennagger et al., 2013). The fifth topmost overrepresented pathway corresponded to the activation of Ras-related protein Rap-1A (Rap1A) via interferon gamma (IFNγ). Rap1A is a tumor suppressor which mediates growth inhibitory responses in cancer (Alsayed et al., 2000). The cytokine IFNγ plays an important role in innate and adaptive immune responses and prevents development of primary and transplanted tumors (Ikeda et al., 2002). Further, the pathway analysis found two putative pro-inflammatory metabolic pathways which involve the molecules eicosanoid hepoxilin A3 (hepA3) and platelet activating factor (PAF), respectively. Both molecules have been suggested to play key roles in inflammation-associated cancer (Mrsny et al., 2004; Tsoupras et al., 2009). Furthermore, the results reported a signaling cascade which leads to the activation of mitogen-activated protein kinase 1 (Mapk1/Erk2) via interleukin-8 (IL-8). Several studies have implicated IL-8 in tumor angiogenesis, growth, and metastasis in colon, gastric and pancreatic carcinoma (Li et al., 2001, 2008; Kuai et al., 2012; Sun et al., 2014a). A recent study showed that IL-8 increases the migration in human CRC cells through the integrin alpha-V/beta-6 and chemokine receptors CXCR1/2 involving the activation of Mapk1 and Ets-1 signaling pathway (Sun et al., 2014a). Another reported pathway relates to the interleukin-3 (IL-3)-induced activation of the JAK2/STAT5 pathway. IL-3 expression via the T cell receptor signaling pathway is known to regulate growth and differentiation of hematopoietic stem cells, neutrophils, eosinophils, megakaryocytes, macrophages, lymphoid and erythroid cells (Reddy et al., 2000). Lastly, the results showed overrepresentation for the activation of Wnt signaling which is aberrantly activated in the majority of CRCs (Cancer Genome Atlas Network, 2012).

**Table 1:** **Overrepresented TRANSPATH pathways for the signature genes of 1638NT-1**.

**Pathway**	**Hit names of signature genes**	***P*-value**
detoxification and bioactivation of tobacco-derived carcinogen NNK	Cbr3, Ugt1a1, Ugt1a2, Ugt1a6a, Ugt1a7c	4.755E-4
NNK → NNAL-O-glucuronide, NNAL-N-glucuronide	Cbr3, Ugt1a1, Ugt1a2, Ugt1a6a, Ugt1a7c	4.755E-4
heme, globin → bilirubin beta-diglucuronide	Ugt1a1, Ugt1a2, Ugt1a6a, Ugt1a7c	0.00326
hemoglobin oxidation	Ugt1a1, Ugt1a2, Ugt1a6a, Ugt1a7c	0.00326
IFNgamma → Rap1	Cybb, Hspa1a, Ifngr1, Ncf4	0.01253
Syk → RhoA	Syk, Vav2	0.01349
Hck → RhoA	Hck, Vav2	0.01349
hepoxilin A3 → Hepoxillin A3-D	Ggt7, Tgm2	0.01349
G-alpha-q → IP3	Cybb, Ncf4, Plcb1	0.01385
BCR → p38	C3,Cybb, Ncf4, Syk	0.01618
BCR —MLK3 → c-Jun	C3, Cybb, Ncf4, Syk	0.01618
catabolism of PAF	Enpp2, Pla2g7, Plcb1, Plcg2	0.01618
alpha IIb beta3 → Rac1	Cybb, Fyb, Ncf4, Prkg1, Syk	0.0211
alpha IIb beta3 pathway	Cybb, Fyb, Ncf4, Prkg1, Syk	0.0211
IL-8 → ERK2	Cxcl1, Cybb, Gnai1, Il8, Ncf4	0.02495
WAVE2 → Arp2/3 complex	Acta1, Actr3b, Cybb, Cyfip2, Ncf4	0.02495
Epo → Syk	Epor, Syk	0.02577
PMCA4 —/ nNOS	Dmd, Snta1	0.02577
Wnt activation of LRP5/6/frizzled/axin complex	Fzd4, Fzd8, Wnt1	0.0268
SDF-1 → G-protein	Cxcr4, Cybb, Gnai1, Ncf4, Pik3r5	0.02923
BCR → cytoskeletal reorganization	C3, Cybb, Ncf4, Syk	0.03089
BCR → c-Jun	C3, Cybb, Ncf4, Syk	0.03089
SLP-65 —/ Raf-1	Cybb, Ncf4, Plcg2	0.03503
dehydroepiandrosterone → estriol 16-glucuronide	Cyp1b1, Cyp4a12a, Cyp4b1, Ugt1a1, Ugt1a2, Ugt1a6a, Ugt1a7c	0.03888
IL-3 → STAT5	Csf2rb, Il3ra	0.04102
beta-glucan —DECTIN1 → IP3, DAG	Plcg2, Syk	0.04102
metabolism of estrogens	Cyp1b1, Cyp4a12a, Cyp4b1, Ugt1a1, Ugt1a2, Ugt1a6a, Ugt1a7c	0.04299
Rac1 —p65PAK → Arp2/3 complex	Acta1, Actr3b, Cybb, Ncf4	0.04396
Src → Rac1	Cybb, Ncf4, Vav2	0.0444
N-cadherin —Eplin → actin	Acta1, Cdh2, Ctnna2	0.0444

#### 3.3.2. Pathway analysis for CMT-93

The pathway analysis resulted in the identification of 28 overrepresented TRANSPATH pathways based on the signature genes of CMT-93 (Table 2). The four topmost overrepresented pathways share 13/14 hit signature genes which are all associated with the assembly of protein complexes called adherens junctions that occur in epithelial and endothelial tissues (Guo et al., 2007). One prominent signature gene amongst these hits was E-cadherin (cadherin-1/CDH1) that belongs to the cadherin superfamily and encodes a calcium-dependent cell adhesion protein. E-cadherin acts as an invasion suppressor and its loss in epithelial carcinomas permits the invasion of adjacent normal tissues. Several studies showed that the level of E-cadherin expression is inversely correlated with tumor malignancy (Vleminckx et al., 1991; Cowin et al., 2005; Junghans et al., 2005). Likewise, protein-protein interactions between E-cadherin and β-catenin result in the formation of a tumor-suppressor system (Müller et al., 1999). The regulation of β-catenin/E-cadherin has been associated with the induction of epithelial-mesenchymal transition (EMT) and metastasis (Morali et al., 2001; Kim et al., 2002; Eger et al., 2004).

**Table 2:** **Overrepresented TRANSPATH pathways for the signature genes of CMT-93**.

**Pathway**	**Hit names of signature genes**	***P*-value**
beta-catenin:E-cadherin complex phosphorylation and dissociation	Axl, Blk, Cdh1, Epha1, Erbb3, Fes, Kit, Lck, Mertk, Ntrk1, Ret, Tek, Txk	0.00147
beta-catenin:E-cadherin complex phosphorylation and dephosphorylation	Axl, Blk, Cdh1, Epha1, Erbb3, Fes, Kit, Lck, Mertk, Ntrk1, Ret, Tek, Txk	0.00147
tyrosine dephosphorylation of plakoglobin	Axl, Blk, Cdh1, Epha1, Erbb3, Fes, Kit, Lck, Mertk, Ntrk1, Ret, Tek, Txk	0.00166
beta-catenin network	Axl, Blk, Cdh1, Epha1, Erbb3, Fes, Kit, Lck, Magi2, Mertk, Ntrk1, Ret, Tek, Txk	0.002
NGF —p75NTR → trkA	Ngf, Ntrk1	0.00464
VDR network	Cyp27a1, Cyp2r1, Hist1h4i, Hist1h4j, Hist2h3c2, Hist2h4, Hist4h4, Vdr	0.00541
NGF → trkA	Ngf, Ntrk1	0.0133
Tie2 dephosphorylation	Ptprb,Tek	0.0133
CO2, H2O → spermine	Arg1, Car14, Car2, Car3, Car6	0.01419
Angiopoietin/Tie signaling	Dok2, Nos3, Ptprb, Sfn, Tek	0.01419
creatine biosynthesis and degradation	Car14, Car2, Car3, Car6, Gatm, Mat1a	0.01625
VDR → RXR-alpha → transcriptional activation	Hist1h4i, Hist1h4j, Hist2h3c2, Hist2h4, Hist4h4, Vdr	0.01891
sphinganine → ceramide-2,3,6,7	Cers1, Cers4, Ugcg	0.01936
urea and aspartate cycles, polyamine and creatine synthesis	Arg1, Car14, Car2, Car3, Car6, Gatm	0.02184
CO2, L-ornithine → L-arginine	Car14, Car2, Car3, Car6	0.02475
p53 → p21Cip1	Hist1h4i, Hist1h4j, Hist2h4, Hist4h4	0.02475
p53 → PUMA	Hist1h4i, Hist1h4j, Hist2h4, Hist4h4	0.02475
7-dehydrocholesterol → calcitriol	Cyp27a1, Cyp2r1	0.02542
formation of vitamin D3 and 1alpha,25-dihydroxycholecalciferol	Cyp27a1, Cyp2r1	0.02542
Nedd4 → trkA	Ngf, Ntrk1	0.02542
PKAc → NR2C	Grin1, Prkaca	0.02542
NR2A:NR2B —PKAc → Ca	Grin1, Prkaca	0.02542
Vitamin D metabolism	Cyp27a1, Cyp2r1	0.02542
Tie2 —p56Dok-2 → PAK1	Dok2, Tek	0.02542
L-tryptophan → 5-hydroxyindoleacetate	Aldh1a7, Maoa, Tph1	0.03438
degradation of tryptophan	Acmsd, Aldh1a7, Maoa, Tph1	0.03625
Csk, CD45 → Lck	Lck, Ptprc	0.04048
NR2B:NR2C —CaMKII → c-Fos	Camk2d, Grin1, Prkaca	0.0436

The results included further pathways which are related to the phosphorylation and desphosphorylation of the β-catenin/E-cadherin complex. In this regard, it has been reported that phosphorylation of β-catenin, e.g., through the epidermal growth factor receptor (EGFR) or the tyrosine-protein kinase Src, leads to the dissociation of the complex and consequently to the accumulation of free β-catenin. On the contrary, dephosphorylation of β-catenin results in the formation of the complex (Müller et al., 1999). Another overrepresented pathway corresponded to nerve growth factor (NGF) signaling via the tyrosine kinase receptor TrkA. NGF has been associated with cancer cell proliferation as well as apoptosis of colon cancer cells (Molloy et al., 2011; Anagnostopoulou et al., 2013) and with angiogenesis (Romon et al., 2010). Further overrepresented pathways related to the angiopoietin-Tie signaling system which plays a role in the regulation of angiogenesis (Fagiani and Christofori, 2013). In tumors, angiopoietin-2 (Ang2) inhibits the activity of the receptor tyrosine kinase Tie2 and destabilizes blood vessels, thereby facilitating angiogenesis (Holash et al., 1999a,b; Augustin et al., 2009). Moreover, several other overrepresented pathways could be linked to anti-tumor properties. These included two p53-dependent pathways which lead to the induction of the cyclin-dependent kinase inhibitor 1 (p21^Cip1^) or the p53 upregulated modulator of apoptosis (Puma), respectively. Downregulation of p21^Cip1^ expression has been associated with poor prognosis and expression of Puma with a rapid apoptosis in CRC (Pasz-Walczak et al., 2001; Yu et al., 2001). Furthermore, the results also included overrepresented pathways which related to vitamin D receptor (VDR) signaling and vitamin D metabolism. VDR signaling is activated upon binding of vitamin D and plays a role in cancer progression as well as cross-talks with multiple other pathways (Slattery, 2007). For example, several studies have suggested interactions of vitamin D or its active vitamin D metabolite, calcitriol, with β-catenin (Deeb et al., 2007; Zheng et al., 2012; Klampfer, 2014). These interactions represent points of convergence between VDR and canonical Wnt signaling in CRC, which has been linked to inhibition of Wnt signaling, tumor growth inhibition, the activation of apoptotic pathways, inhibition of angiogenesis and inhibition of tumor-promoting inflammation (Deeb et al., 2007; Zheng et al., 2012; Klampfer, 2014).

### 3.4. Promoter analysis based on signature genes

Altered gene expression is generally a result of the dysregulated activity of TFs that may play central roles as oncogenes and tumor suppressors. These proteins are often potential targets for cancer therapies due to the fact that many oncogenic signaling pathways involve TFs whose aberrant activation and inactivation contributes to tumor development and progression. We applied a promoter analysis to the previously identified signature genes in order to display which TFs are potentially important regulators in the cell lines under study. This analysis was performed using geneXplain which quantifies the enrichment of TFBSs in promoter regions of the signature genes. In total, 135 and 117 TFs were identified for 1638N-T1 and CMT-93, respectively. These numbers include 51 (Supplementary Table S4) and 33 TFs (Supplementary Table S5) that were exclusively enriched in 1638N-T1 or CMT-93, respectively, as well as 84 overlapping TFs in the intersection between both cell lines (Supplementary Table S6). We exemplarily highlighted several TF families/subfamilies which are present for the three TF sets. In a subsequent analysis, we additionally searched for overrepresented pathways on the basis of these sets.

#### 3.4.1. Intersection-specific TF families/subfamilies of 1638N-T1 and CMT-93

The enriched TFBSs were classified into 32 prominent TF families/subfamilies according to TFClass (Table 3). Our analysis detected several members of the SMAD factor family that were found to have enriched binding sites in the promoters. These factors are a major component of TGF-β signaling which is involved in the regulation of cell growth in the normal intestinal epithelium. Alterations in their expression contribute to cancer aggressiveness in CRC (Xie et al., 2003; Xu and Pasche, 2007; Korchynskyi et al., 1999; Fleming et al., 2013). Furthermore, the analysis revealed overrepresentation for members of the Jun-related factors and Fos-related factors. The protein AP-1 is composed of either Jun-Jun homodimers or Jun-Fos heterodimers and plays a role in differentiation, proliferation, and apoptosis (Ameyar et al., 2003). AP-1 is induced by c-Jun N-terminal protein kinases (JNK) and ERK MAPKs pathways or the canonical Wnt signaling pathway in CRC (Licato et al., 1997; Mann et al., 1999), thereby affecting CRC cell proliferation (Suto et al., 2004). Binding site enrichment was also detected for the CCAAT-enhancer binding protein (C/EBP) family of TFs whose expression has been associated with invasiveness of human colorectal cancer (Rask et al., 2000). Likewise, several members of the POU domain factor family, including Oct-4 (Pou5f1), were found in the intersection between both cell lines. It has been reported that Oct-4 promotes metastasis in CRC through EMT (Dai et al., 2013). Furthermore, Oct-4 knockdown leads to decreased Wnt pathway activity and high risk for liver metastases in CRC patients (Dai et al., 2013). Enrichment for binding sites of VDR, which belongs to the Thyroid hormone receptor-related factor (NR1) family, was also detected in the intersection. It has been suggested that vitamin D has no effect on tumor reduction in APC-deficient mice and that VDR expression is lost in the majority of the colon cancer cells (Giardina et al., 2015). Interestingly, the analysis also revealed enrichment for binding sites of β-catenin which interacts as a cofactor with members of the TCF-7-related factor family to activate Wnt target gene expression (see Supplementary Table S6).

**Table 3:** **Intersection-specific TF families/subfamilies between 1638N-T1 and CMT-93**.

**TF classification**	**TF family/subfamily**
1.1.1	Jun-related factors
1.1.1.1	Jun factors
1.1.1.2	NF-E2-like factors
1.1.2	Fos-related factors
1.1.2.1	Fos factors
1.1.3	Maf-related factors
1.1.3.1	Large Maf factors
1.1.3.2	Small Maf factors
1.1.8	C/EBP-related
1.1.8.1	C/EBP
1.2.1	E2A-related factors
1.2.2	MyoD / ASC-related factors
1.2.2.1	Myogenic transcription factors
1.2.3.1	Tal / HEN-like factors
1.2.6	bHLH-ZIP factors
1.2.6.1	TFE3-like factors
1.2.6.2	USF factors
1.2.6.5	Myc / Max factors
1.2.6.7	Mad-like factors
2.1.2	Thyroid hormone receptor-related factors (NR1)
2.1.2.4	Vitamin D receptor (NR1I)
2.1.3	RXR-related receptors (NR2)
2.1.3.1	Retinoid X receptors (NR2B)
2.1.3.2	HNF-4 (NR2A)
3.1.10	POU domain factors
3.1.10.2	POU2 (Oct-1/2-like factors)
3.1.4	TALE-type homeo domain factors
3.1.4.4	PBX
6.4.1	Runt-related factors
7.1.1	SMAD factors
7.1.1.1	Regulatory Smads (R-Smad)
7.1.1.3	Repressor-Smads (I-Smad)

#### 3.4.2. Overrepresented TRANSPATH pathways based on intersection-specific TFs

Based on the 84 overlapping TFs in the intersection of both cell lines, the pathway analysis revealed overrepresentation for 35 TRANSPATH pathways (Table 4). Members of the SMAD factor family were found to be involved in many of the top overrepresented pathways. In this context, the TGF-β pathway was detected among the most overrepresented pathways. Likewise, SMADs were also found to be involved in a pathway which corresponded to the regulation of endothelin-1 (ET-1). ET-1 is a vasoconstrictor peptide, which is known to be produced by CRC cells and stimulates CRC proliferation (Asham et al., 2001; Grant et al., 2007; Knowles et al., 2012). The second most overrepresented pathway corresponded to the transcriptional regulation of ECM components. ECM sustains normal tissue homeostasis and prevents malignant transformation (Gao et al., 2014). Its anti-tumor properties are opposed by chronic inflammation, which may lead to the conversion of a tumor-inhibiting into a tumor-promoting microenvironment (Gao et al., 2014).

**Table 4:** **Overrepresented TRANSPATH pathways based on the intersection-specific TF set of 1638N-T1 and CMT-93**.

**Pathway**	**Hit names of TFs**	***P*-value**
Endothelin-1 gene regulation	Fos, Jun, Smad3, Smad4	2.8851480825350153E-8
Transcriptional Regulation of ECM components	Smad2, Smad3, Smad4, Tfe3	4.2462045176114295E-7
PPAR pathway	Ppara, Pparg, Rxra, Rxrb, Smad2, Smad3	4.401281772213993E-7
BMP7 → Smad1, Smad5, Smad8	Smad1, Smad4, Smad5, Smad9	9.814052909861147E-7
TGFbeta pathway	Fos, Jun, Pparg, Smad1, Smad2, Smad3, Smad4, Smad5, Smad7, Smad9, Tfe3	1.1668767789940478E-6
SMAD7, SIK1 gene induction	Smad2, Smad3, Smad4	2.3427402430218484E-6
MIC2 signaling	Fosb, Jun, Jund, Srf	8.907929442840803E-6
Smad2/3, PPARgamma, regulation of bioavailability	Pparg, Smad2, Smad3	9.284406529601274E-6
MIC2-isoform2 —JNK, JunD → MMP9	Fosb, Jun, Jund	4.556873521617853E-5
TGFbeta1 → Smad1, Smad2, Smad5	Smad1, Smad2, Smad5	7.900923266022097E-5
MIC2-isoform2 —FosB → MMP9	Fosb, Jund, Srf	1.2524823045846698E-4
mammalian Hippo network	Smad2, Smad3, Smad4, Smad7, Tead1	1.609311066368507E-4
RA, 15d-PGJ2 → RXR-beta, PPAR-gamma	Pparg, Rxrb	1.830040551373434E-4
RXR-beta, VDR heterodimerization	Rxrb, Vdr	1.830040551373434E-4
Smad2/3 —TAZ → cytoplasmic retention	Smad2, Smad3, Smad4	3.5891749789138236E-4
Sox9 —Smad3 → COL2A1	Smad2, Smad3	5.443266849267895E-4
MyoD regulation	Myod1, Tcf3	5.443266849267895E-4
MKK4 —/ PPAR-gamma	Pparg, Rxrb	0.0010793689633243411
Ctbp1 —/ Smad3	Smad3, Smad4	0.0010793689633243411
ERK1 → NQO1	Mafk, Nfe2l2	0.0010793689633243411
E2F —/ Smad4	Smad3, Smad4	0.0017836171536470523
Nrf2 → HMOX1	Mafk, Nfe2l2	0.0017836171536470523
stress-associated pathways	Jun, Mitf, Myf6, Pparg, Rxra, Rxrb	0.0023269497130444508
PRIC complex → PPAR-alpha	Ppara, Rxra	0.002652641362864685
TGFbeta1 → Smad2/3	Smad2, Smad3	0.002652641362864685
MEK → EZR	Fos, Jun	0.002652641362864685
p38 pathway	Jun, Mitf, Myf6, Pparg	0.0034193798154062926
15-Keto-PGE2 → TP63	Pparg, Smad2	0.003682094214938695
TGFbetaR-I —pak2, ERK1 → SMAD7, SERPINE1	Smad2, Smad3, Smad4	0.003904012718560197
15d-PGJ2 → PPAR-gamma	Pparg, Rxra	0.009320059910591498
Regulation of mesendoderm differentiation genes	Smad2, Smad4	0.012994431232912744
IRAK-1 —MKK3 → TNF	Fos, Jun	0.015031819490714783
JNK pathway	Jun, Pparg, Rxra, Rxrb	0.016302058148038395
VDR network	Rxra, Rxrb, Vdr	0.01758202640044028
TGFbetaR-I → ERK	Smad2, Smad3	0.04188993264127895

Furthermore, the analysis showed overrepresentation for a PPAR-related pathway which comprises the peroxisome proliferator activated receptors PPAR-α, PPAR-γ and Smads. It was shown that activation of PPAR-γ inhibits TGF-β-induced loss of E-cadherin expression, the induction of mesenchymal markers (vimentin, N-cadherin, fibronectin), MMPs and antagonizes Smad3 function, thereby preventing metastasis in lung cancer (Reka et al., 2010). This pathway has also been implicated in the induction of apoptosis as well as inhibition of cyclooxygenase-2 (COX-2) in CRC (Yang and Frucht, 2001). Activation of the PPAR pathway was shown to cause reduction in linear and clonogenic growth and, thus, it has been suggested that PPAR-γ modulates cell growth and differentiation of CRC cells (Sarraf et al., 1998). Moreover, it was shown that PPAR-γ expression is altered in APC-deficient mice, an effect which is thought to be mediated by the Wnt/β-catenin pathway (Jansson et al., 2005). In conformity with the overrepresented pathways, which were found based on the signature genes of CMT-93, a VDR network-related pathway was also found based on the intersection-specific TFs.

#### 3.4.3. 1638N-T1-specific TF families/subfamilies

The enriched TFBSs can be classified into 14 prominent TF families/subfamilies based on the 1638N-T1-specific TFs (Table 5). Amongst these, the factors Onecut1 and Onecut2, which belong to the HD-CUT factors family, were found to be enriched in the signature genes of 1638N-T1. Through targeting of Onecut2, the microRNA miR-429 has been reported to regulate the expression of several EMT-related markers (Sun et al., 2014b). Overall, it has been suggested that Onecut2 is involved in EMT, migration and invasion of CRC cells (Sun et al., 2014b). Onecut1 (Hnf6) expression was found to be positively correlated with the expression of p53 and E-cadherin in human lung cancer. The Onecut1-mediated induction of p53 is thought to inhibit EMT, migration and invasion (Yuan et al., 2013). Moreover, the analysis detected the HOX-related factors Cdx1 and Cdx2, which regulate intestine-specific gene expression and enterocyte differentiation (Suh et al., 1994; Suh and Traber, 1996; Taylor et al., 1997; Freund et al., 1998; Soubeyran et al., 1999; Lynch et al., 2003). In addition, it has been suggested that expression of Cdx1 reduces cancer cell proliferation by reducing cyclin D1 expression (Lynch et al., 2003). Interestingly, Cdx1 and Cdx2 also inhibit proliferation of CRC cells by blocking canonical Wnt signaling activity (Guo et al., 2004). In contrast, another study indicated that Cdx2 can promote expression of Wnt/β-catenin pathway genes (da Costa et al., 1999). Furthermore, the analysis revealed overrepresentation for several members of the interferon regulatory factor (IRF) family. Most IRFs play central roles in immune response, apoptosis and are known to exhibit tumor suppressor properties in cancer (Bouker et al., 2005). For example, anti-tumor function of IRF-1- and IRF-5-associated pathways have been suggested in CRC (Hu and Barnes, 2006; Yuan et al., 2015). The analysis also detected Sox9, a member of the SOX-related factors. Sox9 is a target as well as potential upstream regulator of Wnt signaling (Blache et al., 2004; Bastide et al., 2007).

**Table 5:** **1638N-T1-specific TF families/subfamilies**.

**TF classification**	**TF family/subfamily**
3.1.1.9	CDX (Caudal type homeobox)
3.1.9	HD-CUT factors
3.1.9.1	ONECUT
3.1.10.7	HNF1-like factors
3.3.1	Forkhead box (FOX) factors
3.3.1.1	FOXA
3.3.1.6	FOXF
3.5.3	Interferon-regulatory factors
4.1.1	SOX-related factors
4.1.1.3	Sox-related factors, Group C
4.1.1.4	Sox-related factors, Group D
4.1.1.5	Sox-related factors, Group E
6.1.3	NFAT-related factors
8.2.1	HMGA factors

#### 3.4.4. Overrepresented TRANSPATH pathways based on 1638N-T1-specific TFs

In total, 7 overrepresented pathways were found based on the 51 exclusive TFs for 1638NT-1 (Table 6). The results included overrepresented pathways which corresponded to the TLR (Toll-like receptor) pathways TLR3, TLR4, and TLR9. TLRs are pattern recognition receptors (PRRs) that play key roles in innate and adaptive immune responses. In host defence, TLRs recognize pathogens by pathogen-associated molecular patterns (PAMPs). TLRs are involved in inflammatory reponses, cell proliferation and survival, and have been associated with pro-tumor as well as anti-tumor effects in cancer (Rakoff-Nahoum and Medzhitov, 2009; Basith et al., 2012). TLR signaling pathways promote the production of cytokines and chemokines via interfering with intracellular pathways and activation of TFs, such as IRFs and NF-κB (Li et al., 2014). In particular, activation of the TLR9 pathway promotes the development of anti-tumor T-cell responses (Krieg, 2008). In contrast, it was also shown that this pathway can promote angiogenesis and cancer progression (Belmont et al., 2014; Holldack, 2014). TLR3 activation mediated by dsRNA was shown to trigger apoptosis of human breast cancer cells (Salaun et al., 2006). Additionally, signaling by IRF-3 has been implicated in TLR3-mediated apoptosis in prostate cancer (Gambara et al., 2015). Another overrepresented TRL-related pathway corresponded to the lipopolysaccharide (LPS)-induced activation of the TFs IRF-3, IRF-7, and CBP/p300 via the TLR4/MD2 complex. Moreover, it was shown that metastasis of CRC cells is increased through a signaling cascade involving LPS-induced TLR4 signaling as well as downstream PI3K/Akt signaling and β1 integrin activity (Hsu et al., 2011). LPS also increases phosphorylation of Mapk1 and p38, activation of NF-κB, and promotes cytokine production, such as that of IL-8, vascular endothelial growth factor (VEGF), and TGF-β in human colon cells (Tang and Zhu, 2012). Moreover, the same study has implicated TLR4 in promoting immune escape of the human colon cancer cells by inducing immunosuppressive factors and apoptosis resistance (Tang and Zhu, 2012). Strikingly, two pathways corresponded to the canonical Wnt/β-catenin signaling pathway which is of high relevance in CRC.

**Table 6:** **Overrepresented TRANSPATH pathways based on the 1638N-T1-specific TF set**.

**Pathway**	**Hit names of TFs**	***P*-value**
dsRNA → IRF-7:IRF-3:CBP:p300	Irf3, Irf7	3.947146928237511E-4
LPS → IRF-3:IRF-7:CBP:p300	Irf3, Irf7	0.0014260355781928803
wnt → beta-catenin	Ctnnb1, Tbp	0.005286143325503229
TLR9 pathway	Irf1, Irf7	0.0106622039857671
TLR3 pathway	Irf3, Irf7	0.01598971078797065
wnt pathway	Ctnnb1, Tbp	0.02936961872680831
TLR4 pathway	Irf3, Irf7	0.03155510304218106

#### 3.4.5. CMT-93-specific TF families/subfamilies

The enriched TFBSs can be classified into 10 prominent TF families/subfamilies for the CMT-93-specific TFs (Table 7). The results included Ebf3 which is a member of the Early B-Cell Factor-related factors family. This family plays a role in differentiation of specific cell types such as B lymphocytes and olfactory cells (Zhao et al., 2006). Expression of Ebf3 was previously shown to promote cell cycle arrest and apoptosis in several tumor cell lines including colon carcinoma (Zhao et al., 2006).

**Table 7:** **CMT-93-specific TF families/subfamilies**.

**TF classification**	**TF family/subfamily**
1.1.2	Fos-related factors
1.2.6.3	SREBP factors
2.3.3.1	GLI-like factors
3.5.2	Ets-related factors
3.5.2.1	Ets-like factors
3.5.2.2	Elk-like factors
3.5.2.3	Elf-1-like factors
6.1.1	NF-kappaB-related factors
6.1.5	Early B-Cell Factor-related factors
6.2.1	STAT factors

The analysis also reported enriched TFBSs for the NF-κB-related factor family. NF-κB signaling is usually induced by inflammation and also known to be triggered by cancer progression. Many recent findings indicate that NF-κB is constitutively activated in malignant cells of various cancers including CRC (Nakshatri et al., 1997; Wang et al., 1999; Lindholm et al., 2000; Lind et al., 2001; Kojima et al., 2004), thereby promoting, cell proliferation, angiogenesis, metastasis, upregulation of chemokine secretion and other anti-apoptosis proteins (Sakamoto et al., 2009; Wang et al., 2009). Furthermore, enriched binding sites were detected for the signal transducer and activator of transcription (STAT) family which are critical regulators of immune and inflammatory responses (Yu et al., 2009). These factors play an important role in many types of cancer, including colorectal cancer, as they may promote pro-tumor inflammatory pathways such as NF-κB and JAK/STAT pathways, as well as suppress anti-tumor immunity (Wang et al., 2009; Yu et al., 2009; Slattery et al., 2013). The activation of Stat3 and Stat5 has been shown to promote cell proliferation and invasion in cancer (Yu et al., 2009), while Stat3 was also found to be persistently activated and overexpressed in colon cancers (Klampfer, 2008). Our analysis also revealed binding site enrichment for several members of the family of Ets-related factors which are involved in diverse cellular processes, thereby often cooperatively interacting with other TFs and co-factors (Oikawa, 2004). In cancer, this family is known to regulate genes which play a role in angiogenesis, invasion and metastasis. Therefore, their altered expression has been implicated in development and progression of cancer (Bassuk and Leiden, 1997; Graves and Petersen, 1998; Oikawa and Yamada, 2003; Oikawa, 2004). Moreover, it has been suggested to use ETS-related factors as prognostic markers in cytotoxic treatment of metastatic colorectal cancer (Giessen et al., 2013).

#### 3.4.6. Overrepresented TRANSPATH pathways based on CMT-93-specific TFs

In total, 52 overrepresented pathways were found based on the 33 exclusive TFs for CMT-93 (Table 8). Most of these overrepresented pathways involved NF-κB family members. Further overrepresented pathways involved the tumor necrosis factor-alpha (TNF-α) of which one related to the TNF-α-mediated activation of NFκB. An increase in production of the pro-inflammatory cytokine TNF-α is linked to poor outcome in CRC (Balkwill2005, Mantovani2005, Coussens2002, Balkwill2001). Interestingly, TNF-α was shown to promote Wnt signaling through translocation of β-catenin into the nucleus in gastric tumor cells (Oguma et al., 2008).

**Table 8:** **Overrepresented TRANSPATH pathways based on the CMT-93-specific TF set**.

**Pathway**	**Hit names of TFs**	***P*-value**
PDGF B → STATs	Stat3, Stat5a, Stat5b	6.272149884041184E-7
STAT5 → Ccnd1	Stat5a, Stat5b	3.1953088992325076E-5
STAT5 → CISH	Stat5a, Stat5b	3.1953088992325076E-5
STAT5 → CSN2	Stat5a, Stat5b	3.1953088992325076E-5
PDGF B → STAT1alpha, STAT5	Stat5a, Stat5b	9.554461322021479E-5
importin-alpha3 → NFkappaB	Nfkb1, Rela	9.554461322021479E-5
Pin1 → p50:RelA-p65	Nfkb1, Rela	9.554461322021479E-5
Epo —Jak2 → STAT5	Stat5a, Stat5b	1.9046201145220444E-4
Epo → STAT5	Stat5a, Stat5b	1.9046201145220444E-4
IL-3 → STAT5	Stat5a, Stat5b	3.1639480397985514E-4
LXR —/ IL1B	Nfkb1, Rela	3.1639480397985514E-4
SOCS-1 → p50:RelA-p65	Nfkb1, Rela	4.730345816689199E-4
TLR8 —Btk → NF-kappaB	Nfkb1, Rela	4.730345816689199E-4
TLR9 —Btk → NF-kappaB	Nfkb1, Rela	4.730345816689199E-4
p50:RelA-p65 → SELE	Nfkb1, Rela	4.730345816689199E-4
IFNalpha/beta pathway	Stat3, Stat5a, Stat5b	6.440779144960161E-4
fMLP → NFkappaB	Nfkb1, Rela	6.600749950470129E-4
IL-2 → STAT5	Stat5a, Stat5b	6.600749950470129E-4
IFNalpha, IFNbeta → STAT5	Stat5a, Stat5b	6.600749950470129E-4
LXR network	Nfkb1, Rela	6.600749950470129E-4
IL-2 - STAT5 pathway	Stat5a, Stat5b	8.772117434506635E-4
cPKC —CARD9 → TRAF6	Nfkb1, Rela	8.772117434506635E-4
mannan, Dectin2	Nfkb1, Rela	8.772117434506635E-4
EDA-A2 —TRAF3 → p50:RelA-p65	Nfkb1, Rela	0.0011241425642107344
EDA-A1 → p50:RelA-p65	Nfkb1,Rela	0.0011241425642107344
IL-1 pathway	Elk1, Nfkb1, Rela	0.0012748952830245175
neurotrophic signaling	Elk1, Nfkb1, Rela, Trp53	0.0012979014272467505
NGF —p75NTR → p50:RelA-p65	Nfkb1, Rela	0.001400567221887963
CH000000333	Nfkb1, Rela	0.0017061874975544628
EDAR → NF-kappaB	Nfkb1, Rela	0.0017061874975544628
TNF-alpha → p50:RelA-p65	Nfkb1, Rela	0.0024038320457071337
PDGF pathway	Stat3, Stat5a, Stat5b	0.004038573581262634
TBK1:TRIF:IKK-i → p50:RelA	Nfkb1, Rela	0.004136568270131099
dsRNA → p50:RelA	Nfkb1, Rela	0.004136568270131099
RANKL → p38	Nfkb1, Rela	0.004638374899213325
LAT → p50:RelA	Nfkb1, Rela	0.005722351182439321
EDAR pathway	Nfkb1, Rela	0.009597224599851443
T-cell antigen receptor pathway	Elk1, Nfkb1, Rela	0.009985935589865625
LPS → NF-kappaB	Nfkb1, Rela	0.011871624770568048
NF-kappaB → genes encoding endothelial adhesion molecules	Nfkb1,Rela	0.011871624770568048
Epo pathway	Stat5a, Stat5b	0.012677905293747096
TLR9 pathway	Nfkb1, Rela	0.012677905293747096
IL-1beta → p50:RelA	Nfkb1, Rela	0.01436112389208374
TLR3 pathway	Nfkb1, Rela	0.018969411877391994
TNFR1 signaling	Nfkb1, Rela	0.019957669453188952
diacyl lipopeptide, TLR2	Nfkb1, Rela	0.019957669453188952
p38 pathway	Stat3, Trp53	0.029796036656231952
PRL pathway	Stat5a, Stat5b	0.03343465884776058
p50:RelA-p65 → IL8	Nfkb1, Rela	0.03343465884776058
IL-3 signaling	Stat5a, Stat5b	0.03343465884776058
TLR4 pathway	Nfkb1, Rela	0.037242517103675384
TLR2-mediated signaling	Nfkb1, Rela	0.04394876054564345

In conformity with the results obtained for the 1638N-T1-specific TFs, TLR-related pathways for five different TLRs (TLRs 2,3,4,8,9) were also detected for the TFs of the CMT93-specific set. The results further included several overrepresented STAT factors-related pathways that included an activation of STATs by platelet-derived growth factor (PDGF)-mediated signaling. Signaling via PDGF tyrosine kinase receptors plays an important role in angiogenesis, mesenchymal cell migration, proliferation and the expression and activation of PDGF receptors is particularly associated with invasion and metastasis in CRC (Yu et al., 2003; Kitadai et al., 2006; Steller et al., 2013).

Moreover, the analysis detected overrepresentation for LXR-related pathways that implicate a role for NFκB subunits RELA/p65, NFKB1/p105, NFKB1/p50 as well as interleukin-1 beta (IL-1β). Interestingly, the signature gene set for CMT-93 included the factors Nr1h2 and Nr1h3, two members of the thyroid hormone receptor-related factor (NR1) family. These genes encode liver X receptors (LXRs), of which the oxysterol receptor LXRα (Nr1h3) is thought to increase caspase-dependent apoptosis, slow growth of xenograft tumors in CRC mouse models and may negatively interfere with Wnt signaling through direct binding to β-catenin in CRC (Uno et al., 2009; Sasso et al., 2013). Hence, LXRs have been considered important potential targets in cancer therapeutics on account of their tumor suppressor activities (Sasso et al., 2013; Vedin et al., 2013; Lin and Åke Gustafsson, 2015). With respect to IL-1β, this pro-inflammatory cytokine has been associated with angiogenesis, invasiveness of different tumor cells and increased risk of CRC (Voronov et al., 2003; Andersen et al., 2013).

### 3.5. Identification of upstream master regulators in pathways based on TF sets

In the previous step, we reported potentially important TFs for the sets of signature genes, on the basis of which we defined sets of TFs for the intersection between the two cell lines as well as for the 1638N-T1-specific and CMT-93-specific TFs. Since signal transduction pathways can modulate the activity of nuclear TFs, activation mutations in these pathways can lead to the altered expression of the TFs and their target genes. These pathways are diverse in both their complexity and the mechanism of signal transduction, and even more complexity is added through cross-talks or transactivation signals between different pathways. Therefore, we were interested in the detection of upstream regulators, called master regulators, for the previously defined TF sets. We additionally aimed to construct the upstream pathways which may regulate activity or inhibition of the TFs.

We applied the master regulator analysis from geneXplain to each of the three TF sets, namely the intersection with overlapping TFs between 1638N-T1 and CMT-93, the 1638N-T1-specific and the CMT-93-specific TFs. This workflow will first map the set-specific TFs to TRANSPATH molecules and then search based on the TRANSPATH knowledge for upstream master regulators. We report the top three master regulators for each TF set (Table 9) and provide references for their roles in cancer. Noteworthy, we only proposed distinct master regulators for each gene set, i.e., different splice variants or isoforms of a master regulator reported by the analysis were counted as the same master regulator.

**Table 9:** **Top three master regulators for three TF sets: Intersection-specific TFs of the two cell lines, 1638N-T1-specific TFs and CMT-93-specific TFs**.

**Rank**	**Intersection set**	**1638N-T1-specific set**	**CMT-93-specific set**
1	Rad23A	MLK3	Aebp1 (ACLP)
2	Smad3	TBK1	Il2rg (gamma-c)
3	Melk	Siah2	Mapk1 (ERK2)

The master regulators and their pathways, denoted as master regulator pathways, constitute the set-specific TFs which are either connected to other set-specific TFs or intermediate molecules. These intermediate molecules are not contained within the respective TF sets but function as a bridge between the set-specific TFs and the master regulator(s) in the pathways. Since the pathways of the top ranked master regulators share many of the interacting nodes and, thus, are very similar to each other, we merged the top 3 master regulator pathways for each set into one network.

#### 3.5.1. Prediction of master regulators and construction of a master regulatory network based on the intersection-specific TF set

For the intersection-specific TF set, we obtained the three master regulators Rad23A, Smad3, and Melk that reach 91, 74, and 93 TFs from the set, respectively. The master regulator Rad23A is involved in DNA damage recognition and nucleotide-excision repair. A recent study has implicated Rad23A in nuclear translocation of the apoptosis-inducing factor (AIF) during induction of cell death (Sudhakar and Chow, 2014). However, not much is known about its specific function in CRC.

As a major component of the TGF-β signaling pathway, the Smad3 master regulator plays a pivotal role in survival, invasion, and metastasis of CRC cells (Xu and Pasche, 2007; Fleming et al., 2013). However, despite the fact that not much is known about the pathogenic role of Smad3, mutations in the gene occur rather rarely in human CRC (Ku et al., 2007). Loss of Smad3 has been associated with metastasis in CRC, an outcome that is thought to be dependent on chronic inflammation, e.g., triggered by bacterial infection (Zhu et al., 1998; Maggio-Price et al., 2006).

The third master regulator maternal embryonic leucine zipper kinase (Melk) is a known embryonic and neural stem cell marker and belongs to the family of serine/threonine kinases (Choi and Ku, 2011). Melk is normally expressed in cells that undergo proliferation during embryonic development, however, elevated expression has been particularly observed in variety of different cancer cell types including colorectal cancer (Gray et al., 2005; Badouel et al., 2010; Ganguly et al., 2015). Moreover, it has been shown that Melk knockdown decreases proliferation and tumor growth in CRC and, thus, it has been proposed to use Melk as a therapeutic target for cancer (Gray et al., 2005).

The merged master regulatory network consisted of 155 nodes (Figure 2, Supplementary Table S7 and Supplementary Figure S7). The master regulators Rad23A and Smad3 were found most upstream in the hierarchy of the network. Rad23A was connected via the nodes p300 and CBP to the other nodes in the network, whereas Smad3 was connected to a variety of nodes which also included important cancer-associated TFs such as c-Myc, Runt-related factors, and Smad factors. Likewise, the master regulator Melk featured cascades through several molecules including Smad factors and p53 (see Figure 2 for more details).

#### 3.5.2. Prediction of master regulators and construction of a master regulator network based on the 1638N-T1-specific TF set

The master regulator analysis detected Mlk3, Tbk1 and Siah2, which reach 28, 22, and 37 TFs from the 1638N-T1-specific set, respectively. The first master regulator MLK3 is a serine/threonine kinase that activates p38 MAP kinase, ERK, and JNK signaling pathways (Velho et al., 2014). MLK3-mediated activation has been shown to promote invasion and metastasis in several cancer types, including breast and gastric cancers (Chen et al., 2010; Mishra et al., 2010; Chen and Gallo, 2012; Cronan et al., 2012). Moreover, it has been proposed that mutant MLK3 is involved in the deregulation of several important CRC-associated signaling pathways such as WNT, MAPK, NOTCH, TGF-β, and P53 (Velho et al., 2014). Concerning Wnt signaling pathways in MLK3 mutant cells, it has been shown that components of the canonical Wnt pathway were found to be downregulated, while components of the non-canonical planar cell polarity (PCP) pathway were found to be upregulated.

The proposed master regulator TBK1 is a member of the non-canonical IκB protein kinases which is involved in the activation of IRF3 and c-Rel and NF-κB in cancer. The role of TBK1 is poorly investigated in CRC. However, several studies associated TBK1 with malignant transformation, cell growth and proliferation (Chien et al., 2006; Kim et al., 2013a,b).

The third master regulator Siah2 is an E3 ubiquitin ligase that regulates the degradation of a variety of substrates such as the nuclear corepressor (N-CoR), TRAF2, 2-oxoglutarate dehydrogenase-complex protein E2 (OGDC-E2), TIEG, and β-catenin (Zhang et al., 1998; Matsuzawa and Reed, 2001; Habelhah et al., 2002, 2004; Johnsen et al., 2002). Siah2 has been implicated in MAPK signaling, mitochondrial dynamics and cell survival (Nakayama et al., 2009; Kim et al., 2011). In addition, several studies have indicated that Siah2 functions as a proto-oncogene, while the Siah1 isoform has been associated with tumor suppressor activity (Wong and Möller, 2013; Gopalsamy et al., 2014). Although its role in CRC remains unclear, Siah2 has been suggested to promote invasion and metastasis in a variety of other cancers, including prostate, breast and liver (Qi et al., 2010, 2013; Behling et al., 2011; Malz et al., 2012; Sarkar et al., 2012; Wong et al., 2012; Gopalsamy et al., 2014).

The merged master regulatory network consisted of 52 nodes (Figure 3, Supplementary Table S8 and Supplementary Figure S8). MLK3 and Siah2 were found most upstream in the hierarchy of the network, whereas TBK1 was found downstream of the network branch which is regulated by Siah2. MLK3 featured cascades through MKK3-isoform1, 4, and 6, and IKK-alpha-isoform1, and -beta. Siah2 was connected via the molecule alpha-synuclein-isoform1, Ubc5A, B, and C. TBK1 was connected via IRF3, 5, and 7, STAT6, and IKK-beta to its downstream nodes.

#### 3.5.3. Prediction of master regulators and construction of a master regulator network based on the CMT-93-specific TF set

For the CMT-93-specific TFs, the analysis reported the master regulators Aebp1 (ACLP), Il2rg (gamma-c) and Mapk1 (ERK2), which reach 43, 36, and 31 TFs from the set, respectively. The first proposed master regulator, Aebp1, is known to act as a transcriptional repressor in adipogenesis (Ladha et al., 2012). Aebp1 is upregulated in the majority of the primary glioblastoma multiforme (GBM) and loss of Aebp1 function was shown to result in apoptosis (Ladha et al., 2012). Moreover, Aebp1 induces NF-κB activity which leads to macrophage inflammatory responsiveness and affects tumor cell growth and survival (Majdalawieh et al., 2007). In the context of breast cancer tumorigenesis, Aebp1 has been suggested to be involved in the regulation of the cross-talk between mammary epithelium and stroma (Holloway et al., 2012). To this date, the role of Aebp1 remains largely unclear in CRC.

The second master regulator corresponded to the interleukin 2 receptor subunit gamma (Il2rg/gamma-c) which heterodimerizes with several interleukin receptors, including receptors for the interleukins −2, −4, −7, −9, −15, and −21 (Nata et al., 2015). Interleukins receptor signaling pathways are known to play crucial roles in inflammation-dependent progression and anti-tumor responses in CRC (West et al., 2015).

The last master regulator Mapk1 (ERK2) belongs to the MAP-kinases, which regulate cell growth, differentiation, proliferation, migration, and apoptosis (Santarpia et al., 2012). MAPKs act downstream of several growth-factor receptors such as Egfr, which are often found overexpressed and activated in CRC (Fang and Richardson, 2005). Thus, it has been stated that the ERK MAPK pathway plays a central role in the progression of CRC (Fang and Richardson, 2005). In addition, it has been proposed that this pathway but not the JNK pathway or the p38 MAPK pathway is the key regulator of cell proliferation in CRC (Fang and Richardson, 2005).

The merged master regulatory network was composed of 65 nodes (Figure 4, Supplementary Table S9 and Supplementary Figure S9). ACLP (Aebp1) and Il2rg (gamma-c) were found to be the regulators most upstream in the network. ACLP (Aebp1) was connected via the nodes ERK1 and TNF-alpha to the other nodes in the network. The master regulator Il2rg (gamma-c) featured a cascade through Jak3-isoform1, whereas the master regulator Mapk1 (ERK2) was connected to several molecules and TF families, including SREBP factors, STAT factors and Ets-like factors (see Figure 4 for more details).

#### 3.5.4. A comparison with randomly selected gene sets

To test the prediction quality of our results and, whether they are specific for CRC, we performed a comparison between our results and those found for randomly drawn gene sets. Thus, we first randomly selected 10 gene sets, each of which had the same sample size as the signature genes analyzed in this study. After that, each random gene set was analyzed in the same way as both signature gene sets. In this regard, we started with TFBS enrichment analyses (see Section 2.4.1) for the detection of enriched TFBSs in the promoter regions of each random gene set. After retrieving the corresponding TFs, we observed that 17 TFs were common to each of the 10 random gene sets. Interestingly, 13 out of these 17 TFs were also detected based on both CRC signature gene sets (see Section 3.4.1). To determine their potential role in the context of our results, we further searched for overrepresented TRANSPATH pathways and master regulators based on these 13 TFs (see Section 2.4.2 and 2.4.3). The results of these analyses showed that there were no overrepresented pathways and, beyond that, the master regulators were completely different from those presented in Section 3.5.1, 3.5.2, and 3.5.3. Finally, we searched for overrepresented TRANSPATH pathways based on each random gene set (see Section 2.3.2). As expected, the overrepresented pathways found for each random gene set were completely different among themselves and, thus, they have no overlap with the pathways presented in the Section 3.3.1 and 3.3.2.

## 4. Discussion

In this study, we specifically focused on revealing the similarities and differences with respect to the transcriptional regulation as well as the pathway repertoire of two distinct colorectal cancer (CRC) cell lines, namely 1638N-T1 and CMT-93, in a direct comparison. Based on signature genes that are most significantly upregulated in cancer cell type I and cancer cell type II, respectively, our approach aimed to identify the upstream transcriptional regulators and their regulatory networks.

Our results indicated that many of the pathways, which were identified based on the signature genes, can be linked to both pro-tumor as well as anti-tumor properties. In particular, we found pathways for 1638N-T1 which play a role in the detoxification of carcinogens, immune response, and apoptosis. Additionally, we found pathways which can be linked to oxidative stress, inflammation, cell migration, proliferation and survival. Oxidative stress is one important environmental factor in cancer as it is genotoxic and contributes to mutations (Beckman and Ames, 1997). During tumor progression, cells harbor mutations that reduce growth-limiting effects in pathways such as TGF-β signaling which becomes a tumor-promoting pathway due to mutations in later stages of CRC (Jakowlew, 2006; Bellam and Pasche, 2010; Calon et al., 2012). Therefore, it is likely that the results include many putative anti-tumor pathways that contain mutations in the cell lines, which is an important aspect to be addressed in future investigations.

On the level of transcriptional regulation, we identified a number of well-known, cancer-associated TFs with significantly enriched binding sites in the promoter regions of the signature genes. These TFs belong to a variety of TF families/subfamilies and are known to form protein-protein interactions with each other such as Jun factors and Fos factors which form the heterodimeric AP-1 protein (Chen et al., 1996; Shaulian and Karin, 2002; Eferl and Wagner, 2003). Likewise, nuclear receptors (NRs) of the subfamilies vitamin D receptors (NR1I) and retinoid X receptors form the VDR-RXR heterodimer complex (Orlov et al., 2012) that has been implicated in anticancer therapeutics (Friedrich et al., 2002; Sepulveda et al., 2006; Deeb et al., 2007; Matsuda and Kitagishi, 2013). In this light, it is known that TFs do not regulate their target genes in solitude, but interact with other TFs and cofactors in specific combinations for a fine-tuned control of gene expression (Gerstein et al., 2012). In addition, we identified different TF families/subfamilies that have overlapping binding sites and may act in a synergistic, additive, or antagonistic fashion in cancer. Kittler et al. revealed binding redundancy for NRs and their putative cooperating TFs in breast cancer on the basis of 39 factors, whereas non-overlapping binding sites were found to occur rarely (Kittler et al., 2013). Taken together, although the signature genes of both cell lines show no overlap, they may still be regulated by common factors in CRC.

We revealed that 62 and 72% of the TFs for 1638N-T1 and CMT-93, respectively, were found in the intersection of both cell lines. Consequently, only 38 and 28% of the TFs were exclusive for 1638N-T1 and CMT-93, respectively, whose implications in signal transduction pathways might explain phenotypic differences between the two cell lines with regard to tumor growth and metastasis. We deduced cross-talks between several pathways that might have an impact on tumor progression in the cell lines. For the APC-deficient 1638N-T1 cell line, we found overrepresented pathways which related to the activation of the canonical Wnt signaling pathway (Tables 1, 6). Wnt signaling activity is known to contribute to tumor aggressiveness; therefore, it is often targeted in cancer therapy (Anastas and Moon, 2013; Loh et al., 2013). It has also been stated that enhancement of canonical Wnt signaling activity is required for tumor progression and metastasis (Oguma et al., 2008). On the other hand, we showed several pathways for CMT-93 which have been previously associated with an inhibition of Wnt signaling. Two of these pathways related to VDR signaling and LXR-induced signaling (Tables 4, 8). Strikingly, VDR and LXRα (Nr1h3) were included in the signature genes for CMT-93 (see Supplementary Table S2), and VDR also showed significantly enriched binding sites (see Supplementary Table S5). Previous studies have investigated the activation of VDR as well as LXR in APC-deficient mice and observed that the activity of both factors decreased tumor growth (Zheng et al., 2012; Sasso et al., 2013). In addition, LXR expression was found to be downregulated in colon tumors of APC-deficient mice compared with adjacent normal mucosa (Su et al., 1992; Sasso et al., 2013). We also found that CTNNB1, which encodes β-catenin, was not included in the signature genes of any of the two cell lines, but showed significant binding site enrichment (see Supplementary Table S6). With respect to TCF-7-related factors, the genes Tcf7l1 and Lef1, however, were included in the signature genes of 1638N-T1. Interestingly, VDR and LXR can both directly bind to β-catenin, thereby preventing β-catenin from binding to its target sites (Uno et al., 2009; Makoukji et al., 2011; Zheng et al., 2012; Larriba et al., 2013; Shackleford et al., 2013; Lim et al., 2014).

**Figure 5:**
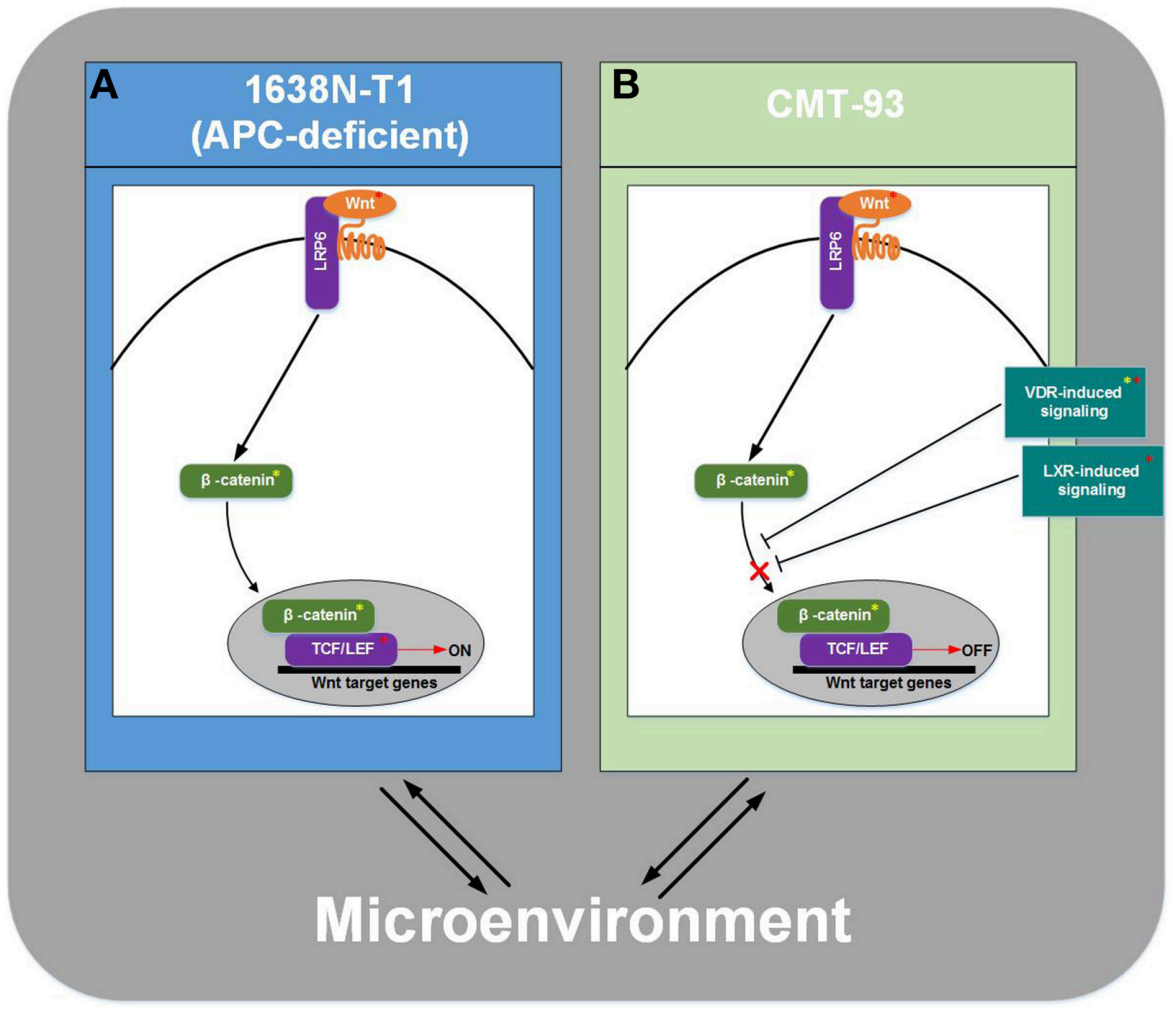
**Schema for potential state of canonical Wnt signaling pathway in mouse models**. **(A)** Wnt signaling is activated in 1638N-T1. **(B)** Wnt signaling is inhibited in CMT-93 through cross-talks with VDR- and/or LXR-induced pathways. Interaction of tumor cells with the microenvironment has an impact on cell proliferation, invasion, and metastasis in mouse models. Signature genes and transcription factors/cofactors, whose binding sites were found to be enriched in promoters, are indicated by a red asterisk or a yellow asterisk, respectively.

All things considered, supported by the knowledge that 1638N-T1 cells harbor a mutation in the APC gene, which leads to aberrant Wnt pathway activation: we suggest that Wnt signaling is activated in 1638N-T1, but inhibited in CMT-93 through cross-talks of canonical Wnt signaling with VDR signaling pathway and/or LXR-related pathways. Consequently, we suggest that Wnt signaling-driven tumor formation and growth should be increased in mouse models involving 1638N-T1 compared to ones involving CMT-93. Though, many additional factors have to be taken into account when monitoring cell proliferation, invasion, and metastasis in mouse models. Several previous studies indicated synergistic effects between K-Ras and canonical Wnt signaling harboring APC mutations in CRC (Janssen et al., 2006; Luo et al., 2009; Lemieux et al., 2015). Furthermore, during development of effective cancer therapies, tumor cells grown *in vitro* are transplanted into ectopic sites of immunocompromized mice that do not reject tumor cells (Sharpless and Depinho, 2006; Richmond and Su, 2008; Hung et al., 2010). It has been stated that these xenograft models may fail to recapitulate the heterogeneity of cancer and the microenvironment, i.e., the interaction between tumor cells and supporting stroma (Hung et al., 2010). In the end, regardless of the fact that Wnt signaling may be aberrantly activated in 1638N-T1, a variety of different factors have an impact on the capacity of tumor cells to grow, proliferate, and metastasize in mouse models. We summarized our observations concerning the potential state of canonical Wnt signaling in the cell lines (Figure 5).

The master regulator analyses revealed several potential candidates which might be useful as therapeutic targets for cancer therapy. Master regulators were inferred from a network model that explicitly displayed the regulatory cascades between TFs. Beside several master regulators with yet unknown roles in CRC, we found MLK3 and Mapk1 (ERK2) which might be important in cancer cell proliferation, invasion, and metastasis of 1638N-T1 and CMT-93, respectively. Above all, our master regulatory networks can be used as models to generate testable hypotheses for studying the phenotypic differences between 1638N-T1 and CMT-93.

## 5. Conclusion

In this study, we have presented a systematic approach which combines colorectal cancer (CRC) cell lines, namely 1638N-T1 and CMT-93, and well-established computational methods in order to compare these cell lines on the level of transcriptional regulation as well as on a pathway level, i.e., the cancer cell-intrinsic pathway repertoire. We used the Trinity platform and the geneXplain platform to identify significantly upregulated genes in each of the cell lines as well as their upstream transcriptional regulators, on the basis of which we generated regulatory networks. Our findings suggested that the Wnt signaling pathway is activated in 1638N-T1, but inhibited in CMT-93 cells through cross-talks with other pathways. Moreover, we identified a number of well-known, cancer-associated TFs for both cell lines and provided indication of several master regulators being present such as MLK3 and Mapk1 (ERK2) which might be important in cell proliferation, migration, and invasion of 1638N-T1 and CMT-93, respectively. Using our systematic approach, we have provided new insights into the invasive potential of individual CRC cell lines, which can be used for development of effective cancer therapy.

## Author contributions

DW and MG participated in the design of the study, conducted computational, and statistical analyses. EW supervised the computational and statistical analyses. MH and TB interpreted the results with DW. JA prepared the colorectal cancer cell lines for this study. AW processed the RNA-Seq data (FASTQ files) and prepared the RNA-Seq count data. AB was involved in the preparation of the cell lines and interpretation of the results in perspective of colorectal cancer biology. DW and MG conceived of and managed the project and wrote the final version of the manuscript. All authors read and approved the final manuscript.

### Conflict of interest statement

The authors declare that the research was conducted in the absence of any commercial or financial relationships that could be construed as a potential conflict of interest.

## Supplementary material

Supplementary Table S1 **Signature genes for colorectal cell line 1638N-T1**.

Supplementary Table S2 **Signature genes for colorectal cell line CMT-93**.

Supplementary Table S3 **Colorectal cancer-related non-redundant PWM library**.

Supplementary Table S4 **1638N-T1-specific TF set**.

Supplementary Table S5 **CMT-93-specific TF set**.

Supplementary Table S6 **Intersection-specific TF set between 1638N-T1 and CMT-93**.

Supplementary Table S7 **Master regulatory network based on the intersection-specific TF set in Pair Graph File format**.

Supplementary Figure S7 **Master regulatory network based on the intersection-specific TF set as Scalable Vector Graphics (SVG)**.

Supplementary Table S8 **Master regulatory network based on the 1638NT-1-specific TF set in Pair Graph File format**.

Supplementary Figure S8 **Master regulatory network based on the 1638NT-1-specific TF set as Scalable Vector Graphics (SVG)**.

Supplementary Table S9 **Master regulatory network based on the CMT-93-specific TF set in Pair Graph File format**.

Supplementary Figure S9 **Master regulatory network based on the CMT-93-specific TF set as Scalable Vector Graphics (SVG)**.
