# Supplementary material for: Computational Identification of Key Regulators in Two Different Colorectal Cancer Cell Lines
Source: Front Genet. 2016 Apr 5;7:42. doi: 10.3389/fgene.2016.00042 (PMC4820448; doi:10.3389/fgene.2016.00042)
Supplement: Supplementary Table S1 — Signature genes for colorectal cell line 1638N-T1. [file Table1.PDF]

Table S1. The Trinity platform was first used to perform a differentially expressed gene (DEG) analysis based on RNA-seq data, which included three biological replicates for 1638N-T1 and CMT-93, respectively. In the second step, the genes were identified, which are most significantly upregulated in 1638N-T1 and, at the same time, downregulated in CMT-93. This category contains the signature genes for the cell line 1638N-T1.

ID	Gene symbol
ENSMUSG00000000093	Tbx2
ENSMUSG00000000142	Axin2
ENSMUSG00000000202	Btbd17
ENSMUSG00000000253	Gmpr
ENSMUSG00000000290	Itgb2
ENSMUSG00000000411	Tssk3
ENSMUSG00000000567	Sox9
ENSMUSG00000000791	Il12rb1
ENSMUSG00000000794	Kcnn3
ENSMUSG00000000861	Bcl11a
ENSMUSG00000000948	Snrpn
ENSMUSG00000001095	Slc13a2
ENSMUSG00000001119	Col6a1
ENSMUSG00000001120	Pcbp3
ENSMUSG00000001270	Ckb
ENSMUSG00000001493	Meox1
ENSMUSG00000001504	Irx2
ENSMUSG00000001506	Col1a1
ENSMUSG00000001555	Fkbp10
ENSMUSG00000001655	Hoxc13
ENSMUSG00000001663	Gstt1
ENSMUSG00000001665	Gstt3
ENSMUSG00000001666	Ddt
ENSMUSG00000001700	Gramd3
ENSMUSG00000001802	Lrp3
ENSMUSG00000001804	Dsg4
ENSMUSG00000001930	Vwf
ENSMUSG00000002020	Ltbp2
ENSMUSG00000002076	Hsf2bp
ENSMUSG00000002204	Napsa
ENSMUSG00000002265	Peg3
ENSMUSG00000002266	Zim1
ENSMUSG00000002688	Prkd1
ENSMUSG00000002900	Lamb1
ENSMUSG00000002944	Cd36
ENSMUSG00000003283	Hck
ENSMUSG00000003352	Cacnb3
ENSMUSG00000003420	Fcgrt
ENSMUSG00000003469	Phyhip
ENSMUSG00000003617	Cp
ENSMUSG00000004151	Etv1
ENSMUSG00000004231	Pax2
ENSMUSG00000004631	Sgce
ENSMUSG00000004633	Chn2
ENSMUSG00000004668	Abca13
ENSMUSG00000005089	Slc1a2
ENSMUSG00000005107	Slc2a9
ENSMUSG00000005320	Fgfr4
ENSMUSG00000005611	Mrvi1
ENSMUSG00000005686	Ampd3
ENSMUSG00000005800	Mmp8
ENSMUSG00000006235	Epor
ENSMUSG00000006369	Fbln1
ENSMUSG00000006403	Adamts4
ENSMUSG00000006435	Neurl1a
ENSMUSG00000006457	Actn3
ENSMUSG00000006576	Slc4a3
ENSMUSG00000006586	Runx1t1
ENSMUSG00000006651	Aplp1
ENSMUSG00000006800	Sulf2
ENSMUSG00000006931	Leprel4
ENSMUSG00000007107	Atp1a4
ENSMUSG00000007122	Casq1
ENSMUSG00000007908	Hmgcll1
ENSMUSG00000008489	Elavl2

ENSMUSG00000008540	Mgst1
ENSMUSG00000008734	Gprc5b
ENSMUSG00000008855	Hdac5
ENSMUSG00000008999	Bmp7
ENSMUSG00000009378	Slc16a12
ENSMUSG00000009621	Vav2
ENSMUSG00000010021	Kif19a
ENSMUSG00000010064	Slc38a3
ENSMUSG00000010066	Cacna2d2
ENSMUSG00000010175	Prox1
ENSMUSG00000010461	Eya4
ENSMUSG00000010651	Acaa1b
ENSMUSG00000011118	Panx3
ENSMUSG00000011256	Adam19
ENSMUSG00000011589	Fsd1
ENSMUSG00000012889	Podnl1
ENSMUSG00000013367	Iglon5
ENSMUSG00000013846	St3gal1
ENSMUSG00000014329	Bicc1
ENSMUSG00000014602	Kif1a
ENSMUSG00000014773	Dll1
ENSMUSG00000014813	Stc1
ENSMUSG00000015243	Abca1
ENSMUSG00000015340	Cybb
ENSMUSG00000015579	Nkx2-5
ENSMUSG00000015619	Gata3
ENSMUSG00000015709	Arnt2
ENSMUSG00000015839	Nfe2l2
ENSMUSG00000015890	Amdhd1
ENSMUSG00000015957	Wnt11
ENSMUSG00000016024	Lbp
ENSMUSG00000016255	Tubb1
ENSMUSG00000016349	Eef1a2
ENSMUSG00000016386	Mpped2
ENSMUSG00000016458	Wt1
ENSMUSG00000016624	Phf21b
ENSMUSG00000017417	Plxdc1
ENSMUSG00000017446	C1qtnf1
ENSMUSG00000017491	Rarb
ENSMUSG00000017607	Tns4
ENSMUSG00000017688	Hnf4g
ENSMUSG00000017692	Rhbdl3
ENSMUSG00000017713	Tha1
ENSMUSG00000017737	Mmp9
ENSMUSG00000017740	Slc12a5
ENSMUSG00000017754	Pltp
ENSMUSG00000017978	Cadps2
ENSMUSG00000018008	Cyth4
ENSMUSG00000018340	Anxa6
ENSMUSG00000018387	Shroom1
ENSMUSG00000018554	Ybx2
ENSMUSG00000018623	Mmp7
ENSMUSG00000018819	Lsp1
ENSMUSG00000018861	Fdxr
ENSMUSG00000018916	Csf2
ENSMUSG00000019122	Ccl9
ENSMUSG00000019194	Scn1b
ENSMUSG00000019256	Ahr
ENSMUSG00000019647	Sema6a
ENSMUSG00000019775	Rgs17
ENSMUSG00000019836	Amd-ps4
ENSMUSG00000019838	Slc16a10
ENSMUSG00000019846	Lama4
ENSMUSG00000019856	Fam184a
ENSMUSG00000019872	Smpdl3a
ENSMUSG00000019894	Slc6a15
ENSMUSG00000019929	Dcn
ENSMUSG00000019997	Ctgf
ENSMUSG00000020009	Ifngr1
ENSMUSG00000020010	Vnn3
ENSMUSG00000020057	Dram1
ENSMUSG00000020062	Slc5a8

ENSMUSG00000020081	Tacr2
ENSMUSG00000020083	2010107G23Rik
ENSMUSG00000020086	H2afy2
ENSMUSG00000020099	Unc5b
ENSMUSG00000020101	4632428N05Rik
ENSMUSG00000020102	Slc16a7
ENSMUSG00000020105	Lrig3
ENSMUSG00000020108	Ddit4
ENSMUSG00000020123	Avpr1a
ENSMUSG00000020140	Lgr5
ENSMUSG00000020159	Gabrp
ENSMUSG00000020164	1700058G18Rik
ENSMUSG00000020176	Grb10
ENSMUSG00000020178	Adora2a
ENSMUSG00000020241	Col6a2
ENSMUSG00000020310	Madcam1
ENSMUSG00000020312	Shc2
ENSMUSG00000020326	Ccng1
ENSMUSG00000020331	Hcn2
ENSMUSG00000020340	Cyfip2
ENSMUSG00000020357	Flt4
ENSMUSG00000020363	Gfpt2
ENSMUSG00000020364	Zfp354a
ENSMUSG00000020396	Nefh
ENSMUSG00000020411	Nipal4
ENSMUSG00000020431	Adcy1
ENSMUSG00000020600	Slc7a15
ENSMUSG00000020601	Trib2
ENSMUSG00000020614	Fam20a
ENSMUSG00000020620	Abca8b
ENSMUSG00000020627	Klhl29
ENSMUSG00000020644	Id2
ENSMUSG00000020810	Cygb
ENSMUSG00000020838	Slc6a4
ENSMUSG00000020865	Abcc3
ENSMUSG00000020866	Cacna1g
ENSMUSG00000020870	Cdc34-ps
ENSMUSG00000020886	Dlg4
ENSMUSG00000020901	Pik3r5
ENSMUSG00000020902	Ntn1
ENSMUSG00000020941	Map3k14
ENSMUSG00000020950	Foxg1
ENSMUSG00000020953	Coch
ENSMUSG00000021047	Nova1
ENSMUSG00000021069	Pygl
ENSMUSG00000021071	Trim9
ENSMUSG00000021127	Zfp36l1
ENSMUSG00000021130	Galnt16
ENSMUSG00000021185	9030617O03Rik
ENSMUSG00000021200	Asb2
ENSMUSG00000021217	Tshz3
ENSMUSG00000021236	Entpd5
ENSMUSG00000021256	Vash1
ENSMUSG00000021259	Cyp46a1
ENSMUSG00000021260	Hhipl1
ENSMUSG00000021280	Exoc3l4
ENSMUSG00000021303	Gng4
ENSMUSG00000021318	Gli3
ENSMUSG00000021356	Irf4
ENSMUSG00000021379	Id4
ENSMUSG00000021457	Syk
ENSMUSG00000021469	Msx2
ENSMUSG00000021488	Nsd1
ENSMUSG00000021559	Dapk1
ENSMUSG00000021565	Slc6a19
ENSMUSG00000021591	Glrx
ENSMUSG00000021612	Slc6a18
ENSMUSG00000021676	Iqgap2
ENSMUSG00000021720	Rnf180
ENSMUSG00000021725	Parp8
ENSMUSG00000021747	4930452B06Rik
ENSMUSG00000021750	Fam107a

ENSMUSG00000021779	Thrb
ENSMUSG00000021795	Sftpd
ENSMUSG00000021803	Cdhr1
ENSMUSG00000021850	1700011H14Rik
ENSMUSG00000021986	Amer2
ENSMUSG00000021994	Wnt5a
ENSMUSG00000022032	Scara5
ENSMUSG00000022044	Stmn4
ENSMUSG00000022054	Nefm
ENSMUSG00000022055	Nefl
ENSMUSG00000022123	Scel
ENSMUSG00000022148	Fyb
ENSMUSG00000022150	Dab2
ENSMUSG00000022156	Gzme
ENSMUSG00000022157	Mcpt8
ENSMUSG00000022180	Slc7a8
ENSMUSG00000022206	Npr3
ENSMUSG00000022211	Lrrc16b
ENSMUSG00000022229	Atp12a
ENSMUSG00000022257	Laptm4b
ENSMUSG00000022304	Dpys
ENSMUSG00000022324	Matn2
ENSMUSG00000022330	Osr2
ENSMUSG00000022367	Has2
ENSMUSG00000022415	Syngr1
ENSMUSG00000022425	Enpp2
ENSMUSG00000022429	Dmc1
ENSMUSG00000022440	C1qtnf6
ENSMUSG00000022456	Sept3
ENSMUSG00000022474	Pmm1
ENSMUSG00000022512	Cldn1
ENSMUSG00000022534	Mefv
ENSMUSG00000022537	Tmem44
ENSMUSG00000022583	Ly6f
ENSMUSG00000022623	Shank3
ENSMUSG00000022636	Alcam
ENSMUSG00000022639	5330426P16Rik
ENSMUSG00000022665	Ccdc80
ENSMUSG00000022705	Drd3
ENSMUSG00000022755	Gpr128
ENSMUSG00000022756	Slc7a4
ENSMUSG00000022758	P2rx6
ENSMUSG00000022836	Mylk
ENSMUSG00000022871	Fetub
ENSMUSG00000022883	Robo1
ENSMUSG00000022947	Cbr3
ENSMUSG00000022949	Clic6
ENSMUSG00000022996	Wnt10b
ENSMUSG00000022997	Wnt1
ENSMUSG00000023009	Nckap5l
ENSMUSG00000023031	Cela1
ENSMUSG00000023032	Slc4a8
ENSMUSG00000023067	Cdkn1a
ENSMUSG00000023070	Rgn
ENSMUSG00000023092	Fhl1
ENSMUSG00000023094	Msrb2
ENSMUSG00000023122	Sult1c2
ENSMUSG00000023191	Leprel2
ENSMUSG00000023206	Il15ra
ENSMUSG00000023247	Guca2a
ENSMUSG00000023484	Prph
ENSMUSG00000023913	Pla2g7
ENSMUSG00000023927	Satb1
ENSMUSG00000023947	Nfkbie
ENSMUSG00000024032	Tff1
ENSMUSG00000024033	Rsph1
ENSMUSG00000024036	Slc37a1
ENSMUSG00000024059	Clip4
ENSMUSG00000024064	Galnt14
ENSMUSG00000024065	Ehd3
ENSMUSG00000024066	Xdh
ENSMUSG00000024084	Qpct

ENSMUSG00000024087	Cyp1b1
ENSMUSG00000024112	Cacna1h
ENSMUSG00000024134	Six2
ENSMUSG00000024140	Epas1
ENSMUSG00000024164	C3
ENSMUSG00000024176	Sox8
ENSMUSG00000024205	Rpl36-ps2
ENSMUSG00000024222	Fkbp5
ENSMUSG00000024232	Bambi
ENSMUSG00000024247	Pkdcc
ENSMUSG00000024299	Adamts10
ENSMUSG00000024304	Cdh2
ENSMUSG00000024330	Col11a2
ENSMUSG00000024331	Dsc2
ENSMUSG00000024386	Proc
ENSMUSG00000024402	Lta
ENSMUSG00000024420	Zfp521
ENSMUSG00000024427	Spry4
ENSMUSG00000024479	Mal2
ENSMUSG00000024501	Dpysl3
ENSMUSG00000024502	Jakmip2
ENSMUSG00000024529	Lox
ENSMUSG00000024534	Sncaip
ENSMUSG00000024538	Ppic
ENSMUSG00000024548	Setbp1
ENSMUSG00000024598	Fbn2
ENSMUSG00000024669	Cd5
ENSMUSG00000024677	Ms4a6b
ENSMUSG00000024678	Ms4a4d
ENSMUSG00000024691	Fam111a
ENSMUSG00000024713	Pcsk5
ENSMUSG00000024736	Tmem132a
ENSMUSG00000024737	Slc15a3
ENSMUSG00000024743	Syt7
ENSMUSG00000024778	Fas
ENSMUSG00000024867	Pip5k1b
ENSMUSG00000024897	Apba1
ENSMUSG00000024899	Papss2
ENSMUSG00000024986	Hhex
ENSMUSG00000025075	Habp2
ENSMUSG00000025082	Vwa2
ENSMUSG00000025089	Gfra1
ENSMUSG00000025104	Hdgfrp3
ENSMUSG00000025176	Hoga1
ENSMUSG00000025196	Cpn1
ENSMUSG00000025216	Lbx1
ENSMUSG00000025255	Zfhx4
ENSMUSG00000025272	Tro
ENSMUSG00000025324	Atp10a
ENSMUSG00000025352	Gdf11
ENSMUSG00000025407	Gli1
ENSMUSG00000025431	Crisp1
ENSMUSG00000025433	Crisp3
ENSMUSG00000025529	Zfp711
ENSMUSG00000025586	Cpeb1
ENSMUSG00000025610	Map3k7cl
ENSMUSG00000025735	Rhbdl1
ENSMUSG00000025743	Sdc3
ENSMUSG00000025784	Clec3b
ENSMUSG00000025867	Cplx2
ENSMUSG00000025887	Casp12
ENSMUSG00000025915	Sgk3
ENSMUSG00000025922	Gm5045
ENSMUSG00000025934	Gsta3
ENSMUSG00000025964	Adam23
ENSMUSG00000025986	Slc39a10
ENSMUSG00000025991	Cps1
ENSMUSG00000025993	Slc40a1
ENSMUSG00000026009	Icos
ENSMUSG00000026012	Cd28
ENSMUSG00000026072	Il1r1
ENSMUSG00000026077	Npas2

ENSMUSG00000026109	Tmeff2
ENSMUSG00000026117	Zap70
ENSMUSG00000026149	Tm4sf20
ENSMUSG00000026166	Ccl20
ENSMUSG00000026185	Igfbp5
ENSMUSG00000026188	Tmem169
ENSMUSG00000026207	Speg
ENSMUSG00000026208	Des
ENSMUSG00000026249	Serpine2
ENSMUSG00000026285	Pdcd1
ENSMUSG00000026303	Mlph
ENSMUSG00000026308	Klhl30
ENSMUSG00000026360	Rgs2
ENSMUSG00000026365	Cfh
ENSMUSG00000026389	Steap3
ENSMUSG00000026398	Nr5a2
ENSMUSG00000026405	C4bp
ENSMUSG00000026452	Syt2
ENSMUSG00000026468	Lhx4
ENSMUSG00000026473	Glul
ENSMUSG00000026527	Rgs7
ENSMUSG00000026579	F5
ENSMUSG00000026610	Esrrg
ENSMUSG00000026620	Mark1
ENSMUSG00000026630	Batf3
ENSMUSG00000026637	Traf5
ENSMUSG00000026676	Ccdc3
ENSMUSG00000026700	Tnfsf4
ENSMUSG00000026736	4930426L09Rik
ENSMUSG00000026737	Pip4k2a
ENSMUSG00000026811	St6galnac6
ENSMUSG00000026817	Ak1
ENSMUSG00000026837	Col5a1
ENSMUSG00000026875	Traf1
ENSMUSG00000026930	Gpsm1
ENSMUSG00000026981	Il1rn
ENSMUSG00000027188	Pamr1
ENSMUSG00000027200	Sema6d
ENSMUSG00000027201	Myef2
ENSMUSG00000027202	Slc12a1
ENSMUSG00000027227	Sord
ENSMUSG00000027254	Map1a
ENSMUSG00000027261	Hao1
ENSMUSG00000027358	Bmp2
ENSMUSG00000027371	Fahd2a
ENSMUSG00000027460	Angpt4
ENSMUSG00000027474	Ccm2l
ENSMUSG00000027488	Snta1
ENSMUSG00000027514	Zbp1
ENSMUSG00000027555	Car13
ENSMUSG00000027603	Ggt7
ENSMUSG00000027692	Tnik
ENSMUSG00000027750	Postn
ENSMUSG00000027765	P2ry1
ENSMUSG00000027797	Dclk1
ENSMUSG00000027800	Tm4sf1
ENSMUSG00000027820	Mme
ENSMUSG00000027831	Veph1
ENSMUSG00000027832	Ptx3
ENSMUSG00000027848	Olfml3
ENSMUSG00000027894	Slc6a17
ENSMUSG00000027895	Kcnc4
ENSMUSG00000027925	Sprr2j-ps
ENSMUSG00000027931	Npr1
ENSMUSG00000027955	Fam198b
ENSMUSG00000027956	Tmem144
ENSMUSG00000027962	Vcam1
ENSMUSG00000027985	Lef1
ENSMUSG00000027993	Trim2
ENSMUSG00000027995	Tlr2
ENSMUSG00000027996	Sfrp2
ENSMUSG00000028005	Gucy1b3

ENSMUSG00000028011	Tdo2
ENSMUSG00000028015	Ctso
ENSMUSG00000028020	Glrb
ENSMUSG00000028023	Pitx2
ENSMUSG00000028033	Kcnq5
ENSMUSG00000028036	Ptgfr
ENSMUSG00000028108	Ecm1
ENSMUSG00000028184	Lphn2
ENSMUSG00000028211	Trp53inp1
ENSMUSG00000028238	Atp6v0d2
ENSMUSG00000028262	Clca2
ENSMUSG00000028278	Rragd
ENSMUSG00000028289	Epha7
ENSMUSG00000028354	Fmn2
ENSMUSG00000028358	Zfp618
ENSMUSG00000028359	Orm3
ENSMUSG00000028370	Pappa
ENSMUSG00000028389	Zfp37
ENSMUSG00000028399	Ptprd
ENSMUSG00000028402	Mpdz
ENSMUSG00000028415	Spink4
ENSMUSG00000028476	Reck
ENSMUSG00000028517	Ppap2b
ENSMUSG00000028518	Prkaa2
ENSMUSG00000028565	Nfia
ENSMUSG00000028600	Podn
ENSMUSG00000028631	Kcnq4
ENSMUSG00000028713	Cyp4b1
ENSMUSG00000028766	Alpl
ENSMUSG00000028782	Bai2
ENSMUSG00000028834	Trim63
ENSMUSG00000028838	Extl1
ENSMUSG00000028909	Ptpru
ENSMUSG00000028919	Arhgef19
ENSMUSG00000028926	Cdk14
ENSMUSG00000028943	Espn
ENSMUSG00000029070	Mxra8
ENSMUSG00000029093	Sorcs2
ENSMUSG00000029096	Htra3
ENSMUSG00000029108	Pcdh7
ENSMUSG00000029126	Nsg1
ENSMUSG00000029153	Ociad2
ENSMUSG00000029168	Dpysl5
ENSMUSG00000029206	Nsun7
ENSMUSG00000029260	Ugt2b34
ENSMUSG00000029304	Spp1
ENSMUSG00000029348	Asphd2
ENSMUSG00000029359	Tesc
ENSMUSG00000029372	Ppbp
ENSMUSG00000029375	Il8
ENSMUSG00000029380	Cxcl1
ENSMUSG00000029419	Gm996
ENSMUSG00000029431	B3gnt4
ENSMUSG00000029436	Mmp17
ENSMUSG00000029455	Aldh2
ENSMUSG00000029546	Uncx
ENSMUSG00000029563	Foxp2
ENSMUSG00000029595	Lhx5
ENSMUSG00000029633	Gm5578
ENSMUSG00000029641	Rasl11a
ENSMUSG00000029644	Pdx1
ENSMUSG00000029646	Cdx2
ENSMUSG00000029648	Flt1
ENSMUSG00000029673	Auts2
ENSMUSG00000029718	Pcolce
ENSMUSG00000029755	Dlx5
ENSMUSG00000029765	Plxna4
ENSMUSG00000029771	Irf5
ENSMUSG00000029811	Abp1
ENSMUSG00000029851	Fam115c
ENSMUSG00000029875	AI836003
ENSMUSG00000029913	Prdm5

ENSMUSG00000030022	Adamts9
ENSMUSG00000030087	Klf15
ENSMUSG00000030123	Plxnd1
ENSMUSG00000030157	Clec2d
ENSMUSG00000030207	8430419L09Rik
ENSMUSG00000030209	Grin2b
ENSMUSG00000030237	Slco1a4
ENSMUSG00000030246	Ldhb
ENSMUSG00000030247	Kcnj8
ENSMUSG00000030263	Lrmp
ENSMUSG00000030268	Bcat1
ENSMUSG00000030283	St8sia1
ENSMUSG00000030306	Tmtc1
ENSMUSG00000030499	Kctd15
ENSMUSG00000030519	Apba2
ENSMUSG00000030554	Synm
ENSMUSG00000030555	Ttc23
ENSMUSG00000030559	Rab38
ENSMUSG00000030560	Ctsc
ENSMUSG00000030600	Lrfn1
ENSMUSG00000030653	Pde2a
ENSMUSG00000030688	Stard10
ENSMUSG00000030703	Gdpd3
ENSMUSG00000030732	Chrdl2
ENSMUSG00000030768	Disp1
ENSMUSG00000030769	Slc5a11
ENSMUSG00000030786	Itgam
ENSMUSG00000030849	Fgfr2
ENSMUSG00000030862	Cpxm2
ENSMUSG00000030917	Tmem159
ENSMUSG00000030946	Lhpp
ENSMUSG00000030956	Fam53b
ENSMUSG00000030972	Acsm5
ENSMUSG00000031023	Akip1
ENSMUSG00000031028	Tub
ENSMUSG00000031097	Tnni2
ENSMUSG00000031098	Syt8
ENSMUSG00000031112	2610018G03Rik
ENSMUSG00000031119	Gpc4
ENSMUSG00000031137	Fgf13
ENSMUSG00000031216	Stard8
ENSMUSG00000031233	Pgk2
ENSMUSG00000031271	Serpina7
ENSMUSG00000031274	Col4a5
ENSMUSG00000031283	Chrdl1
ENSMUSG00000031289	Il13ra2
ENSMUSG00000031290	Lrch2
ENSMUSG00000031303	Map3k15
ENSMUSG00000031327	Chic1
ENSMUSG00000031340	Gabre
ENSMUSG00000031342	Gpm6b
ENSMUSG00000031355	Arhgap6
ENSMUSG00000031380	Figf
ENSMUSG00000031430	Vsig1
ENSMUSG00000031438	Rnf128
ENSMUSG00000031486	Gpr124
ENSMUSG00000031504	Rab20
ENSMUSG00000031558	Slit2
ENSMUSG00000031561	Tenm3
ENSMUSG00000031565	Fgfr1
ENSMUSG00000031616	Ednra
ENSMUSG00000031626	Sorbs2
ENSMUSG00000031665	Sall1
ENSMUSG00000031734	Irx3
ENSMUSG00000031767	Nudt7
ENSMUSG00000031825	Crispld2
ENSMUSG00000031842	Pde4c
ENSMUSG00000031853	BC021891
ENSMUSG00000031870	Pgr
ENSMUSG00000031877	Ces2g
ENSMUSG00000031881	Cdh16
ENSMUSG00000031886	Ces2e

ENSMUSG00000031906	Smpd3
ENSMUSG00000031962	Cdh15
ENSMUSG00000031963	Bmper
ENSMUSG00000031972	Acta1
ENSMUSG00000032000	Birc3
ENSMUSG00000032006	Pdgfd
ENSMUSG00000032012	Pvrl1
ENSMUSG00000032014	Oaf
ENSMUSG00000032015	Pou2f3
ENSMUSG00000032033	Barx2
ENSMUSG00000032128	Robo3
ENSMUSG00000032186	Tmod2
ENSMUSG00000032204	Aqp9
ENSMUSG00000032271	Nnmt
ENSMUSG00000032289	Thsd4
ENSMUSG00000032322	Pstpip1
ENSMUSG00000032327	Stra6
ENSMUSG00000032332	Col12a1
ENSMUSG00000032344	Mb21d1
ENSMUSG00000032348	Gsta4
ENSMUSG00000032350	Gclc
ENSMUSG00000032357	Tinag
ENSMUSG00000032387	Rbpms2
ENSMUSG00000032401	Lctl
ENSMUSG00000032487	Ptgs2
ENSMUSG00000032492	Pth1r
ENSMUSG00000032511	Scn5a
ENSMUSG00000032554	Trf
ENSMUSG00000032564	Cpne4
ENSMUSG00000032718	Mansc1
ENSMUSG00000032735	Ablim3
ENSMUSG00000032816	Igdcc4
ENSMUSG00000032841	Prr5l
ENSMUSG00000032892	Rangrf
ENSMUSG00000033053	1700028P14Rik
ENSMUSG00000033174	Mgll
ENSMUSG00000033214	Slitrk5
ENSMUSG00000033256	Shf
ENSMUSG00000033278	Ptprm
ENSMUSG00000033318	Gstt2
ENSMUSG00000033350	Chst2
ENSMUSG00000033355	Rtp4
ENSMUSG00000033420	Antxr1
ENSMUSG00000033453	Adamts15
ENSMUSG00000033533	Acsm1
ENSMUSG00000033715	Akr1c14
ENSMUSG00000033788	Dysf
ENSMUSG00000033854	Kcnk10
ENSMUSG00000033857	Engase
ENSMUSG00000033871	Ppargc1b
ENSMUSG00000033880	Lgals3bp
ENSMUSG00000034161	Scx
ENSMUSG00000034227	Foxj1
ENSMUSG00000034275	Igsf9b
ENSMUSG00000034308	Sdr42e1
ENSMUSG00000034330	Plcg2
ENSMUSG00000034336	Ina
ENSMUSG00000034422	Parp14
ENSMUSG00000034452	Slc24a1
ENSMUSG00000034456	Uroc1
ENSMUSG00000034457	Eda2r
ENSMUSG00000034463	Scara3
ENSMUSG00000034488	Edil3
ENSMUSG00000034551	Hdx
ENSMUSG00000034570	Inpp5j
ENSMUSG00000034579	Pla2g3
ENSMUSG00000034591	Slc41a2
ENSMUSG00000034613	Ppm1h
ENSMUSG00000034652	Cd300a
ENSMUSG00000034684	Sema3f
ENSMUSG00000034685	Fam171a2
ENSMUSG00000034706	Dnaic2

ENSMUSG00000034714	Ttyh2
ENSMUSG00000034758	Tle6
ENSMUSG00000034771	Tle2
ENSMUSG00000034818	Celf5
ENSMUSG00000034845	Plvap
ENSMUSG00000034853	Acot11
ENSMUSG00000034910	Pygo1
ENSMUSG00000034936	Arl4d
ENSMUSG00000034959	5031414D18Rik
ENSMUSG00000035045	Zc3h12b
ENSMUSG00000035067	Xkr6
ENSMUSG00000035104	Eva1a
ENSMUSG00000035105	Egln3
ENSMUSG00000035158	Mitf
ENSMUSG00000035183	Slc24a5
ENSMUSG00000035184	Fam124a
ENSMUSG00000035226	Rims4
ENSMUSG00000035258	Abi3bp
ENSMUSG00000035262	Amh
ENSMUSG00000035277	Arx
ENSMUSG00000035279	Ssc5d
ENSMUSG00000035385	Ccl2
ENSMUSG00000035392	Dennd1a
ENSMUSG00000035403	Crb2
ENSMUSG00000035513	Ntng2
ENSMUSG00000035540	Gc
ENSMUSG00000035561	Aldh1b1
ENSMUSG00000035566	Pcdh17
ENSMUSG00000035592	Krt33a
ENSMUSG00000035735	Dagla
ENSMUSG00000035799	Twist1
ENSMUSG00000035811	Ugt2b35
ENSMUSG00000035829	Ppp1r26
ENSMUSG00000035929	H2-Q4
ENSMUSG00000035934	Pknox2
ENSMUSG00000035948	Acss3
ENSMUSG00000036030	Prtg
ENSMUSG00000036098	Myrf
ENSMUSG00000036225	Kctd1
ENSMUSG00000036249	Rbm43
ENSMUSG00000036264	Fstl4
ENSMUSG00000036356	Csgalnact1
ENSMUSG00000036463	4930544G11Rik
ENSMUSG00000036528	Ppfibp2
ENSMUSG00000036545	Adamts2
ENSMUSG00000036585	Fgf1
ENSMUSG00000036602	Alx1
ENSMUSG00000036699	Zcchc12
ENSMUSG00000036782	Klhl13
ENSMUSG00000036867	Smad6
ENSMUSG00000036902	Neto2
ENSMUSG00000036904	Fzd8
ENSMUSG00000036912	Piwil4
ENSMUSG00000036957	Lrfn3
ENSMUSG00000036995	Asap3
ENSMUSG00000037138	Aff3
ENSMUSG00000037139	Myom3
ENSMUSG00000037157	Il22ra1
ENSMUSG00000037211	Spry1
ENSMUSG00000037239	Spred3
ENSMUSG00000037254	Itih2
ENSMUSG00000037306	Man1c1
ENSMUSG00000037321	Tap1
ENSMUSG00000037341	Slc9a7
ENSMUSG00000037347	Chst7
ENSMUSG00000037362	Nov
ENSMUSG00000037370	Enpp1
ENSMUSG00000037379	Spon2
ENSMUSG00000037411	Serpine1
ENSMUSG00000037438	Gm9763
ENSMUSG00000037579	Kcnh3
ENSMUSG00000037605	Lphn3

ENSMUSG00000037621	Atoh8
ENSMUSG00000037709	Fam13a
ENSMUSG00000037762	Slc16a9
ENSMUSG00000037784	Dzip1l
ENSMUSG00000037820	Tgm2
ENSMUSG00000037902	Sirpa
ENSMUSG00000037942	Crp
ENSMUSG00000037944	Ccr7
ENSMUSG00000037962	Fam101a
ENSMUSG00000038020	Rapgefl1
ENSMUSG00000038058	Nod1
ENSMUSG00000038128	Camk4
ENSMUSG00000038143	Stox2
ENSMUSG00000038146	Notch3
ENSMUSG00000038151	Prdm1
ENSMUSG00000038178	Slc43a2
ENSMUSG00000038246	Fam50b
ENSMUSG00000038248	Sobp
ENSMUSG00000038305	Spats2l
ENSMUSG00000038311	2410017I17Rik
ENSMUSG00000038319	Kcnh2
ENSMUSG00000038370	Pcp4l1
ENSMUSG00000038390	Gpr162
ENSMUSG00000038408	1700018A04Rik
ENSMUSG00000038456	Dennd2a
ENSMUSG00000038463	Olfml2b
ENSMUSG00000038473	Nos1ap
ENSMUSG00000038508	Gdf15
ENSMUSG00000038517	Tbkbp1
ENSMUSG00000038522	AI317395
ENSMUSG00000038587	Akap12
ENSMUSG00000038630	Zkscan16
ENSMUSG00000038668	Lpar1
ENSMUSG00000038677	Scube3
ENSMUSG00000038679	Trps1
ENSMUSG00000038738	Shank1
ENSMUSG00000038765	Lmx1b
ENSMUSG00000038768	9130409I23Rik
ENSMUSG00000038776	Ephx1
ENSMUSG00000038777	Sema6c
ENSMUSG00000038879	Nipal2
ENSMUSG00000038916	Soga3
ENSMUSG00000039081	Zfp503
ENSMUSG00000039095	En2
ENSMUSG00000039115	Itga9
ENSMUSG00000039133	9330171B17Rik
ENSMUSG00000039137	Whrn
ENSMUSG00000039209	Rpl39l
ENSMUSG00000039224	D1Pas1
ENSMUSG00000039238	Zfp750
ENSMUSG00000039239	Tgfb2
ENSMUSG00000039323	Igfbp2
ENSMUSG00000039328	Rnf122
ENSMUSG00000039385	Cdh6
ENSMUSG00000039450	Dcxr
ENSMUSG00000039542	Ncam1
ENSMUSG00000039601	Rcan2
ENSMUSG00000039611	Tmem246
ENSMUSG00000039620	6430573F11Rik
ENSMUSG00000039632	Ccdc151
ENSMUSG00000039646	Vasn
ENSMUSG00000039676	Capsl
ENSMUSG00000039716	Dock3
ENSMUSG00000039942	Ptger4
ENSMUSG00000039954	Stk32a
ENSMUSG00000039997	Ifi203
ENSMUSG00000040009	Gnaz
ENSMUSG00000040016	Ptger3
ENSMUSG00000040093	Bmf
ENSMUSG00000040118	Cacna2d1
ENSMUSG00000040133	Gpr176
ENSMUSG00000040140	Tdrd6

ENSMUSG00000040146	Rgl3
ENSMUSG00000040183	Ankrd6
ENSMUSG00000040219	Ttc12
ENSMUSG00000040258	Nxph4
ENSMUSG00000040265	Dnm3
ENSMUSG00000040289	Hey1
ENSMUSG00000040328	Olfr56
ENSMUSG00000040350	Trim7
ENSMUSG00000040405	Havcr1
ENSMUSG00000040413	Timd2
ENSMUSG00000040447	Spns2
ENSMUSG00000040594	Ranbp17
ENSMUSG00000040605	Bace2
ENSMUSG00000040606	Kazn
ENSMUSG00000040613	Apobec1
ENSMUSG00000040616	Tmem51
ENSMUSG00000040649	Rimklb
ENSMUSG00000040690	Col16a1
ENSMUSG00000040710	St8sia4
ENSMUSG00000040713	Creg1
ENSMUSG00000040808	S100g
ENSMUSG00000040852	Plekhh2
ENSMUSG00000040875	Osbpl10
ENSMUSG00000040957	Cables1
ENSMUSG00000040964	Arhgef10l
ENSMUSG00000041000	Trim62
ENSMUSG00000041020	Map7d2
ENSMUSG00000041078	Grid1
ENSMUSG00000041144	Dnahc7b
ENSMUSG00000041180	Hectd2
ENSMUSG00000041237	Pklr
ENSMUSG00000041261	Car8
ENSMUSG00000041272	Tox
ENSMUSG00000041351	Rap1gap
ENSMUSG00000041390	Mdfic
ENSMUSG00000041477	Dcp1b
ENSMUSG00000041482	Piezo2
ENSMUSG00000041515	Irf8
ENSMUSG00000041540	Sox5
ENSMUSG00000041548	Hspb8
ENSMUSG00000041698	Slco1a1
ENSMUSG00000041729	Coro2b
ENSMUSG00000041731	Pgm5
ENSMUSG00000041741	Pde3a
ENSMUSG00000041797	Abca9
ENSMUSG00000041801	Phlda3
ENSMUSG00000041828	Abca8a
ENSMUSG00000041911	Dlx1
ENSMUSG00000041930	Fam222a
ENSMUSG00000042182	Bend6
ENSMUSG00000042246	Tmc7
ENSMUSG00000042258	Isl1
ENSMUSG00000042268	Slc26a9
ENSMUSG00000042284	Itga1
ENSMUSG00000042286	Stab1
ENSMUSG00000042293	Gm5617
ENSMUSG00000042359	Osbpl6
ENSMUSG00000042388	Dlgap3
ENSMUSG00000042396	Rbm7
ENSMUSG00000042476	Abcb4
ENSMUSG00000042498	D330045A20Rik
ENSMUSG00000042500	Ago4
ENSMUSG00000042532	Golga7b
ENSMUSG00000042686	Jph1
ENSMUSG00000042763	Maneal
ENSMUSG00000042812	Foxf1
ENSMUSG00000042834	Nrep
ENSMUSG00000042918	Mamstr
ENSMUSG00000042961	Egflam
ENSMUSG00000042985	Upk3b
ENSMUSG00000042988	Notum
ENSMUSG00000043013	Onecut1

ENSMUSG00000043088	Il17re
ENSMUSG00000043223	Gm4835
ENSMUSG00000043289	Mei4
ENSMUSG00000043415	Otud1
ENSMUSG00000043439	E130012A19Rik
ENSMUSG00000043496	Tril
ENSMUSG00000043518	Rai2
ENSMUSG00000043531	Sorcs1
ENSMUSG00000043557	Mdga1
ENSMUSG00000043587	Acpl2
ENSMUSG00000043613	Mmp3
ENSMUSG00000043664	Tmem221
ENSMUSG00000043687	1190005I06Rik
ENSMUSG00000043795	Gm14492
ENSMUSG00000043843	Tmem145
ENSMUSG00000043903	Gm22
ENSMUSG00000043953	Ccrl2
ENSMUSG00000043969	Emx2
ENSMUSG00000044017	Gpr133
ENSMUSG00000044033	Ccdc141
ENSMUSG00000044043	Pcdhb14
ENSMUSG00000044065	Gm9788
ENSMUSG00000044068	Zrsr1
ENSMUSG00000044162	Tnip3
ENSMUSG00000044211	Gm7887
ENSMUSG00000044243	Bhlha9
ENSMUSG00000044313	Mab21l3
ENSMUSG00000044322	Dsc1
ENSMUSG00000044352	Sowaha
ENSMUSG00000044365	Cxxc4
ENSMUSG00000044434	Gm9791
ENSMUSG00000044562	Rasip1
ENSMUSG00000044576	Gareml
ENSMUSG00000044609	Gm9294
ENSMUSG00000044646	Zbtb7c
ENSMUSG00000044674	Fzd1
ENSMUSG00000044694	2010007H06Rik
ENSMUSG00000044749	Abca6
ENSMUSG00000044807	Zfp354c
ENSMUSG00000044827	Tlr1
ENSMUSG00000044921	Rassf9
ENSMUSG00000045062	Pcdhb7
ENSMUSG00000045075	Gm9796
ENSMUSG00000045087	S1pr5
ENSMUSG00000045103	Dmd
ENSMUSG00000045104	Gm5514
ENSMUSG00000045287	Rtn4rl1
ENSMUSG00000045318	Adra2c
ENSMUSG00000045382	Cxcr4
ENSMUSG00000045498	Pcdhb3
ENSMUSG00000045515	Pou3f3
ENSMUSG00000045657	Pcdhb10
ENSMUSG00000045664	Cdc42ep2
ENSMUSG00000045672	Col27a1
ENSMUSG00000045689	Pcdhb4
ENSMUSG00000045725	Prr15
ENSMUSG00000045730	Adrb2
ENSMUSG00000045761	Fam179a
ENSMUSG00000045790	Ccdc149
ENSMUSG00000045842	Gm7148
ENSMUSG00000045868	Gvin1
ENSMUSG00000045876	Pcdhb8
ENSMUSG00000045912	C2cd4c
ENSMUSG00000045928	4933440M02Rik
ENSMUSG00000045954	Sdpr
ENSMUSG00000045991	Onecut2
ENSMUSG00000046049	Rp1l1
ENSMUSG00000046085	4931422A03Rik
ENSMUSG00000046157	Tmem229b
ENSMUSG00000046173	Pabpc6
ENSMUSG00000046203	Sprr2g
ENSMUSG00000046207	Pik3r6

ENSMUSG00000046275	Tusc5
ENSMUSG00000046318	Ccbe1
ENSMUSG00000046413	D230002A01Rik
ENSMUSG00000046449	C77370
ENSMUSG00000046550	Spin2
ENSMUSG00000046598	Bdh1
ENSMUSG00000046620	Rps11-ps4
ENSMUSG00000046668	Cxxc5
ENSMUSG00000046687	Gm5424
ENSMUSG00000046714	Foxc2
ENSMUSG00000046743	Fat4
ENSMUSG00000046807	Fam211b
ENSMUSG00000046818	Ddit4l
ENSMUSG00000046841	Ckap4
ENSMUSG00000046879	Irgm1
ENSMUSG00000046934	Csl
ENSMUSG00000046942	Mageb16
ENSMUSG00000046997	Spsb4
ENSMUSG00000047205	Dusp18
ENSMUSG00000047230	Cldn2
ENSMUSG00000047370	Gm7367
ENSMUSG00000047420	Fam180a
ENSMUSG00000047517	Dmbt1
ENSMUSG00000047562	Mmp10
ENSMUSG00000047692	4930533K18Rik
ENSMUSG00000047712	Ust
ENSMUSG00000047730	Fcgbp
ENSMUSG00000047735	Samd9l
ENSMUSG00000047747	Rnf150
ENSMUSG00000047875	Gpr157
ENSMUSG00000047996	Prrg1
ENSMUSG00000048065	Cyb5r2
ENSMUSG00000048087	Gm4737
ENSMUSG00000048126	Col6a3
ENSMUSG00000048164	Gm7204
ENSMUSG00000048185	Gm7075
ENSMUSG00000048188	Gm8181
ENSMUSG00000048261	Gm4879
ENSMUSG00000048334	Gm8258
ENSMUSG00000048402	Gli2
ENSMUSG00000048458	Fam212b
ENSMUSG00000048538	Gm9826
ENSMUSG00000048562	Sp8
ENSMUSG00000048592	Gm5946
ENSMUSG00000048616	Nog
ENSMUSG00000048636	A730049H05Rik
ENSMUSG00000048721	Fndc9
ENSMUSG00000048779	P2ry6
ENSMUSG00000048794	Ccdc37
ENSMUSG00000048852	Gm12185
ENSMUSG00000048960	Prex2
ENSMUSG00000048967	Yjefn3
ENSMUSG00000048988	Elfn1
ENSMUSG00000049001	Ndnf
ENSMUSG00000049044	Rapgef4
ENSMUSG00000049047	Armcx3
ENSMUSG00000049086	Bmyc
ENSMUSG00000049100	Pcdh10
ENSMUSG00000049119	Fam110b
ENSMUSG00000049148	Plcxd3
ENSMUSG00000049230	Gm9833
ENSMUSG00000049281	Scn3b
ENSMUSG00000049313	Sorl1
ENSMUSG00000049409	Prokr1
ENSMUSG00000049414	Gm5417
ENSMUSG00000049422	Chchd10
ENSMUSG00000049460	Gm8396
ENSMUSG00000049511	Htr1b
ENSMUSG00000049515	Espnl
ENSMUSG00000049556	Lingo1
ENSMUSG00000049690	Nckap5
ENSMUSG00000049758	Olfr1318

ENSMUSG00000049791	Fzd4
ENSMUSG00000049804	Armcx4
ENSMUSG00000049999	Ppp1r3d
ENSMUSG00000050050	Ccdc158
ENSMUSG00000050132	Sarm1
ENSMUSG00000050272	Dscam
ENSMUSG00000050288	Fzd2
ENSMUSG00000050345	4930486L24Rik
ENSMUSG00000050359	Sprr1a
ENSMUSG00000050368	Hoxd10
ENSMUSG00000050382	Kif7
ENSMUSG00000050394	Armcx6
ENSMUSG00000050395	Tnfsf15
ENSMUSG00000050490	Gm8394
ENSMUSG00000050578	Mmp13
ENSMUSG00000050640	Tmem150c
ENSMUSG00000050671	Ism2
ENSMUSG00000050776	Olfr1317
ENSMUSG00000050860	Phospho1
ENSMUSG00000051043	Gprc5c
ENSMUSG00000051076	Vtcn1
ENSMUSG00000051176	Zfp42
ENSMUSG00000051177	Plcb1
ENSMUSG00000051242	Pcdhb9
ENSMUSG00000051297	2410124H12Rik
ENSMUSG00000051331	Cacna1c
ENSMUSG00000051359	Ncald
ENSMUSG00000051486	Pcdhb11
ENSMUSG00000051497	Kcnj16
ENSMUSG00000051599	Pcdhb2
ENSMUSG00000051639	Gm5812
ENSMUSG00000051652	Lrrc3
ENSMUSG00000051678	Pcdhb6
ENSMUSG00000051732	Pabpc2
ENSMUSG00000052273	Dnahc3
ENSMUSG00000052353	9930013L23Rik
ENSMUSG00000052373	Mpp3
ENSMUSG00000052374	Actn2
ENSMUSG00000052504	Epha3
ENSMUSG00000052560	Cpne8
ENSMUSG00000052688	5430435G22Rik
ENSMUSG00000052713	Zfp608
ENSMUSG00000052726	Kcnt2
ENSMUSG00000052861	Dnahc6
ENSMUSG00000052889	Prkcb
ENSMUSG00000052920	Prkg1
ENSMUSG00000052955	Cpvl
ENSMUSG00000052957	Gas1
ENSMUSG00000053046	Brsk2
ENSMUSG00000053054	Adh6a
ENSMUSG00000053101	Gpr141
ENSMUSG00000053161	Daw1
ENSMUSG00000053173	Rpl18-ps2
ENSMUSG00000053185	Gm9898
ENSMUSG00000053199	Arhgap20
ENSMUSG00000053216	Btn2a2
ENSMUSG00000053279	Aldh1a1
ENSMUSG00000053395	Cacng8
ENSMUSG00000053414	Hunk
ENSMUSG00000053465	Hs6st3
ENSMUSG00000053472	Gm13534
ENSMUSG00000053475	Tnfaip6
ENSMUSG00000053536	Cstf2t
ENSMUSG00000053541	Gm4759
ENSMUSG00000053613	4732444A12Rik
ENSMUSG00000053617	Sh3pxd2a
ENSMUSG00000053624	Gykl1
ENSMUSG00000053706	B430305J03Rik
ENSMUSG00000053797	Krt16
ENSMUSG00000054000	Tusc1
ENSMUSG00000054072	Iigp1
ENSMUSG00000054128	H2-T3

ENSMUSG00000054256	Msi1
ENSMUSG00000054342	Kcnn4
ENSMUSG00000054418	2900041M22Rik
ENSMUSG00000054545	Ugt1a6a
ENSMUSG00000054589	Gm9949
ENSMUSG00000054612	Mgmt
ENSMUSG00000054630	Ugt2b5
ENSMUSG00000054640	Slc8a1
ENSMUSG00000054659	Pm20d2
ENSMUSG00000054667	Irs4
ENSMUSG00000054763	Defb42
ENSMUSG00000054863	Fam19a5
ENSMUSG00000054934	Kcnmb4
ENSMUSG00000054942	Fam73a
ENSMUSG00000055027	Smyd1
ENSMUSG00000055053	Nfic
ENSMUSG00000055069	Rab39
ENSMUSG00000055150	Zfp78
ENSMUSG00000055172	C1ra
ENSMUSG00000055322	Tns1
ENSMUSG00000055413	H2-Q5
ENSMUSG00000055421	Pcdh9
ENSMUSG00000055435	Maf
ENSMUSG00000055538	Zcchc24
ENSMUSG00000055632	Hmcn2
ENSMUSG00000055653	Gpc3
ENSMUSG00000055675	Kbtbd11
ENSMUSG00000055692	Tmem191c
ENSMUSG00000055771	Gm7936
ENSMUSG00000055780	Usp26
ENSMUSG00000055799	Tcf7l1
ENSMUSG00000056091	St3gal5
ENSMUSG00000056214	Pard6g
ENSMUSG00000056220	Pla2g4a
ENSMUSG00000056270	Prr9
ENSMUSG00000056290	Ms4a4b
ENSMUSG00000056367	Actr3b
ENSMUSG00000056476	Med12l
ENSMUSG00000056486	Chn1
ENSMUSG00000056602	Fry
ENSMUSG00000056643	Chst13
ENSMUSG00000056772	Rps6-ps2
ENSMUSG00000056870	Gulp1
ENSMUSG00000056877	Rps15a-ps1
ENSMUSG00000056900	Usp13
ENSMUSG00000057098	Ebf1
ENSMUSG00000057182	Scn3a
ENSMUSG00000057315	Arhgap24
ENSMUSG00000057321	Usp17ld
ENSMUSG00000057346	Apol9a
ENSMUSG00000057425	Ugt2b37
ENSMUSG00000057596	Trim30d
ENSMUSG00000057614	Gnai1
ENSMUSG00000057657	Rps18-ps3
ENSMUSG00000057706	Mex3b
ENSMUSG00000057895	Zfp105
ENSMUSG00000057914	Cacnb2
ENSMUSG00000057933	Gsta2
ENSMUSG00000058070	Eml1
ENSMUSG00000058159	T2
ENSMUSG00000058163	Gm5431
ENSMUSG00000058400	Qrfpr
ENSMUSG00000058447	Zfp82
ENSMUSG00000058470	Gm8369
ENSMUSG00000058581	Gm5801
ENSMUSG00000058590	Gm6462
ENSMUSG00000058624	Gda
ENSMUSG00000058628	Olfr875
ENSMUSG00000058838	Rps27a-ps2
ENSMUSG00000058914	C1qtnf3
ENSMUSG00000058975	Kcnc1
ENSMUSG00000059022	Kcp

ENSMUSG00000059033	Rpl18a-ps1
ENSMUSG00000059040	Gm5506
ENSMUSG00000059089	Fcgr4
ENSMUSG00000059326	Csf2ra
ENSMUSG00000059343	Aldoart1
ENSMUSG00000059552	Trp53
ENSMUSG00000059658	Gm16379
ENSMUSG00000059751	Rps3a3
ENSMUSG00000059775	Rps26-ps1
ENSMUSG00000059776	Gm10071
ENSMUSG00000059895	Ptp4a3
ENSMUSG00000059901	Adamts14
ENSMUSG00000060063	Alox5ap
ENSMUSG00000060275	Nrg2
ENSMUSG00000060317	Acnat2
ENSMUSG00000060371	Caln1
ENSMUSG00000060429	Sntb1
ENSMUSG00000060499	Rpl10l
ENSMUSG00000060512	0610040J01Rik
ENSMUSG00000060548	Tnfrsf19
ENSMUSG00000060795	Gm13363
ENSMUSG00000060988	Galnt13
ENSMUSG00000061186	Sfmbt2
ENSMUSG00000061272	Gm14173
ENSMUSG00000061330	Gm11361
ENSMUSG00000061411	8430427H17Rik
ENSMUSG00000061451	Tmem151a
ENSMUSG00000061486	Gm5161
ENSMUSG00000061488	Rpl27a-ps1
ENSMUSG00000061584	Lyg2
ENSMUSG00000061601	Pclo
ENSMUSG00000061669	Gm6404
ENSMUSG00000061751	Kalrn
ENSMUSG00000061815	Rufy4
ENSMUSG00000061848	Gm5805
ENSMUSG00000061947	Serpina10
ENSMUSG00000061988	Rpl10a-ps2
ENSMUSG00000062078	Qk
ENSMUSG00000062157	Ifnlr1
ENSMUSG00000062235	Gm5341
ENSMUSG00000062279	Gm10237
ENSMUSG00000062458	Gm8623
ENSMUSG00000062472	Gm4945
ENSMUSG00000062477	Hmgb1l
ENSMUSG00000062551	Prl2c1
ENSMUSG00000062563	Cys1
ENSMUSG00000062588	Gm6104
ENSMUSG00000062727	Hist1h2bk
ENSMUSG00000062758	Gm16477
ENSMUSG00000062861	Zfp28
ENSMUSG00000062991	Nrg1
ENSMUSG00000063063	Ctnna2
ENSMUSG00000063129	Aldoart2
ENSMUSG00000063166	Gm5449
ENSMUSG00000063180	Gm10126
ENSMUSG00000063193	Cd300lb
ENSMUSG00000063245	Gm13248
ENSMUSG00000063286	Gm8995
ENSMUSG00000063296	Tmem117
ENSMUSG00000063350	Olfr874
ENSMUSG00000063415	Cyp26b1
ENSMUSG00000063543	Gm5616
ENSMUSG00000063548	Gm4825
ENSMUSG00000063556	Gm10132
ENSMUSG00000063568	Jazf1
ENSMUSG00000063586	Gm5513
ENSMUSG00000063687	Pcdhb5
ENSMUSG00000063696	Gm8730
ENSMUSG00000063873	Slc24a3
ENSMUSG00000063875	Rps6-ps1
ENSMUSG00000063919	Srrm4
ENSMUSG00000064043	Trerf1

ENSMUSG00000064063	BC048507
ENSMUSG00000064281	Rpl19-ps1
ENSMUSG00000064325	Hhip
ENSMUSG00000064354	mt-Co2
ENSMUSG00000064355	mt-Tk
ENSMUSG00000064373	Sepp1
ENSMUSG00000065126	Snord104
ENSMUSG00000066026	Dhrs3
ENSMUSG00000066068	Gm13611
ENSMUSG00000066071	Cyp4a12a
ENSMUSG00000066362	Rps13-ps1
ENSMUSG00000066456	Hmgn3
ENSMUSG00000066478	Gm5745
ENSMUSG00000066554	Gm10167
ENSMUSG00000066629	Rpl36-ps3
ENSMUSG00000066755	Tnfsf18
ENSMUSG00000066807	Gm10179
ENSMUSG00000066809	Gm10180
ENSMUSG00000066842	Hmcn1
ENSMUSG00000066983	Gm16519
ENSMUSG00000067001	Serpinb7
ENSMUSG00000067158	Col4a4
ENSMUSG00000067261	Foxd3
ENSMUSG00000067351	Rps15a-ps2
ENSMUSG00000067377	Tspan6
ENSMUSG00000067575	Rpl35a-ps3
ENSMUSG00000067577	A430093F15Rik
ENSMUSG00000067860	Zic3
ENSMUSG00000068105	Tnfrsf13c
ENSMUSG00000068227	Il2rb
ENSMUSG00000068246	Apol9b
ENSMUSG00000068262	Gm5879
ENSMUSG00000068396	Rpl34-ps1
ENSMUSG00000068480	Gm7551
ENSMUSG00000068551	Zfp467
ENSMUSG00000068614	Actc1
ENSMUSG00000068744	Psrc1
ENSMUSG00000068758	Il3ra
ENSMUSG00000068794	Col28a1
ENSMUSG00000069044	Usp9y
ENSMUSG00000069255	Dusp22
ENSMUSG00000069265	Hist1h3a
ENSMUSG00000069300	Hist1h2bj
ENSMUSG00000069301	Hist1h2ag
ENSMUSG00000069662	Marcks
ENSMUSG00000069892	9930111J21Rik2
ENSMUSG00000069893	9930111J21Rik1
ENSMUSG00000069899	Gm12166
ENSMUSG00000069939	Gm12070
ENSMUSG00000069962	Gm5356
ENSMUSG00000069996	Gm7125
ENSMUSG00000070280	Slc22a14
ENSMUSG00000070323	Mmp27
ENSMUSG00000070473	Cldn3
ENSMUSG00000070476	Fam217b
ENSMUSG00000070509	Rgma
ENSMUSG00000070524	Fcrlb
ENSMUSG00000070605	Gm13251
ENSMUSG00000070667	Rpl31-ps10
ENSMUSG00000070695	Cntnap5a
ENSMUSG00000070704	Ugt2b36
ENSMUSG00000070713	Gm10282
ENSMUSG00000070778	Rpl7a-ps8
ENSMUSG00000070867	Trabd2b
ENSMUSG00000071040	Gm9386
ENSMUSG00000071217	Gm2022
ENSMUSG00000071237	Gm6253
ENSMUSG00000071273	Gm5145
ENSMUSG00000071303	Rps8-ps1
ENSMUSG00000071362	Gm10330
ENSMUSG00000071419	Rps15-ps2
ENSMUSG00000071470	Ccnb1ip1

ENSMUSG00000071475	Rpl21-ps5
ENSMUSG00000071516	Hist1h2ai
ENSMUSG00000071532	Gm10335
ENSMUSG00000071568	Gm5874
ENSMUSG00000071604	Fam189a2
ENSMUSG00000071713	Csf2rb
ENSMUSG00000071715	Ncf4
ENSMUSG00000071719	Tmem28
ENSMUSG00000072244	Trim6
ENSMUSG00000072294	Klf12
ENSMUSG00000072324	Gm8420
ENSMUSG00000072407	Gm6419
ENSMUSG00000072568	Fam84b
ENSMUSG00000072680	Tmem254c
ENSMUSG00000072875	Gpr27
ENSMUSG00000072941	Sod3
ENSMUSG00000072949	Acot1
ENSMUSG00000073052	D130052B06Rik
ENSMUSG00000073067	9130019P16Rik
ENSMUSG00000073233	Gm9144
ENSMUSG00000073295	Nudt11
ENSMUSG00000073402	Gm8909
ENSMUSG00000073403	Gm10499
ENSMUSG00000073409	H2-Q6
ENSMUSG00000073489	Ifi204
ENSMUSG00000073530	Pappa2
ENSMUSG00000073535	Gm5532
ENSMUSG00000073557	Ppp1r12b
ENSMUSG00000073679	Gm10562
ENSMUSG00000073716	Gm13241
ENSMUSG00000073728	Tmem51as1
ENSMUSG00000073844	Gm21957
ENSMUSG00000074026	Gm6091
ENSMUSG00000074158	9830147E19Rik
ENSMUSG00000074384	AI429214
ENSMUSG00000074403	Hist2h3b
ENSMUSG00000074505	Fat3
ENSMUSG00000074516	Gm10709
ENSMUSG00000074553	Gm10713
ENSMUSG00000074575	Kcng1
ENSMUSG00000074577	Fam65c
ENSMUSG00000074620	Gm10731
ENSMUSG00000074622	Mafb
ENSMUSG00000074623	Gm826
ENSMUSG00000074634	Gm7120
ENSMUSG00000074635	3110070M22Rik
ENSMUSG00000074637	Sox2
ENSMUSG00000074682	Zcchc3
ENSMUSG00000074780	Anapc15-ps
ENSMUSG00000074903	Gm2058
ENSMUSG00000074930	Gm13981
ENSMUSG00000075012	Fjx1
ENSMUSG00000075028	Prdm11
ENSMUSG00000075122	Cd80
ENSMUSG00000075277	6720416L17Rik
ENSMUSG00000075297	H60b
ENSMUSG00000075302	Erich2
ENSMUSG00000075304	Sp5
ENSMUSG00000075324	Fign
ENSMUSG00000075525	Gm10849
ENSMUSG00000075581	Gm16409
ENSMUSG00000075609	Gm7935
ENSMUSG00000075702	Selm
ENSMUSG00000076036	Gm22133
ENSMUSG00000076230	Gm22758
ENSMUSG00000076441	Ass1
ENSMUSG00000076677	Ighv6-3
ENSMUSG00000078128	Gm2178
ENSMUSG00000078134	Gm12355
ENSMUSG00000078153	Psme2b-ps
ENSMUSG00000078183	Gm15610
ENSMUSG00000078184	B020018G12Rik

ENSMUSG00000078234	Klhdc7a
ENSMUSG00000078302	Foxd1
ENSMUSG00000078435	AU041133
ENSMUSG00000078452	Raet1d
ENSMUSG00000078532	Nkain1
ENSMUSG00000078597	Cyp4a12b
ENSMUSG00000078606	Gm4070
ENSMUSG00000078636	Gm7336
ENSMUSG00000078684	5830417I10Rik
ENSMUSG00000078880	Gm14308
ENSMUSG00000078920	Ifi47
ENSMUSG00000078921	Tgtp2
ENSMUSG00000078922	Tgtp1
ENSMUSG00000078965	Gm12033
ENSMUSG00000079262	Slco1a6
ENSMUSG00000079297	Gm2223
ENSMUSG00000079339	Gm14446
ENSMUSG00000079359	Gm9006
ENSMUSG00000079387	Luzp4
ENSMUSG00000079462	Gm15737
ENSMUSG00000079499	6530402F18Rik
ENSMUSG00000079507	H2-Q1
ENSMUSG00000079559	Gm684
ENSMUSG00000079915	Gm16073
ENSMUSG00000079941	Gm11273
ENSMUSG00000079962	Gm12643
ENSMUSG00000079963	Gm7146
ENSMUSG00000079998	Gm12947
ENSMUSG00000080002	Gm5566
ENSMUSG00000080242	Gm15487
ENSMUSG00000080708	Ccnd3-ps
ENSMUSG00000080710	Gm8107
ENSMUSG00000080718	Gm8019
ENSMUSG00000080747	Gm14016
ENSMUSG00000080759	Gm15573
ENSMUSG00000080763	Gm12341
ENSMUSG00000080773	Gm12955
ENSMUSG00000080778	Gm7199
ENSMUSG00000080779	Gm8731
ENSMUSG00000080782	Gm13495
ENSMUSG00000080788	Gm7841
ENSMUSG00000080790	Gm7638
ENSMUSG00000080796	Gm9159
ENSMUSG00000080808	Gm12608
ENSMUSG00000080818	Gm9434
ENSMUSG00000080825	Gm14892
ENSMUSG00000080829	Gm8545
ENSMUSG00000080844	Gm14107
ENSMUSG00000080845	Gm9115
ENSMUSG00000080846	Gm13379
ENSMUSG00000080855	Rpl35-ps1
ENSMUSG00000080859	Rpl10-ps1
ENSMUSG00000080860	Gm13815
ENSMUSG00000080893	Gm15920
ENSMUSG00000080922	Gm2214
ENSMUSG00000080923	Gm5400
ENSMUSG00000080928	Hmgb1-ps6
ENSMUSG00000080932	Gm10224
ENSMUSG00000080966	Gm6263
ENSMUSG00000080969	Gm12372
ENSMUSG00000080972	Gm16061
ENSMUSG00000080980	1700071K01Rik
ENSMUSG00000080982	Gm13549
ENSMUSG00000080989	Gm14048
ENSMUSG00000080994	Gm13464
ENSMUSG00000080998	Gm5384
ENSMUSG00000081009	Gm14065
ENSMUSG00000081023	Gm15766
ENSMUSG00000081026	Gm11855
ENSMUSG00000081049	Rps24-ps3
ENSMUSG00000081050	Gm12461
ENSMUSG00000081067	Gm12912

ENSMUSG00000081068	Gm12804
ENSMUSG00000081070	Gm13181
ENSMUSG00000081084	Gm5638
ENSMUSG00000081099	Gm5270
ENSMUSG00000081113	Gm7308
ENSMUSG00000081120	Gm12458
ENSMUSG00000081130	Gm8217
ENSMUSG00000081138	Gm12834
ENSMUSG00000081145	Gm14585
ENSMUSG00000081152	Gm12430
ENSMUSG00000081164	Gm8722
ENSMUSG00000081168	Gm13730
ENSMUSG00000081173	Gm13500
ENSMUSG00000081182	Gm14935
ENSMUSG00000081189	Gm12725
ENSMUSG00000081205	Gm5940
ENSMUSG00000081210	Gm12933
ENSMUSG00000081214	Rpl35a-ps2
ENSMUSG00000081221	Gm14760
ENSMUSG00000081230	Gm9097
ENSMUSG00000081231	Tcp1-ps1
ENSMUSG00000081233	Gm12857
ENSMUSG00000081235	Gm14788
ENSMUSG00000081239	Gm11836
ENSMUSG00000081242	Gm15719
ENSMUSG00000081249	Gm11517
ENSMUSG00000081255	Gm5380
ENSMUSG00000081273	Gm15896
ENSMUSG00000081289	Gm14857
ENSMUSG00000081295	Gm6275
ENSMUSG00000081308	Gm14480
ENSMUSG00000081319	Gm12936
ENSMUSG00000081350	Gm5388
ENSMUSG00000081364	Gm8595
ENSMUSG00000081384	Gm12752
ENSMUSG00000081389	Gm13368
ENSMUSG00000081390	Gm14538
ENSMUSG00000081391	Gm11765
ENSMUSG00000081394	Gm13215
ENSMUSG00000081396	Gm15132
ENSMUSG00000081400	Gm13680
ENSMUSG00000081402	Gm15455
ENSMUSG00000081423	Gm12882
ENSMUSG00000081431	Gm15483
ENSMUSG00000081432	Gm12593
ENSMUSG00000081441	Gm6161
ENSMUSG00000081448	Gm13086
ENSMUSG00000081456	Gm7781
ENSMUSG00000081470	Gm14131
ENSMUSG00000081473	Gm4853
ENSMUSG00000081476	Itpa-ps1
ENSMUSG00000081488	Gm15368
ENSMUSG00000081496	Gm13428
ENSMUSG00000081505	Gm8644
ENSMUSG00000081519	Gm13331
ENSMUSG00000081520	Gm16200
ENSMUSG00000081526	Gm7219
ENSMUSG00000081544	Gm13077
ENSMUSG00000081546	Gm14927
ENSMUSG00000081557	Gm5697
ENSMUSG00000081562	Gm11575
ENSMUSG00000081572	Gm14138
ENSMUSG00000081584	Gm12716
ENSMUSG00000081614	Gm9436
ENSMUSG00000081643	Gm11605
ENSMUSG00000081648	Gm13423
ENSMUSG00000081654	Gm5383
ENSMUSG00000081657	Gm15466
ENSMUSG00000081661	Gm14450
ENSMUSG00000081664	Gm15544
ENSMUSG00000081669	Npm3-ps1
ENSMUSG00000081683	Fzd10

ENSMUSG00000081684	Rps2-ps13
ENSMUSG00000081695	Rpsa-ps5
ENSMUSG00000081705	Gm12582
ENSMUSG00000081718	Gm8261
ENSMUSG00000081729	Hspa9-ps1
ENSMUSG00000081732	Gm15495
ENSMUSG00000081757	Gm13798
ENSMUSG00000081769	Gm12216
ENSMUSG00000081772	Gm13131
ENSMUSG00000081783	Gm15599
ENSMUSG00000081787	Gm13991
ENSMUSG00000081789	Gm11893
ENSMUSG00000081796	Gm9005
ENSMUSG00000081797	Gm8662
ENSMUSG00000081819	Gm12722
ENSMUSG00000081840	Gm15220
ENSMUSG00000081850	Gm15667
ENSMUSG00000081871	Gm11488
ENSMUSG00000081875	Gm15223
ENSMUSG00000081877	Gm8858
ENSMUSG00000081881	Gm14752
ENSMUSG00000081883	Gm15574
ENSMUSG00000081885	Gm13231
ENSMUSG00000081892	Gm8864
ENSMUSG00000081896	Gm5389
ENSMUSG00000081906	Rpl9-ps1
ENSMUSG00000081924	Gm11191
ENSMUSG00000081926	Gm15536
ENSMUSG00000081929	Rps11-ps2
ENSMUSG00000081932	Gm12331
ENSMUSG00000081941	Gm11218
ENSMUSG00000081943	Gm9078
ENSMUSG00000081946	Gm11472
ENSMUSG00000081951	Gm15352
ENSMUSG00000081953	Gm12435
ENSMUSG00000081975	Gm12482
ENSMUSG00000081984	Dnajb3
ENSMUSG00000081992	Gm13408
ENSMUSG00000081994	Gm14517
ENSMUSG00000082013	Gm13216
ENSMUSG00000082016	Pgam1-ps2
ENSMUSG00000082026	Gm5764
ENSMUSG00000082039	Gm15653
ENSMUSG00000082043	Gm12848
ENSMUSG00000082044	Gm14284
ENSMUSG00000082052	Gm13819
ENSMUSG00000082061	Gm12726
ENSMUSG00000082062	Ftl2
ENSMUSG00000082063	Gm12993
ENSMUSG00000082066	Gm13904
ENSMUSG00000082076	Gm8659
ENSMUSG00000082087	Gm12138
ENSMUSG00000082097	Gm14039
ENSMUSG00000082100	Glns-ps1
ENSMUSG00000082107	Gm16216
ENSMUSG00000082109	Rpl19-ps12
ENSMUSG00000082128	Gm11824
ENSMUSG00000082136	Gm15660
ENSMUSG00000082143	Gm12864
ENSMUSG00000082151	Gm11810
ENSMUSG00000082171	Rpl27-ps2
ENSMUSG00000082174	Gm14877
ENSMUSG00000082175	Gm14563
ENSMUSG00000082176	Gm13163
ENSMUSG00000082177	Gm16005
ENSMUSG00000082185	Gm12428
ENSMUSG00000082189	Gm4784
ENSMUSG00000082198	Gm14504
ENSMUSG00000082202	Gm4910
ENSMUSG00000082235	Gm13326
ENSMUSG00000082253	Gm14639
ENSMUSG00000082254	Gm11922

ENSMUSG00000082255	Gm11822
ENSMUSG00000082258	Gm15289
ENSMUSG00000082269	Gm14676
ENSMUSG00000082272	Gm11675
ENSMUSG00000082284	H3f3a-ps1
ENSMUSG00000082289	Gm15596
ENSMUSG00000082308	Gm15770
ENSMUSG00000082312	Gm11996
ENSMUSG00000082336	Gm8401
ENSMUSG00000082341	Gm14143
ENSMUSG00000082348	Gm12807
ENSMUSG00000082390	Gm12136
ENSMUSG00000082399	Gm14036
ENSMUSG00000082415	Gm12486
ENSMUSG00000082456	Gm11598
ENSMUSG00000082457	Gm13776
ENSMUSG00000082458	Gm9430
ENSMUSG00000082461	Gm8844
ENSMUSG00000082478	Gm11619
ENSMUSG00000082480	Gm11687
ENSMUSG00000082481	Rps12-ps19
ENSMUSG00000082510	Gm11793
ENSMUSG00000082513	Gm15374
ENSMUSG00000082514	Gm11452
ENSMUSG00000082523	Gm12667
ENSMUSG00000082530	Gm12168
ENSMUSG00000082535	Gm7860
ENSMUSG00000082543	Gm14336
ENSMUSG00000082545	Gm12196
ENSMUSG00000082567	Gm14896
ENSMUSG00000082578	Gm13527
ENSMUSG00000082580	Gm13182
ENSMUSG00000082600	Gm9673
ENSMUSG00000082605	Gm16148
ENSMUSG00000082609	Gm15464
ENSMUSG00000082621	Rpl7a-ps13
ENSMUSG00000082636	Gm14923
ENSMUSG00000082646	Gm12732
ENSMUSG00000082647	Gm14056
ENSMUSG00000082649	Gm12754
ENSMUSG00000082669	Gm5393
ENSMUSG00000082675	Gm6382
ENSMUSG00000082677	Gm15371
ENSMUSG00000082679	Gm13469
ENSMUSG00000082682	Gm15349
ENSMUSG00000082683	Gm12697
ENSMUSG00000082688	Gm12124
ENSMUSG00000082689	Gm14156
ENSMUSG00000082691	Dynlt1-ps1
ENSMUSG00000082693	Gm15190
ENSMUSG00000082701	Gm14460
ENSMUSG00000082743	Gm12988
ENSMUSG00000082749	Rpl9-ps2
ENSMUSG00000082755	Gm8692
ENSMUSG00000082776	Gm7061
ENSMUSG00000082793	Gm9308
ENSMUSG00000082794	Gm6806
ENSMUSG00000082804	Rpl17-ps1
ENSMUSG00000082835	Gm13118
ENSMUSG00000082836	Gm13612
ENSMUSG00000082849	Gm14053
ENSMUSG00000082855	Gm14537
ENSMUSG00000082862	Gm13806
ENSMUSG00000082870	Gm12165
ENSMUSG00000082872	Gm15773
ENSMUSG00000082874	Rpl15-ps1
ENSMUSG00000082876	Gm11889
ENSMUSG00000082896	Gm5844
ENSMUSG00000082905	Gm7823
ENSMUSG00000082911	Gm14119
ENSMUSG00000082924	Gm11462
ENSMUSG00000082928	Gm12785

ENSMUSG00000082929	Gm6067
ENSMUSG00000082931	Gm13586
ENSMUSG00000082935	Gm7658
ENSMUSG00000082950	Gm7834
ENSMUSG00000082951	Gm13359
ENSMUSG00000082953	Gm13217
ENSMUSG00000082964	Rpl13-ps5
ENSMUSG00000082974	Gm15129
ENSMUSG00000082978	Rpsa-ps11
ENSMUSG00000082987	Rps13-ps6
ENSMUSG00000082991	Rps10-ps3
ENSMUSG00000082994	Gm11848
ENSMUSG00000083011	Gm12816
ENSMUSG00000083025	Gm11829
ENSMUSG00000083029	Gm13720
ENSMUSG00000083075	Gm11980
ENSMUSG00000083089	Gm4760
ENSMUSG00000083103	Gm6429
ENSMUSG00000083116	Gm13410
ENSMUSG00000083121	Gm6341
ENSMUSG00000083122	Gm15578
ENSMUSG00000083136	Gm15170
ENSMUSG00000083154	Gm11892
ENSMUSG00000083159	Gm12180
ENSMUSG00000083160	Gm15967
ENSMUSG00000083166	Gm8648
ENSMUSG00000083177	Gm7820
ENSMUSG00000083183	Gm15575
ENSMUSG00000083186	Gm11805
ENSMUSG00000083218	Gm16425
ENSMUSG00000083224	Gm11255
ENSMUSG00000083235	Gm13246
ENSMUSG00000083238	Hspe1-ps4
ENSMUSG00000083240	Gm13453
ENSMUSG00000083255	Gm12738
ENSMUSG00000083261	Gm7816
ENSMUSG00000083262	Gm14470
ENSMUSG00000083270	Gm13498
ENSMUSG00000083282	Ctsf
ENSMUSG00000083291	Gm13679
ENSMUSG00000083306	Gm13868
ENSMUSG00000083311	Gm5643
ENSMUSG00000083320	Gm13935
ENSMUSG00000083325	Gm14121
ENSMUSG00000083344	Gm7363
ENSMUSG00000083346	Gm8260
ENSMUSG00000083355	Gm11581
ENSMUSG00000083367	Gm8806
ENSMUSG00000083390	Gm13121
ENSMUSG00000083394	Gm11703
ENSMUSG00000083395	Gm11203
ENSMUSG00000083399	Gm15033
ENSMUSG00000083405	Gm15725
ENSMUSG00000083421	Gm14776
ENSMUSG00000083422	Gm15604
ENSMUSG00000083424	Rpl35a-ps4
ENSMUSG00000083429	Gm15198
ENSMUSG00000083431	Gm13437
ENSMUSG00000083440	Rpl7a-ps12
ENSMUSG00000083458	Gm5510
ENSMUSG00000083468	Gm9431
ENSMUSG00000083483	Gm14044
ENSMUSG00000083488	Gm8534
ENSMUSG00000083498	Gm14172
ENSMUSG00000083536	Gm15808
ENSMUSG00000083544	Oat-rs1
ENSMUSG00000083573	Gm7312
ENSMUSG00000083580	Rpsa-ps3
ENSMUSG00000083588	Gm8051
ENSMUSG00000083617	Gm5390
ENSMUSG00000083619	Gm14414
ENSMUSG00000083621	Gm14586

ENSMUSG00000083633	Gm13312
ENSMUSG00000083642	Gm13148
ENSMUSG00000083649	Rasl2-9
ENSMUSG00000083654	Gm11875
ENSMUSG00000083655	Gm12099
ENSMUSG00000083662	Gm12152
ENSMUSG00000083669	Gm10169
ENSMUSG00000083679	Gm12892
ENSMUSG00000083681	Gm6640
ENSMUSG00000083684	Gm14034
ENSMUSG00000083689	Gm14094
ENSMUSG00000083693	Gm11971
ENSMUSG00000083716	Gm13436
ENSMUSG00000083720	Gm12901
ENSMUSG00000083728	Gm13854
ENSMUSG00000083737	Prdx6-ps2
ENSMUSG00000083748	Gm11662
ENSMUSG00000083760	Gm14524
ENSMUSG00000083761	Pgam1-ps1
ENSMUSG00000083766	Gm15335
ENSMUSG00000083767	Gm11405
ENSMUSG00000083773	Gm13394
ENSMUSG00000083774	Gm7180
ENSMUSG00000083783	Gm12739
ENSMUSG00000083792	Rpl12-ps2
ENSMUSG00000083804	Gm13676
ENSMUSG00000083819	Gm11977
ENSMUSG00000083831	Gm14248
ENSMUSG00000083837	Gm14427
ENSMUSG00000083838	Gm11957
ENSMUSG00000083852	Gm13007
ENSMUSG00000083864	Gm13719
ENSMUSG00000083875	Gm13831
ENSMUSG00000083878	Gm12034
ENSMUSG00000083880	Hspe1-ps6
ENSMUSG00000083912	Gm5391
ENSMUSG00000083919	Tpi-rs8
ENSMUSG00000083937	Cct3-ps1
ENSMUSG00000083939	Gm14451
ENSMUSG00000083948	Gm7158
ENSMUSG00000083985	Gm12468
ENSMUSG00000083988	Rps10-ps4
ENSMUSG00000084012	Gm11956
ENSMUSG00000084018	Gm11895
ENSMUSG00000084021	Gm5832
ENSMUSG00000084022	Gm16459
ENSMUSG00000084024	Gm15937
ENSMUSG00000084031	Gm7416
ENSMUSG00000084047	Gm14984
ENSMUSG00000084065	Gm15159
ENSMUSG00000084073	Gm11910
ENSMUSG00000084086	Gm5761
ENSMUSG00000084094	Gm13961
ENSMUSG00000084099	Gm12929
ENSMUSG00000084104	Gm13578
ENSMUSG00000084119	Gm12351
ENSMUSG00000084131	Rpl3-ps2
ENSMUSG00000084140	Gm14000
ENSMUSG00000084153	Gm12186
ENSMUSG00000084162	Gm11251
ENSMUSG00000084177	Gm13508
ENSMUSG00000084183	Gm12009
ENSMUSG00000084195	Gm15372
ENSMUSG00000084197	Gm9089
ENSMUSG00000084215	Gm11814
ENSMUSG00000084216	Gm7973
ENSMUSG00000084218	Gm8145
ENSMUSG00000084249	Gm12267
ENSMUSG00000084278	Gm12189
ENSMUSG00000084280	Gm11972
ENSMUSG00000084301	Gm7856
ENSMUSG00000084302	Gm12194

ENSMUSG00000084306	Gm11743
ENSMUSG00000084328	Gm14046
ENSMUSG00000084331	Gm8163
ENSMUSG00000084333	Gm11599
ENSMUSG00000084345	Rpl17-ps4
ENSMUSG00000084347	Akt2-ps
ENSMUSG00000084350	Znf41-ps
ENSMUSG00000084413	Gm12017
ENSMUSG00000084822	Gm11768
ENSMUSG00000084838	Gm10241
ENSMUSG00000084849	Gm16105
ENSMUSG00000084874	Gm12906
ENSMUSG00000084989	E030010N08Rik
ENSMUSG00000085055	Gm15958
ENSMUSG00000085058	8030453O22Rik
ENSMUSG00000085073	Gm14562
ENSMUSG00000085155	Gm11780
ENSMUSG00000085162	Gm12295
ENSMUSG00000085172	Gm6542
ENSMUSG00000085281	Gm13316
ENSMUSG00000085300	Gm16345
ENSMUSG00000085440	D330022K07Rik
ENSMUSG00000085465	Gm15347
ENSMUSG00000085514	9530048J24Rik
ENSMUSG00000085525	Gm13166
ENSMUSG00000085587	Gm14493
ENSMUSG00000085604	Gm11547
ENSMUSG00000085620	G630018N14Rik
ENSMUSG00000085629	Gm11697
ENSMUSG00000085683	9130409J20Rik
ENSMUSG00000085707	Gm12212
ENSMUSG00000085715	Tsix
ENSMUSG00000085747	Gm11194
ENSMUSG00000085837	4933400F03Rik
ENSMUSG00000085862	Gm13483
ENSMUSG00000085867	Gm5834
ENSMUSG00000085897	Gm11604
ENSMUSG00000085941	Gm11201
ENSMUSG00000085981	Gm11375
ENSMUSG00000086003	B230206L02Rik
ENSMUSG00000086006	Gm13293
ENSMUSG00000086040	Wipf3
ENSMUSG00000086095	Gm15328
ENSMUSG00000086191	1110035M17Rik
ENSMUSG00000086213	A330040F15Rik
ENSMUSG00000086240	Gm7846
ENSMUSG00000086245	Gm16170
ENSMUSG00000086268	Gm11670
ENSMUSG00000086313	Gm15940
ENSMUSG00000086416	Gm14002
ENSMUSG00000086426	Gm15295
ENSMUSG00000086503	Xist
ENSMUSG00000086513	9130208D14Rik
ENSMUSG00000086515	Gm16292
ENSMUSG00000086537	Nespas
ENSMUSG00000086544	Gm13704
ENSMUSG00000086560	Gm13372
ENSMUSG00000086567	Gm2830
ENSMUSG00000086587	Gm11837
ENSMUSG00000086665	Gm13067
ENSMUSG00000086670	Gm13194
ENSMUSG00000086743	Hnrnpa1l2-ps
ENSMUSG00000086746	Gm15222
ENSMUSG00000086784	Isoc2a
ENSMUSG00000086796	4932702P03Rik
ENSMUSG00000086926	Gm6226
ENSMUSG00000086946	Gm15527
ENSMUSG00000087002	Gm16277
ENSMUSG00000087095	Emx2os
ENSMUSG00000087179	Gm14230
ENSMUSG00000087187	Gm13431
ENSMUSG00000087220	Gm11377

ENSMUSG00000087239	Gm7613
ENSMUSG00000087255	Gm6985
ENSMUSG00000087264	1500002O10Rik
ENSMUSG00000087265	Gm12349
ENSMUSG00000087278	A930006I01Rik
ENSMUSG00000087321	Gm5353
ENSMUSG00000087400	Gm15270
ENSMUSG00000087415	Gm14529
ENSMUSG00000087461	C230014O12Rik
ENSMUSG00000087503	Gm11459
ENSMUSG00000087613	Gm13855
ENSMUSG00000087651	1500009L16Rik
ENSMUSG00000087993	Mir1982
ENSMUSG00000088529	Gm26083
ENSMUSG00000089278	Gm24553
ENSMUSG00000089628	Gm9727
ENSMUSG00000089639	Gm2862
ENSMUSG00000089645	Gm5766
ENSMUSG00000089665	Fcor
ENSMUSG00000089673	Gm16546
ENSMUSG00000089762	Ier5l
ENSMUSG00000089785	Gm8429
ENSMUSG00000089803	Gm10171
ENSMUSG00000089807	Gm16298
ENSMUSG00000089829	Gm16565
ENSMUSG00000089834	Gm16303
ENSMUSG00000089837	Npcd
ENSMUSG00000089838	Gm2962
ENSMUSG00000089868	Gm16134
ENSMUSG00000089901	Gm8113
ENSMUSG00000089908	Gm16558
ENSMUSG00000089943	Ugt1a5
ENSMUSG00000089945	Gm20459
ENSMUSG00000089960	Ugt1a1
ENSMUSG00000089988	Gm16238
ENSMUSG00000090007	Rpl30-ps2
ENSMUSG00000090053	Palm2
ENSMUSG00000090099	Gm8837
ENSMUSG00000090124	Ugt1a7c
ENSMUSG00000090126	4930519F09Rik
ENSMUSG00000090138	Gm3687
ENSMUSG00000090142	Gm15795
ENSMUSG00000090145	Ugt1a6b
ENSMUSG00000090147	Gm3329
ENSMUSG00000090171	Ugt1a2
ENSMUSG00000090234	Gm16042
ENSMUSG00000090243	Gm16103
ENSMUSG00000090272	Mndal
ENSMUSG00000090277	Gm8338
ENSMUSG00000090307	1700071M16Rik
ENSMUSG00000090322	Gm17090
ENSMUSG00000090323	Gm5263
ENSMUSG00000090369	4933411K16Rik
ENSMUSG00000090451	Gm6133
ENSMUSG00000090460	Gm17511
ENSMUSG00000090467	Gm4613
ENSMUSG00000090474	Gm9372
ENSMUSG00000090489	Gm17415
ENSMUSG00000090538	Gm4775
ENSMUSG00000090570	Gm17041
ENSMUSG00000090602	Gm5611
ENSMUSG00000090624	Gm17060
ENSMUSG00000090639	Gm20425
ENSMUSG00000090659	Zfp493
ENSMUSG00000090674	Gm17082
ENSMUSG00000090714	Zscan4d
ENSMUSG00000090753	Rpl31-ps4
ENSMUSG00000090955	Gm17097
ENSMUSG00000090996	Gm20458
ENSMUSG00000091003	Gm7244
ENSMUSG00000091020	Gm5828
ENSMUSG00000091105	Gm5633

ENSMUSG00000091119	Ccdc152
ENSMUSG00000091223	Gm8775
ENSMUSG00000091230	Gm6970
ENSMUSG00000091237	Gm17114
ENSMUSG00000091269	Gm6682
ENSMUSG00000091361	Gm5277
ENSMUSG00000091419	Gm17450
ENSMUSG00000091426	Gm17228
ENSMUSG00000091442	Gm17031
ENSMUSG00000091577	Gm6211
ENSMUSG00000091580	Gm6467
ENSMUSG00000091665	Gm17101
ENSMUSG00000091697	Eif3s6-ps2
ENSMUSG00000091705	H2-Q2
ENSMUSG00000091712	Sec14l5
ENSMUSG00000091721	Gm5549
ENSMUSG00000091732	Gm17541
ENSMUSG00000091803	Cox16
ENSMUSG00000091898	Tnnc1
ENSMUSG00000091942	Gm17138
ENSMUSG00000091957	Rps2-ps10
ENSMUSG00000091971	Hspa1a
ENSMUSG00000092014	Gm4468
ENSMUSG00000092054	Kif4-ps
ENSMUSG00000092060	Bend4
ENSMUSG00000092094	Zfp804b
ENSMUSG00000092116	Gm10320
ENSMUSG00000092171	4833427F10Rik
ENSMUSG00000092181	Gm20432
ENSMUSG00000092192	Dyx1c1
ENSMUSG00000092220	Gm20528
ENSMUSG00000092230	Gm18889
ENSMUSG00000092250	Gm20467
ENSMUSG00000092252	Gm20499
ENSMUSG00000092269	Gm9577
ENSMUSG00000092277	Gm19684
ENSMUSG00000092278	Gm8752
ENSMUSG00000092283	Gm20412
ENSMUSG00000092335	Gm7221
ENSMUSG00000092369	1700039E22Rik
ENSMUSG00000092394	Gm20445
ENSMUSG00000092438	Gm18734
ENSMUSG00000092447	Gm7696
ENSMUSG00000092469	Gm20395
ENSMUSG00000092474	Gm20478
ENSMUSG00000092499	1700092C10Rik
ENSMUSG00000092505	Gm19246
ENSMUSG00000092517	Art2a-ps
ENSMUSG00000092549	Gm20491
ENSMUSG00000092563	Gm3617
ENSMUSG00000092570	2210019I11Rik
ENSMUSG00000092624	Gm3654
ENSMUSG00000092655	Gm25238
ENSMUSG00000092746	Gm22179
ENSMUSG00000092953	Gm26217
ENSMUSG00000093075	Gm25948
ENSMUSG00000093080	Mir3060
ENSMUSG00000093283	Gm22567
ENSMUSG00000093315	Mir5114
ENSMUSG00000093346	Gm26115
ENSMUSG00000093392	Gm6061
ENSMUSG00000093406	Rpl48-ps1
ENSMUSG00000093543	Gm8473
ENSMUSG00000093552	Gm5777
ENSMUSG00000093581	Gm18006
ENSMUSG00000093587	Gm20554
ENSMUSG00000093589	Gm18367
ENSMUSG00000093645	Gm23187
ENSMUSG00000093650	Gm20631
ENSMUSG00000093661	Eif4e3
ENSMUSG00000093671	Gm20656
ENSMUSG00000093688	Gm8488

ENSMUSG00000093716	Gm19815
ENSMUSG00000093769	Hist2h3c1
ENSMUSG00000093806	Asmt
ENSMUSG00000093826	Gm6900
ENSMUSG00000093847	Gm5039
ENSMUSG00000093885	Rpl35a-ps5
ENSMUSG00000093954	Gm21464
ENSMUSG00000094015	Rps12-ps20
ENSMUSG00000094092	Gm15921
ENSMUSG00000094114	Gm21967
ENSMUSG00000094174	Ighv6-4
ENSMUSG00000094242	Gm5456
ENSMUSG00000094278	Gm12626
ENSMUSG00000094303	
ENSMUSG00000094320	Gm13202
ENSMUSG00000094338	Hist1h2bl
ENSMUSG00000094344	Gm11942
ENSMUSG00000094439	Gm21969
ENSMUSG00000094461	Olfr883
ENSMUSG00000094530	Gm21399
ENSMUSG00000094597	Gm4810
ENSMUSG00000094651	Gal3st2
ENSMUSG00000094664	Rpl35a-ps6
ENSMUSG00000094708	Gm10359
ENSMUSG00000094873	Gm13812
ENSMUSG00000094918	Gm8765
ENSMUSG00000094942	Gm3604
ENSMUSG00000094955	Gm3699
ENSMUSG00000094973	Gm8994
ENSMUSG00000095042	Gm12537
ENSMUSG00000095134	Gm21857
ENSMUSG00000095139	Pou3f2
ENSMUSG00000095150	Rps19-ps13
ENSMUSG00000095159	Tubb4b-ps1
ENSMUSG00000095203	Gm13810
ENSMUSG00000095224	F930015N05Rik
ENSMUSG00000095261	Gm17391
ENSMUSG00000095288	Gm7618
ENSMUSG00000095427	Rps2-ps6
ENSMUSG00000095440	Fignl2
ENSMUSG00000095457	Gm21911
ENSMUSG00000095478	Gm9824
ENSMUSG00000095512	Gm17222
ENSMUSG00000095526	Gm10243
ENSMUSG00000095562	Gm21887
ENSMUSG00000095614	Gm6291
ENSMUSG00000095649	Gm21884
ENSMUSG00000095908	Gm5576
ENSMUSG00000095937	Gm12671
ENSMUSG00000096051	Vmn1r40
ENSMUSG00000096066	Gm10424
ENSMUSG00000096154	Gm13057
ENSMUSG00000096157	Gm5472
ENSMUSG00000096177	Gm5070
ENSMUSG00000096233	Gm13238
ENSMUSG00000096258	Rps16-ps3
ENSMUSG00000096449	Gm4076
ENSMUSG00000096486	Gm5426
ENSMUSG00000096579	Gm17121
ENSMUSG00000096623	Gm8112
ENSMUSG00000096699	Rps19-ps4
ENSMUSG00000096712	Gm15454
ENSMUSG00000096726	Gm5558
ENSMUSG00000096727	Psmb9
ENSMUSG00000096768	Erdr1
ENSMUSG00000096842	Gm10736
ENSMUSG00000096937	RP23-91L18.2
ENSMUSG00000096942	Rps19-ps6
ENSMUSG00000096948	AC163347.1
ENSMUSG00000096957	AC124475.1
ENSMUSG00000097002	CT025649.1
ENSMUSG00000097028	AC114655.1

ENSMUSG00000097041	RP23-404J7.2
ENSMUSG00000097122	AL731663.1
ENSMUSG00000097142	AC102815.1
ENSMUSG00000097216	AC119206.1
ENSMUSG00000097224	RP23-412B2.6
ENSMUSG00000097250	AC141473.1
ENSMUSG00000097255	Rpl31-ps7
ENSMUSG00000097336	RP23-155H5.2
ENSMUSG00000097354	AC142191.1
ENSMUSG00000097421	D630011A20Rik
ENSMUSG00000097423	AC124322.1
ENSMUSG00000097426	RP24-245K18.1
ENSMUSG00000097493	AC164597.1
ENSMUSG00000097529	Rpl31-ps19
ENSMUSG00000097535	AC159314.1
ENSMUSG00000097679	Rps19-ps5
ENSMUSG00000097703	AC055772.1
ENSMUSG00000097754	RP24-160J12.1
ENSMUSG00000097764	AC110573.1
ENSMUSG00000097788	AC154910.1
ENSMUSG00000097789	AC129605.1
ENSMUSG00000097861	CT033751.2
ENSMUSG00000097919	RP24-414A22.6
ENSMUSG00000097950	RP23-103N6.1
ENSMUSG00000097968	RP23-1B19.3
ENSMUSG00000098000	RP23-2B18.1
ENSMUSG00000098007	RP23-146P1.1
ENSMUSG00000098053	RP24-510G5.4
ENSMUSG00000098064	RP23-419L21.1
ENSMUSG00000098073	RP24-204J10.1
ENSMUSG00000098083	RP24-251F16.3
ENSMUSG00000098110	RP23-23I2.1
ENSMUSG00000098126	RP24-333N14.1
ENSMUSG00000098136	RP23-285H4.1
ENSMUSG00000098138	RP23-136O17.1
ENSMUSG00000098167	RP24-426K19.1
ENSMUSG00000098169	RP23-347H20.1
ENSMUSG00000098177	RP24-352J7.1
ENSMUSG00000098178	RP23-81C12.3
ENSMUSG00000098179	RP23-138N1.1
ENSMUSG00000098198	RP24-290G2.1
ENSMUSG00000098201	RP24-149G24.1
ENSMUSG00000098205	RP23-337B16.1
ENSMUSG00000098222	RP23-269P8.1
ENSMUSG00000098265	
ENSMUSG00000098275	
ENSMUSG00000098276	
ENSMUSG00000098281	
ENSMUSG00000098293	
ENSMUSG00000098524	
ENSMUSG00000098543	
ENSMUSG00000098557	
ENSMUSG00000098641	
ENSMUSG00000098688	
ENSMUSG00000098743	
ENSMUSG00000098761	
ENSMUSG00000098798	
ENSMUSG00000098837	
ENSMUSG00000098842	
ENSMUSG00000098925	
ENSMUSG00000098948	
ENSMUSG00000098992	
ENSMUSG00000099101	
ENSMUSG00000099125	
ENSMUSG00000099253	
ENSMUSG00000099312	
ENSMUSG00000099325	
ENSMUSG00000099373	
ENSMUSG00000099377	
ENSMUSG00000099417	
ENSMUSG00000099423	
ENSMUSG00000099433	

ENSMUSG00000099470
ENSMUSG00000099492
ENSMUSG00000099521
ENSMUSG00000099627
ENSMUSG00000099701
ENSMUSG00000099714
ENSMUSG00000099732
ENSMUSG00000099741
ENSMUSG00000099757
ENSMUSG00000099816
ENSMUSG00000099858
ENSMUSG00000099860
ENSMUSG00000099870
ENSMUSG00000099973
ENSMUSG00000100025
ENSMUSG00000100033
ENSMUSG00000100037
ENSMUSG00000100078
ENSMUSG00000100090
ENSMUSG00000100160
ENSMUSG00000100178
ENSMUSG00000100204
ENSMUSG00000100215
ENSMUSG00000100218
ENSMUSG00000100319
ENSMUSG00000100335
ENSMUSG00000100393
ENSMUSG00000100432
ENSMUSG00000100434
ENSMUSG00000100441
ENSMUSG00000100536
ENSMUSG00000100561
ENSMUSG00000100592
ENSMUSG00000100615
ENSMUSG00000100619
ENSMUSG00000100621
ENSMUSG00000100696
ENSMUSG00000100700
ENSMUSG00000100701
ENSMUSG00000100702
ENSMUSG00000100706
ENSMUSG00000100767
ENSMUSG00000100839
ENSMUSG00000100927
ENSMUSG00000100934
ENSMUSG00000100968
ENSMUSG00000100980
ENSMUSG00000100992
ENSMUSG00000101129
ENSMUSG00000101206
ENSMUSG00000101236
ENSMUSG00000101262
ENSMUSG00000101301
ENSMUSG00000101337
ENSMUSG00000101355
ENSMUSG00000101523
ENSMUSG00000101574
ENSMUSG00000101610
ENSMUSG00000101666
ENSMUSG00000101682
ENSMUSG00000101685
ENSMUSG00000101730
ENSMUSG00000101784
ENSMUSG00000101795
ENSMUSG00000101800
ENSMUSG00000101803
ENSMUSG00000101939
ENSMUSG00000101958
ENSMUSG00000101966
ENSMUSG00000102075
ENSMUSG00000102083
ENSMUSG00000102095

ENSMUSG00000102162
ENSMUSG00000102193
ENSMUSG00000102219
ENSMUSG00000102252
ENSMUSG00000102475
ENSMUSG00000102478
ENSMUSG00000102490
ENSMUSG00000102493
ENSMUSG00000102503
ENSMUSG00000102516
ENSMUSG00000102577
ENSMUSG00000102609
ENSMUSG00000102615
ENSMUSG00000102627
ENSMUSG00000102637
ENSMUSG00000102692
ENSMUSG00000102708
ENSMUSG00000102752
ENSMUSG00000102813
ENSMUSG00000102858
ENSMUSG00000102882
ENSMUSG00000102908
ENSMUSG00000102915
ENSMUSG00000102931
ENSMUSG00000102943
ENSMUSG00000102950
ENSMUSG00000102972
ENSMUSG00000103045
ENSMUSG00000103057
ENSMUSG00000103114
ENSMUSG00000103129
ENSMUSG00000103152
ENSMUSG00000103173
ENSMUSG00000103208
ENSMUSG00000103235
ENSMUSG00000103278
ENSMUSG00000103286
ENSMUSG00000103312
ENSMUSG00000103332
ENSMUSG00000103351
ENSMUSG00000103367
ENSMUSG00000103380
ENSMUSG00000103423
ENSMUSG00000103433
ENSMUSG00000103501
ENSMUSG00000103534
ENSMUSG00000103571
ENSMUSG00000103574
ENSMUSG00000103614
ENSMUSG00000103643
ENSMUSG00000103661
ENSMUSG00000103665
ENSMUSG00000103792
ENSMUSG00000103801
ENSMUSG00000103888
ENSMUSG00000103921
ENSMUSG00000103922
ENSMUSG00000103986
ENSMUSG00000104043
ENSMUSG00000104076
ENSMUSG00000104126
ENSMUSG00000104166
ENSMUSG00000104171
ENSMUSG00000104182
ENSMUSG00000104183
ENSMUSG00000104236
ENSMUSG00000104274
ENSMUSG00000104298
ENSMUSG00000104360
ENSMUSG00000104385
ENSMUSG00000104390
