# Supplementary material for: Computational Identification of Key Regulators in Two Different Colorectal Cancer Cell Lines
Source: Front Genet. 2016 Apr 5;7:42. doi: 10.3389/fgene.2016.00042 (PMC4820448; doi:10.3389/fgene.2016.00042)
Supplement: Supplementary Table S2 — Signature genes for colorectal cell line CMT-93. [file Table2.PDF]

Table S1. The Trinity platform was first used to perform a differentially expressed gene (DEG) analysis based on RNA-seq data, which included three biological replicates for 1638N-T1 and CMT-93, respectively. In the second step, the genes were identified, which are most significantly upregulated in CMT-93 and, at the same time, downregulated in 1638N-T1. This category contains the signature genes for the cell line CMT-93.

ID	Gene symbol
ENSMUSG00000000031	H19
ENSMUSG00000000197	Nalcn
ENSMUSG00000000204	Slfn4
ENSMUSG00000000244	Tspan32
ENSMUSG00000000303	Cdh1
ENSMUSG00000000385	Tmprss2
ENSMUSG00000000386	Mx1
ENSMUSG00000000409	Lck
ENSMUSG00000000416	Cttnbp2
ENSMUSG00000000544	Gpa33
ENSMUSG00000000686	Abhd15
ENSMUSG00000000690	Hoxb6
ENSMUSG00000000706	Btn1a1
ENSMUSG00000000732	Icosl
ENSMUSG00000000792	Slc5a5
ENSMUSG00000001014	Icam4
ENSMUSG00000001020	S100a4
ENSMUSG00000001023	S100a5
ENSMUSG00000001249	Hpn
ENSMUSG00000001334	Fndc5
ENSMUSG00000001348	Acp5
ENSMUSG00000001420	Tmem79
ENSMUSG00000001510	Dlx3
ENSMUSG00000001520	Nrip2
ENSMUSG00000001583	Tnk1
ENSMUSG00000001588	Acap1
ENSMUSG00000001604	Tcea3
ENSMUSG00000001656	Hoxc11
ENSMUSG00000001657	Hoxc8
ENSMUSG00000001661	Hoxc6
ENSMUSG00000001672	Marveld3
ENSMUSG00000001819	Hoxd13
ENSMUSG00000001946	Esam
ENSMUSG00000002007	Srpk3
ENSMUSG00000002100	Mybpc3
ENSMUSG00000002108	Nr1h3
ENSMUSG00000002111	Sfpi1
ENSMUSG00000002228	Ppm1j
ENSMUSG00000002257	Def6
ENSMUSG00000002289	Angptl4
ENSMUSG00000002459	Rgs20
ENSMUSG00000002475	Abhd3
ENSMUSG00000002578	Ikzf4
ENSMUSG00000002602	Axl
ENSMUSG00000002769	Gnmt
ENSMUSG00000003051	Elf3
ENSMUSG00000003206	Ebi3
ENSMUSG00000003279	Dlgap1
ENSMUSG00000003282	Plag1
ENSMUSG00000003309	Ap1m2
ENSMUSG00000003355	Fkbp11
ENSMUSG00000003378	Grik5
ENSMUSG00000003411	Rab3b
ENSMUSG00000003824	Syce2
ENSMUSG00000003849	Nqo1
ENSMUSG00000003949	Hlf
ENSMUSG00000004035	Gstm7
ENSMUSG00000004113	Cacna1b
ENSMUSG00000004266	Ptpn6
ENSMUSG00000004328	Hif3a
ENSMUSG00000004371	Il11
ENSMUSG00000004473	Clec11a
ENSMUSG00000004558	Ndrg2
ENSMUSG00000004654	Ghrhr
ENSMUSG00000004655	Aqp1

ENSMUSG00000004698	Hdac9
ENSMUSG00000004864	Mapk13
ENSMUSG00000004872	Pax3
ENSMUSG00000004885	Crabp2
ENSMUSG00000004891	Nes
ENSMUSG00000004951	Hspb1
ENSMUSG00000005397	Nid1
ENSMUSG00000005469	Prkaca
ENSMUSG00000005470	Asf1b
ENSMUSG00000005628	Tmod4
ENSMUSG00000005672	Kit
ENSMUSG00000005705	Agrp
ENSMUSG00000005950	P2rx5
ENSMUSG00000005951	Shpk
ENSMUSG00000005952	Trpv1
ENSMUSG00000005973	Rcn1
ENSMUSG00000006056	Calcoco2
ENSMUSG00000006143	Upk3bl
ENSMUSG00000006179	Prss16
ENSMUSG00000006310	Zbtb32
ENSMUSG00000006345	Ggt1
ENSMUSG00000006360	Crip1
ENSMUSG00000006362	Cbfa2t3
ENSMUSG00000006386	Tek
ENSMUSG00000006411	Pvrl4
ENSMUSG00000006464	Bbs1
ENSMUSG00000006587	Snai3
ENSMUSG00000006777	Krt23
ENSMUSG00000007021	Syngr3
ENSMUSG00000007034	Slc44a4
ENSMUSG00000007035	Msh5
ENSMUSG00000007039	Ddah2
ENSMUSG00000007457	2310003L06Rik
ENSMUSG00000007655	Cav1
ENSMUSG00000007682	Dio2
ENSMUSG00000007783	Cpt1c
ENSMUSG00000008028	1700008O03Rik
ENSMUSG00000008129	4930432K21Rik
ENSMUSG00000008153	Clstn3
ENSMUSG00000008206	Cers4
ENSMUSG00000008461	Fut1
ENSMUSG00000009092	Derl3
ENSMUSG00000009097	Tbx1
ENSMUSG00000009108	Gnat2
ENSMUSG00000009185	Ccl8
ENSMUSG00000009487	Otog
ENSMUSG00000009596	Taf7l
ENSMUSG00000009628	Tex15
ENSMUSG00000010044	Zmynd10
ENSMUSG00000010311	Optc
ENSMUSG00000010342	Tex14
ENSMUSG00000010476	Ebf3
ENSMUSG00000010505	Myt1
ENSMUSG00000010601	Apol7a
ENSMUSG00000010797	Wnt2
ENSMUSG00000011052	1700049J03Rik
ENSMUSG00000011486	Slc25a41
ENSMUSG00000012017	Scarf2
ENSMUSG00000012187	Mogat1
ENSMUSG00000012428	Steap4
ENSMUSG00000013091	Tmem190
ENSMUSG00000013418	B4galnt2
ENSMUSG00000013483	Card14
ENSMUSG00000013766	Ly6g6e
ENSMUSG00000013921	Clip3
ENSMUSG00000014158	Trpv4
ENSMUSG00000014198	Zfp385c
ENSMUSG00000014361	Mertk
ENSMUSG00000014453	Blk
ENSMUSG00000014776	Nol3
ENSMUSG00000014782	Plekhg4
ENSMUSG00000014791	Elmo3

ENSMUSG00000015090	Ptgds
ENSMUSG00000015093	Clic3
ENSMUSG00000015134	Aldh1a3
ENSMUSG00000015189	Casd1
ENSMUSG00000015222	Map2
ENSMUSG00000015468	Notch4
ENSMUSG00000015568	Lpl
ENSMUSG00000015702	Anxa9
ENSMUSG00000015962	1700016C15Rik
ENSMUSG00000016356	Col20a1
ENSMUSG00000016498	Pdcd1lg2
ENSMUSG00000016552	Foxred2
ENSMUSG00000016918	Sulf1
ENSMUSG00000016982	Pom121l2
ENSMUSG00000016995	Matn4
ENSMUSG00000017007	Rbpjl
ENSMUSG00000017204	Gsdma
ENSMUSG00000017390	Aldoc
ENSMUSG00000017400	Stac2
ENSMUSG00000017493	Igfbp4
ENSMUSG00000017667	Zfp334
ENSMUSG00000017723	Wfdc2
ENSMUSG00000017767	Spata25
ENSMUSG00000017868	Sgk2
ENSMUSG00000017950	Hnf4a
ENSMUSG00000018166	Erbb3
ENSMUSG00000018479	1700125H20Rik
ENSMUSG00000018566	Slc2a4
ENSMUSG00000018569	Cldn7
ENSMUSG00000018656	Fam115e
ENSMUSG00000018698	Lhx1
ENSMUSG00000018752	BC096441
ENSMUSG00000018845	Unc45b
ENSMUSG00000018920	Cxcl16
ENSMUSG00000018983	E2f2
ENSMUSG00000018986	Slfn3
ENSMUSG00000019124	Scrn1
ENSMUSG00000019230	Lhx9
ENSMUSG00000019235	Rps6kl1
ENSMUSG00000019278	Dpep1
ENSMUSG00000019539	Rcn3
ENSMUSG00000019577	Pdk4
ENSMUSG00000019732	Calr3
ENSMUSG00000019734	Tmc4
ENSMUSG00000019737	Syne4
ENSMUSG00000019817	Plagl1
ENSMUSG00000019852	D10Bwg1379e
ENSMUSG00000019888	Mgat4c
ENSMUSG00000019890	Nts
ENSMUSG00000019933	2310015B20Rik
ENSMUSG00000019987	Arg1
ENSMUSG00000019990	Pde7b
ENSMUSG00000020023	Tmcc3
ENSMUSG00000020042	Btbd11
ENSMUSG00000020044	Timp3
ENSMUSG00000020120	Plek
ENSMUSG00000020135	Apc2
ENSMUSG00000020154	Ptprb
ENSMUSG00000020169	Best3
ENSMUSG00000020173	Cobl
ENSMUSG00000020227	Irak3
ENSMUSG00000020256	Aldh1l2
ENSMUSG00000020297	Nsg2
ENSMUSG00000020424	Gatsl3
ENSMUSG00000020435	Osbp2
ENSMUSG00000020491	2810021J22Rik
ENSMUSG00000020566	Atp6v1c2
ENSMUSG00000020577	Tspan13
ENSMUSG00000020581	Agr2
ENSMUSG00000020607	Fam84a
ENSMUSG00000020609	Apob
ENSMUSG00000020633	Dcdc2c

ENSMUSG00000020646	Mboat2
ENSMUSG00000020656	Grhl1
ENSMUSG00000020758	Itgb4
ENSMUSG00000020773	Trim47
ENSMUSG00000020774	Aspa
ENSMUSG00000020788	Atp2a3
ENSMUSG00000020805	Slc13a5
ENSMUSG00000020871	Dlx4
ENSMUSG00000020882	Cacnb1
ENSMUSG00000020892	Aloxe3
ENSMUSG00000020990	Cdkl1
ENSMUSG00000021055	Esr2
ENSMUSG00000021057	Akap5
ENSMUSG00000021062	Rab15
ENSMUSG00000021101	4930408O17Rik
ENSMUSG00000021136	Smoc1
ENSMUSG00000021211	Akr1c12
ENSMUSG00000021223	Papln
ENSMUSG00000021245	Mlh3
ENSMUSG00000021250	Fos
ENSMUSG00000021255	Esrrb
ENSMUSG00000021298	Gpr132
ENSMUSG00000021301	Hecw1
ENSMUSG00000021319	Sfrp4
ENSMUSG00000021384	Susd3
ENSMUSG00000021403	Serpinb9b
ENSMUSG00000021416	Eci3
ENSMUSG00000021451	Sema4d
ENSMUSG00000021456	Fbp2
ENSMUSG00000021509	Slc25a48
ENSMUSG00000021575	Ahrr
ENSMUSG00000021596	Mctp1
ENSMUSG00000021614	Vcan
ENSMUSG00000021636	Marveld2
ENSMUSG00000021638	Ocln
ENSMUSG00000021678	F2rl1
ENSMUSG00000021679	S100z
ENSMUSG00000021680	Crhbp
ENSMUSG00000021684	Pde8b
ENSMUSG00000021749	Oit1
ENSMUSG00000021822	Plau
ENSMUSG00000021879	Dnahc12
ENSMUSG00000021966	Prss52
ENSMUSG00000021974	Fgf9
ENSMUSG00000021990	Spata13
ENSMUSG00000022014	Epsti1
ENSMUSG00000022090	Pdlim2
ENSMUSG00000022102	Dok2
ENSMUSG00000022199	Slc22a17
ENSMUSG00000022203	Efs
ENSMUSG00000022218	Tgm1
ENSMUSG00000022221	Ripk3
ENSMUSG00000022225	Cma1
ENSMUSG00000022227	Mcpt1
ENSMUSG00000022231	Sema5a
ENSMUSG00000022262	Dnahc5
ENSMUSG00000022306	Zfpm2
ENSMUSG00000022340	Sybu
ENSMUSG00000022372	Sla
ENSMUSG00000022376	Adcy8
ENSMUSG00000022382	Wnt7b
ENSMUSG00000022469	Rapgef3
ENSMUSG00000022479	Vdr
ENSMUSG00000022484	Hoxc10
ENSMUSG00000022485	Hoxc5
ENSMUSG00000022487	Gtsf1
ENSMUSG00000022488	Nckap1l
ENSMUSG00000022514	Il1rap
ENSMUSG00000022548	Apod
ENSMUSG00000022574	Naprt1
ENSMUSG00000022582	Ly6g
ENSMUSG00000022595	Lypd2

ENSMUSG00000022596	Slurp1
ENSMUSG00000022598	Psca
ENSMUSG00000022603	Mroh4
ENSMUSG00000022656	Pvrl3
ENSMUSG00000022747	St3gal6
ENSMUSG00000022750	Klhl22
ENSMUSG00000022763	Aifm3
ENSMUSG00000022803	Popdc2
ENSMUSG00000022816	Fstl1
ENSMUSG00000022847	Thpo
ENSMUSG00000022860	Chodl
ENSMUSG00000022899	Slc15a2
ENSMUSG00000022900	Ildr1
ENSMUSG00000022941	Ripply3
ENSMUSG00000023000	Dhh
ENSMUSG00000023011	Faim2
ENSMUSG00000023013	Aqp2
ENSMUSG00000023034	Nr4a1
ENSMUSG00000023039	Krt7
ENSMUSG00000023045	Soat2
ENSMUSG00000023046	Igfbp6
ENSMUSG00000023057	Fabp2
ENSMUSG00000023064	Sncg
ENSMUSG00000023078	Cxcl13
ENSMUSG00000023328	Ache
ENSMUSG00000023336	Wfdc1
ENSMUSG00000023393	Slc17a9
ENSMUSG00000023467	Tulp2
ENSMUSG00000023868	Pde10a
ENSMUSG00000023873	1700010I14Rik
ENSMUSG00000023902	Zscan10
ENSMUSG00000023906	Cldn6
ENSMUSG00000023931	Efhb
ENSMUSG00000023959	Clic5
ENSMUSG00000023966	Rsph9
ENSMUSG00000024215	Spdef
ENSMUSG00000024268	Celf4
ENSMUSG00000024274	Zscan30
ENSMUSG00000024302	Dtna
ENSMUSG00000024349	Tmem173
ENSMUSG00000024365	Cyp21a1
ENSMUSG00000024371	C2
ENSMUSG00000024388	Myo7b
ENSMUSG00000024401	Tnf
ENSMUSG00000024421	Lama3
ENSMUSG00000024448	H2-M10.1
ENSMUSG00000024512	Dynap
ENSMUSG00000024530	Slmo1
ENSMUSG00000024565	Sall3
ENSMUSG00000024653	Scgb1a1
ENSMUSG00000024697	Gna14
ENSMUSG00000024727	Trpm6
ENSMUSG00000024747	Aldh1a7
ENSMUSG00000024771	Lipk
ENSMUSG00000024774	Ankrd22
ENSMUSG00000024810	Il33
ENSMUSG00000024842	Cabp4
ENSMUSG00000024846	Cst6
ENSMUSG00000024854	Pold4
ENSMUSG00000024855	Pacs1
ENSMUSG00000024909	Efemp2
ENSMUSG00000024912	Fosl1
ENSMUSG00000024922	Ovol1
ENSMUSG00000024936	Kcnk7
ENSMUSG00000024972	Lgals12
ENSMUSG00000025002	Cyp2c55
ENSMUSG00000025037	Maoa
ENSMUSG00000025064	Col17a1
ENSMUSG00000025092	Hspa12a
ENSMUSG00000025105	Bnc1
ENSMUSG00000025129	Ppp1r27
ENSMUSG00000025161	Slc16a3

ENSMUSG00000025165	Sectm1a
ENSMUSG00000025202	Scd3
ENSMUSG00000025279	Dnase1l3
ENSMUSG00000025330	Padi4
ENSMUSG00000025370	Cdh9
ENSMUSG00000025375	Aatk
ENSMUSG00000025383	Il23a
ENSMUSG00000025429	Pstpip2
ENSMUSG00000025432	Avil
ENSMUSG00000025481	1190003J15Rik
ENSMUSG00000025491	Ifitm1
ENSMUSG00000025492	Ifitm3
ENSMUSG00000025496	Drd4
ENSMUSG00000025497	Cdhr5
ENSMUSG00000025500	1600016N20Rik
ENSMUSG00000025504	Eps8l2
ENSMUSG00000025515	Muc2
ENSMUSG00000025584	Pde8a
ENSMUSG00000025650	Col7a1
ENSMUSG00000025656	Arhgef9
ENSMUSG00000025665	Rps6ka6
ENSMUSG00000025739	Gng13
ENSMUSG00000025757	Hspa4l
ENSMUSG00000025813	Homer2
ENSMUSG00000025815	Dhtkd1
ENSMUSG00000025855	Prkar1b
ENSMUSG00000025888	Casp1
ENSMUSG00000025902	Sox17
ENSMUSG00000025932	Eya1
ENSMUSG00000026065	Slc9a4
ENSMUSG00000026090	2010300C02Rik
ENSMUSG00000026110	Mgat4a
ENSMUSG00000026114	Cnga3
ENSMUSG00000026126	Ptpn18
ENSMUSG00000026170	Cyp27a1
ENSMUSG00000026175	Vil1
ENSMUSG00000026205	Slc23a3
ENSMUSG00000026211	Obsl1
ENSMUSG00000026253	Chrng
ENSMUSG00000026348	Acmsd
ENSMUSG00000026354	Lct
ENSMUSG00000026395	Ptprc
ENSMUSG00000026399	Cd55
ENSMUSG00000026413	Pkp1
ENSMUSG00000026420	Il24
ENSMUSG00000026450	Chit1
ENSMUSG00000026475	Rgs16
ENSMUSG00000026479	Lamc2
ENSMUSG00000026535	Ifi202b
ENSMUSG00000026564	Dusp27
ENSMUSG00000026589	Sec16b
ENSMUSG00000026602	Nphs2
ENSMUSG00000026616	Cr2
ENSMUSG00000026638	Irf6
ENSMUSG00000026639	Lamb3
ENSMUSG00000026674	Ddr2
ENSMUSG00000026692	Fmo4
ENSMUSG00000026764	Kif5c
ENSMUSG00000026765	Lypd6b
ENSMUSG00000026768	Itga8
ENSMUSG00000026822	Lcn2
ENSMUSG00000026826	Nr4a2
ENSMUSG00000026840	Lamc3
ENSMUSG00000026841	Fibcd1
ENSMUSG00000026870	D730039F16Rik
ENSMUSG00000026959	Grin1
ENSMUSG00000026961	Lrrc26
ENSMUSG00000026969	Fam166a
ENSMUSG00000026976	Pax8
ENSMUSG00000027048	Abcb11
ENSMUSG00000027068	Dhrs9
ENSMUSG00000027070	Lrp2

ENSMUSG00000027071	P2rx3
ENSMUSG00000027074	Slc43a3
ENSMUSG00000027075	Slc43a1
ENSMUSG00000027082	Tfpi
ENSMUSG00000027168	Pax6
ENSMUSG00000027199	Gatm
ENSMUSG00000027204	Fbn1
ENSMUSG00000027208	Fgf7
ENSMUSG00000027239	Mdk
ENSMUSG00000027246	Ell3
ENSMUSG00000027273	Snap25
ENSMUSG00000027315	Spint1
ENSMUSG00000027338	Prnd
ENSMUSG00000027356	Fermt1
ENSMUSG00000027375	Mal
ENSMUSG00000027377	Mall
ENSMUSG00000027380	Acoxl
ENSMUSG00000027386	Fbln7
ENSMUSG00000027412	Lpin3
ENSMUSG00000027420	Bfsp1
ENSMUSG00000027435	Cd93
ENSMUSG00000027456	Sdcbp2
ENSMUSG00000027533	Fabp5
ENSMUSG00000027544	Nfatc2
ENSMUSG00000027559	Car3
ENSMUSG00000027562	Car2
ENSMUSG00000027574	Nkain4
ENSMUSG00000027611	Procr
ENSMUSG00000027669	Gnb4
ENSMUSG00000027784	Ppm1l
ENSMUSG00000027796	Smad9
ENSMUSG00000027834	Serpini1
ENSMUSG00000027858	Tspan2
ENSMUSG00000027859	Ngf
ENSMUSG00000027861	Casq2
ENSMUSG00000027868	Tbx15
ENSMUSG00000027890	Gstm4
ENSMUSG00000027901	Dennd2d
ENSMUSG00000027913	Crct1
ENSMUSG00000027919	Lce1g
ENSMUSG00000027938	Creb3l4
ENSMUSG00000027939	Nup210l
ENSMUSG00000027950	Chrnb2
ENSMUSG00000027978	Prss12
ENSMUSG00000028009	1700061I17Rik
ENSMUSG00000028031	Dkk2
ENSMUSG00000028037	Ifi44
ENSMUSG00000028064	Sema4a
ENSMUSG00000028072	Ntrk1
ENSMUSG00000028076	Cd1d1
ENSMUSG00000028088	Fmo5
ENSMUSG00000028125	Abca4
ENSMUSG00000028128	F3
ENSMUSG00000028158	Mttp
ENSMUSG00000028167	Bdh2
ENSMUSG00000028179	Cth
ENSMUSG00000028182	Lrriq3
ENSMUSG00000028217	Cdh17
ENSMUSG00000028223	Decr1
ENSMUSG00000028247	Coq3
ENSMUSG00000028341	Nr4a3
ENSMUSG00000028357	Kif12
ENSMUSG00000028364	Tnc
ENSMUSG00000028381	Ugcg
ENSMUSG00000028427	Aqp7
ENSMUSG00000028434	Epb4.1l4b
ENSMUSG00000028435	Aqp3
ENSMUSG00000028457	Atp8b5
ENSMUSG00000028464	Tpm2
ENSMUSG00000028469	Npr2
ENSMUSG00000028494	Plin2
ENSMUSG00000028524	Sgip1

ENSMUSG00000028525	Pde4b
ENSMUSG00000028527	Ak4
ENSMUSG00000028539	Artn
ENSMUSG00000028541	B4galt2
ENSMUSG00000028542	Slc6a9
ENSMUSG00000028575	Eqtn
ENSMUSG00000028581	Laptm5
ENSMUSG00000028629	Exo5
ENSMUSG00000028635	Edn2
ENSMUSG00000028644	Ermap
ENSMUSG00000028655	Mfsd2a
ENSMUSG00000028664	Ephb2
ENSMUSG00000028699	Tspan1
ENSMUSG00000028701	Lurap1
ENSMUSG00000028717	Tal1
ENSMUSG00000028738	Tas1r2
ENSMUSG00000028751	Pla2g2e
ENSMUSG00000028778	Hcrtr1
ENSMUSG00000028794	A3galt2
ENSMUSG00000028841	Cnksr1
ENSMUSG00000028883	Sema3a
ENSMUSG00000028972	Car6
ENSMUSG00000028976	Slc2a5
ENSMUSG00000028978	Nos3
ENSMUSG00000029019	Nppb
ENSMUSG00000029032	Arhgef16
ENSMUSG00000029053	Prkcz
ENSMUSG00000029061	Mmp23
ENSMUSG00000029074	Ttll10
ENSMUSG00000029086	Prom1
ENSMUSG00000029121	Crmp1
ENSMUSG00000029123	Stk32b
ENSMUSG00000029134	Plb1
ENSMUSG00000029149	Krtcap3
ENSMUSG00000029161	Cgref1
ENSMUSG00000029188	Slc34a2
ENSMUSG00000029195	Klb
ENSMUSG00000029255	Gnrhr
ENSMUSG00000029269	Sult1b1
ENSMUSG00000029275	Gfi1
ENSMUSG00000029309	Sparcl1
ENSMUSG00000029322	Plac8
ENSMUSG00000029330	Cds1
ENSMUSG00000029335	Bmp3
ENSMUSG00000029343	Crybb1
ENSMUSG00000029352	Crybb3
ENSMUSG00000029378	Areg
ENSMUSG00000029379	Cxcl3
ENSMUSG00000029384	2010109A12Rik
ENSMUSG00000029403	Cdkl2
ENSMUSG00000029449	Rhof
ENSMUSG00000029477	Morn3
ENSMUSG00000029490	Mfsd7a
ENSMUSG00000029556	Hnf1a
ENSMUSG00000029581	Fscn1
ENSMUSG00000029651	Mtus2
ENSMUSG00000029658	Wdr95
ENSMUSG00000029716	Tfr2
ENSMUSG00000029727	Cyp3a13
ENSMUSG00000029757	Dync1i1
ENSMUSG00000029784	Ssmem1
ENSMUSG00000029797	Sspo
ENSMUSG00000029814	Igf2bp3
ENSMUSG00000029828	4921507P07Rik
ENSMUSG00000029838	Ptn
ENSMUSG00000029847	2010107G12Rik
ENSMUSG00000029859	Epha1
ENSMUSG00000029916	Agk
ENSMUSG00000030050	Gkn1
ENSMUSG00000030064	Frmd4b
ENSMUSG00000030074	Gxylt2
ENSMUSG00000030088	Aldh1l1

ENSMUSG00000030093	Wnt7a
ENSMUSG00000030098	Grip2
ENSMUSG00000030107	Usp18
ENSMUSG00000030108	Slc6a13
ENSMUSG00000030109	Slc6a12
ENSMUSG00000030110	Ret
ENSMUSG00000030137	Tuba8
ENSMUSG00000030162	Olr1
ENSMUSG00000030165	Klrd1
ENSMUSG00000030167	Klrc1
ENSMUSG00000030187	Klra2
ENSMUSG00000030206	Gsg1
ENSMUSG00000030208	Emp1
ENSMUSG00000030255	Sspn
ENSMUSG00000030256	Bhlhe41
ENSMUSG00000030302	Atp2b2
ENSMUSG00000030319	Cand2
ENSMUSG00000030359	Pzp
ENSMUSG00000030413	Pglyrp1
ENSMUSG00000030428	Ttyh1
ENSMUSG00000030483	Cyp2b10
ENSMUSG00000030486	Zfp108
ENSMUSG00000030491	Tdrd12
ENSMUSG00000030494	Rhpn2
ENSMUSG00000030500	Slc17a6
ENSMUSG00000030518	Fam189a1
ENSMUSG00000030523	Trpm1
ENSMUSG00000030598	Fbxo17
ENSMUSG00000030623	1700019G06Rik
ENSMUSG00000030630	Fah
ENSMUSG00000030670	Cyp2r1
ENSMUSG00000030671	Pde3b
ENSMUSG00000030693	Klk10
ENSMUSG00000030717	Nupr1
ENSMUSG00000030739	Myh14
ENSMUSG00000030762	Aqp8
ENSMUSG00000030782	Tgfb1i1
ENSMUSG00000030785	Cox6a2
ENSMUSG00000030790	Adm
ENSMUSG00000030800	Prss8
ENSMUSG00000030806	Stx1b
ENSMUSG00000030825	Hsd17b14
ENSMUSG00000030877	4933427G17Rik
ENSMUSG00000030909	Anks4b
ENSMUSG00000031066	Usp11
ENSMUSG00000031089	Slc6a14
ENSMUSG00000031101	Sash3
ENSMUSG00000031104	Rab33a
ENSMUSG00000031125	3830403N18Rik
ENSMUSG00000031129	Slc9a9
ENSMUSG00000031147	Magix
ENSMUSG00000031209	Heph
ENSMUSG00000031286	Glt28d2
ENSMUSG00000031293	Rs1
ENSMUSG00000031294	D630029K05Rik
ENSMUSG00000031351	Zfp185
ENSMUSG00000031362	Xlr4c
ENSMUSG00000031387	Renbp
ENSMUSG00000031389	Arhgap4
ENSMUSG00000031397	Tktl1
ENSMUSG00000031410	Nxf7
ENSMUSG00000031448	Adprhl1
ENSMUSG00000031497	Tnfsf13b
ENSMUSG00000031502	Col4a1
ENSMUSG00000031503	Col4a2
ENSMUSG00000031519	Asb5
ENSMUSG00000031553	Adam3
ENSMUSG00000031613	Hpgd
ENSMUSG00000031635	Anxa10
ENSMUSG00000031647	Mfap3l
ENSMUSG00000031710	Ucp1
ENSMUSG00000031712	Il15

ENSMUSG00000031758	Cdyl2
ENSMUSG00000031770	Herpud1
ENSMUSG00000031775	Pllp
ENSMUSG00000031790	Mmp15
ENSMUSG00000031837	Necab2
ENSMUSG00000031841	Cdh13
ENSMUSG00000031849	Comp
ENSMUSG00000031875	Cmtm3
ENSMUSG00000031981	Capn9
ENSMUSG00000031995	St14
ENSMUSG00000032021	Crtam
ENSMUSG00000032024	Clmp
ENSMUSG00000032036	Kirrel3
ENSMUSG00000032066	Bco2
ENSMUSG00000032068	1600029D21Rik
ENSMUSG00000032079	Apoa5
ENSMUSG00000032083	Apoa1
ENSMUSG00000032092	Mpzl2
ENSMUSG00000032101	Ddx25
ENSMUSG00000032134	Muc16
ENSMUSG00000032192	Gnb5
ENSMUSG00000032202	Rab27a
ENSMUSG00000032226	Gcnt3
ENSMUSG00000032246	Calml4
ENSMUSG00000032262	Elovl4
ENSMUSG00000032265	Fam46a
ENSMUSG00000032315	Cyp1a1
ENSMUSG00000032323	Cyp11a1
ENSMUSG00000032358	Fam83b
ENSMUSG00000032377	Plscr4
ENSMUSG00000032380	Dapk2
ENSMUSG00000032436	Cmtm7
ENSMUSG00000032446	Eomes
ENSMUSG00000032548	Slco2a1
ENSMUSG00000032561	Acpp
ENSMUSG00000032589	Bsn
ENSMUSG00000032591	Mst1
ENSMUSG00000032607	Amt
ENSMUSG00000032657	Fam189b
ENSMUSG00000032715	Trib3
ENSMUSG00000032719	Sbspon
ENSMUSG00000032766	Gng11
ENSMUSG00000032875	Arhgef17
ENSMUSG00000032899	Styk1
ENSMUSG00000032908	Sgpp2
ENSMUSG00000032968	Inha
ENSMUSG00000032999	Nlrp4f
ENSMUSG00000033033	Calhm2
ENSMUSG00000033060	Lmo7
ENSMUSG00000033107	Rnf125
ENSMUSG00000033177	Tmprss7
ENSMUSG00000033187	BC016579
ENSMUSG00000033207	Mamdc2
ENSMUSG00000033249	Hsf4
ENSMUSG00000033287	Kctd17
ENSMUSG00000033327	Tnxb
ENSMUSG00000033427	Upb1
ENSMUSG00000033491	Prss35
ENSMUSG00000033498	Strc
ENSMUSG00000033508	Asprv1
ENSMUSG00000033530	Ttc7b
ENSMUSG00000033644	Piwil2
ENSMUSG00000033685	Ucp2
ENSMUSG00000033688	1300017J02Rik
ENSMUSG00000033705	Stard9
ENSMUSG00000033722	BC034090
ENSMUSG00000033998	Kcnk1
ENSMUSG00000034035	Ccdc17
ENSMUSG00000034107	Ano7
ENSMUSG00000034112	Atp2c2
ENSMUSG00000034116	Vav1
ENSMUSG00000034156	Bzrap1

ENSMUSG00000034164	Emid1
ENSMUSG00000034205	Loxl2
ENSMUSG00000034258	Mfsd7c
ENSMUSG00000034266	Batf
ENSMUSG00000034334	Fam151b
ENSMUSG00000034416	Pkd1l2
ENSMUSG00000034438	Gbp8
ENSMUSG00000034607	Pof1b
ENSMUSG00000034640	Tiparp
ENSMUSG00000034687	Fras1
ENSMUSG00000034731	Dgkh
ENSMUSG00000034762	Glis1
ENSMUSG00000034765	Dusp5
ENSMUSG00000034768	Asb16
ENSMUSG00000034792	Gna15
ENSMUSG00000034796	Cpne7
ENSMUSG00000034825	Nrip3
ENSMUSG00000034917	Tjp3
ENSMUSG00000034918	Cdhr2
ENSMUSG00000034926	Dhcr24
ENSMUSG00000034997	Htr2a
ENSMUSG00000035121	Neil2
ENSMUSG00000035228	Ccdc106
ENSMUSG00000035246	Pcyt1b
ENSMUSG00000035298	Klhl35
ENSMUSG00000035456	Prdm8
ENSMUSG00000035459	Stab2
ENSMUSG00000035472	Slc25a21
ENSMUSG00000035498	Cdcp1
ENSMUSG00000035504	Reep6
ENSMUSG00000035557	Krt17
ENSMUSG00000035576	L3mbtl1
ENSMUSG00000035615	Frmpd1
ENSMUSG00000035681	Kcnc2
ENSMUSG00000035783	Acta2
ENSMUSG00000035852	9130017N09Rik
ENSMUSG00000035861	Tmprss11bnl
ENSMUSG00000035864	Syt1
ENSMUSG00000035868	3110052M02Rik
ENSMUSG00000035878	Agphd1
ENSMUSG00000035910	Dcdc2a
ENSMUSG00000035964	Tmem59l
ENSMUSG00000035967	Ddx26b
ENSMUSG00000036067	Slc2a6
ENSMUSG00000036083	Slc17a3
ENSMUSG00000036091	Hyal3
ENSMUSG00000036109	Mbnl3
ENSMUSG00000036136	Fam110c
ENSMUSG00000036139	Hoxc9
ENSMUSG00000036192	Rorb
ENSMUSG00000036251	Trpm8
ENSMUSG00000036295	Lrrn3
ENSMUSG00000036330	Slc18a1
ENSMUSG00000036334	Igsf10
ENSMUSG00000036437	Npy1r
ENSMUSG00000036492	Rnf39
ENSMUSG00000036564	Ndrg4
ENSMUSG00000036611	Eepd1
ENSMUSG00000036687	Tmem184a
ENSMUSG00000036805	Noxa1
ENSMUSG00000036815	Dpp10
ENSMUSG00000036856	Wnt4
ENSMUSG00000036885	Arhgef26
ENSMUSG00000036938	Try5
ENSMUSG00000037060	Prkcdbp
ENSMUSG00000037071	Scd1
ENSMUSG00000037108	Zcwpw1
ENSMUSG00000037169	Mycn
ENSMUSG00000037196	Pacrg
ENSMUSG00000037224	Zfyve28
ENSMUSG00000037375	Hhat
ENSMUSG00000037390	Muc3

ENSMUSG00000037418	Best1
ENSMUSG00000037437	Adam32
ENSMUSG00000037446	Tulp1
ENSMUSG00000037469	C330005M16Rik
ENSMUSG00000037477	Tbx10
ENSMUSG00000037493	Cib2
ENSMUSG00000037578	Pkd2l1
ENSMUSG00000037580	Gch1
ENSMUSG00000037600	1810019J16Rik
ENSMUSG00000037613	Tnfrsf23
ENSMUSG00000037636	Slc25a43
ENSMUSG00000037661	Gpr160
ENSMUSG00000037685	Atp8a1
ENSMUSG00000037705	Tecta
ENSMUSG00000037754	Ppp1r16b
ENSMUSG00000037798	Mat1a
ENSMUSG00000037813	D630003M21Rik
ENSMUSG00000037824	Tspan14
ENSMUSG00000037833	Sh2d4b
ENSMUSG00000037940	Inpp4b
ENSMUSG00000037974	Muc5ac
ENSMUSG00000038011	Dnahc10
ENSMUSG00000038067	Csf3
ENSMUSG00000038068	Rnf144b
ENSMUSG00000038135	Crygn
ENSMUSG00000038170	Pde4dip
ENSMUSG00000038209	Itln1
ENSMUSG00000038210	Hoxa11
ENSMUSG00000038217	Tlcd2
ENSMUSG00000038295	Atg9b
ENSMUSG00000038296	Galnt18
ENSMUSG00000038354	Ankrd35
ENSMUSG00000038486	Sv2a
ENSMUSG00000038495	Otud7b
ENSMUSG00000038523	1700003F12Rik
ENSMUSG00000038526	Car14
ENSMUSG00000038530	Rgs4
ENSMUSG00000038543	BC028528
ENSMUSG00000038550	Gm129
ENSMUSG00000038569	Rad9b
ENSMUSG00000038591	Colec10
ENSMUSG00000038642	Ctss
ENSMUSG00000038692	Hoxb4
ENSMUSG00000038700	Hoxb5
ENSMUSG00000038721	Hoxb7
ENSMUSG00000038725	Pkhd1l1
ENSMUSG00000038793	Lefty1
ENSMUSG00000038807	Rap1gap2
ENSMUSG00000038843	Gcnt1
ENSMUSG00000038910	Plcl2
ENSMUSG00000038963	Slco4a1
ENSMUSG00000038980	Rbbp8nl
ENSMUSG00000038997	Asb17
ENSMUSG00000039058	Ak5
ENSMUSG00000039062	Anpep
ENSMUSG00000039084	Chad
ENSMUSG00000039103	Nexn
ENSMUSG00000039131	Gipc2
ENSMUSG00000039145	Camk1d
ENSMUSG00000039193	Nlrc4
ENSMUSG00000039200	Atf7ip2
ENSMUSG00000039217	Il18
ENSMUSG00000039330	Tsga10ip
ENSMUSG00000039347	Atp6v0e2
ENSMUSG00000039357	Fut11
ENSMUSG00000039377	Hlx
ENSMUSG00000039405	Prss23
ENSMUSG00000039461	Tcta
ENSMUSG00000039476	Prrx2
ENSMUSG00000039543	Ttc18
ENSMUSG00000039563	2210406O10Rik
ENSMUSG00000039639	Kcne1

ENSMUSG00000039672	Kcne2
ENSMUSG00000039714	Cplx3
ENSMUSG00000039775	Defb3
ENSMUSG00000039787	Cercam
ENSMUSG00000039798	2600006K01Rik
ENSMUSG00000039809	Gabbr2
ENSMUSG00000039813	Tbc1d2
ENSMUSG00000039814	Xkr5
ENSMUSG00000039831	Arhgap29
ENSMUSG00000039883	Lrrc17
ENSMUSG00000039976	Tbc1d16
ENSMUSG00000040003	Magi2
ENSMUSG00000040026	Saa3
ENSMUSG00000040046	Tph1
ENSMUSG00000040138	Ndp
ENSMUSG00000040170	Fmo2
ENSMUSG00000040181	Fmo1
ENSMUSG00000040231	Syngr4
ENSMUSG00000040253	Gbp7
ENSMUSG00000040254	Sema3d
ENSMUSG00000040283	Btnl9
ENSMUSG00000040327	Cul9
ENSMUSG00000040329	Il7
ENSMUSG00000040345	Arhgap9
ENSMUSG00000040364	Sec1
ENSMUSG00000040380	Cbln3
ENSMUSG00000040387	Klhl32
ENSMUSG00000040412	5330417C22Rik
ENSMUSG00000040434	Gyltl1b
ENSMUSG00000040435	Ppp1r15a
ENSMUSG00000040441	Slc26a10
ENSMUSG00000040495	Chrm4
ENSMUSG00000040525	Cblc
ENSMUSG00000040570	Rundc3b
ENSMUSG00000040600	Eps8l3
ENSMUSG00000040612	Ildr2
ENSMUSG00000040666	Sh3bgr
ENSMUSG00000040705	A930016O22Rik
ENSMUSG00000040728	Esrp1
ENSMUSG00000040794	C1qtnf4
ENSMUSG00000040828	Catsperd
ENSMUSG00000040838	Gm11639
ENSMUSG00000040891	Foxa3
ENSMUSG00000040907	Atp1a3
ENSMUSG00000040936	Ulk4
ENSMUSG00000040969	Arhgef38
ENSMUSG00000041046	Ramp3
ENSMUSG00000041062	Mslnl
ENSMUSG00000041301	Cftr
ENSMUSG00000041329	Atp1b2
ENSMUSG00000041440	Gk5
ENSMUSG00000041556	Fbxo2
ENSMUSG00000041565	L3mbtl4
ENSMUSG00000041577	Prelp
ENSMUSG00000041608	Entpd3
ENSMUSG00000041624	Gucy1a2
ENSMUSG00000041649	Klf8
ENSMUSG00000041653	Pnpla3
ENSMUSG00000041658	Rragb
ENSMUSG00000041670	Rims1
ENSMUSG00000041695	Kcnj2
ENSMUSG00000041696	Rasl12
ENSMUSG00000041737	Tmem45b
ENSMUSG00000041827	Oasl1
ENSMUSG00000041872	Il17f
ENSMUSG00000041878	8430432A02Rik
ENSMUSG00000041986	Elmod1
ENSMUSG00000041992	Rapgef5
ENSMUSG00000042041	2010003K11Rik
ENSMUSG00000042099	Kank3
ENSMUSG00000042116	Vwa1
ENSMUSG00000042124	Lce1f

ENSMUSG00000042129	Rassf4
ENSMUSG00000042155	Klhl23
ENSMUSG00000042156	Dzip1
ENSMUSG00000042244	Pglyrp3
ENSMUSG00000042250	Pglyrp4
ENSMUSG00000042306	S100a14
ENSMUSG00000042377	Fam83g
ENSMUSG00000042439	Zfp532
ENSMUSG00000042474	Faim3
ENSMUSG00000042477	Tfap2e
ENSMUSG00000042581	Thsd7b
ENSMUSG00000042607	Asb4
ENSMUSG00000042638	Gucy2c
ENSMUSG00000042671	Rgs8
ENSMUSG00000042678	Myo15
ENSMUSG00000042734	Ttc9
ENSMUSG00000042759	Apobr
ENSMUSG00000042770	Hebp1
ENSMUSG00000042784	Muc1
ENSMUSG00000042804	Gpr153
ENSMUSG00000042807	Hecw2
ENSMUSG00000042808	Gpx2
ENSMUSG00000042826	Fgf11
ENSMUSG00000042976	9930038B18Rik
ENSMUSG00000043029	Trpv3
ENSMUSG00000043036	Ccdc63
ENSMUSG00000043085	Tmem82
ENSMUSG00000043099	Hic1
ENSMUSG00000043153	Ispd
ENSMUSG00000043155	Hpdl
ENSMUSG00000043165	Lor
ENSMUSG00000043419	A030009H04Rik
ENSMUSG00000043432	Leng9
ENSMUSG00000043461	Sptssb
ENSMUSG00000043487	Acot6
ENSMUSG00000043488	Gm9783
ENSMUSG00000043623	4933412A08Rik
ENSMUSG00000043705	Capn13
ENSMUSG00000043747	1520401A03Rik
ENSMUSG00000043782	Ccdc64b
ENSMUSG00000043789	Vwce
ENSMUSG00000044006	Cilp2
ENSMUSG00000044014	Npy5r
ENSMUSG00000044061	Olfr225
ENSMUSG00000044125	9530080O11Rik
ENSMUSG00000044165	Bcl2l15
ENSMUSG00000044250	Pced1b
ENSMUSG00000044254	Pcsk9
ENSMUSG00000044276	4933427E11Rik
ENSMUSG00000044303	Cdkn2a
ENSMUSG00000044317	Gpr4
ENSMUSG00000044337	Cxcr7
ENSMUSG00000044361	BC024139
ENSMUSG00000044362	Ccdc89
ENSMUSG00000044387	2410080I02Rik
ENSMUSG00000044471	AB041803
ENSMUSG00000044626	Liph
ENSMUSG00000044628	Rnf208
ENSMUSG00000044641	Pard6b
ENSMUSG00000044701	Il27
ENSMUSG00000044716	Dok7
ENSMUSG00000044734	Serpinb1a
ENSMUSG00000044854	1700056E22Rik
ENSMUSG00000044860	Gm1123
ENSMUSG00000044976	Wdr72
ENSMUSG00000045022	1700024P04Rik
ENSMUSG00000045102	Poln
ENSMUSG00000045281	Gpr20
ENSMUSG00000045319	Proser2
ENSMUSG00000045322	Tlr9
ENSMUSG00000045348	Nyap1
ENSMUSG00000045349	Sh2d5

ENSMUSG00000045377	Tmem88
ENSMUSG00000045391	1700120B22Rik
ENSMUSG00000045394	Epcam
ENSMUSG00000045404	Kcnk13
ENSMUSG00000045625	Pigz
ENSMUSG00000045629	Sh3tc2
ENSMUSG00000045662	Henmt1
ENSMUSG00000045746	B230317F23Rik
ENSMUSG00000045775	Slc16a5
ENSMUSG00000045871	Slitrk6
ENSMUSG00000046000	Naa11
ENSMUSG00000046057	Gm15428
ENSMUSG00000046133	C130073F10Rik
ENSMUSG00000046180	4930550L24Rik
ENSMUSG00000046186	Cd109
ENSMUSG00000046259	Sprr2h
ENSMUSG00000046352	Gjb2
ENSMUSG00000046354	Defb14
ENSMUSG00000046402	Rbp1
ENSMUSG00000046408	1700067K01Rik
ENSMUSG00000046410	Kcnk6
ENSMUSG00000046523	Kctd4
ENSMUSG00000046652	Tas2r143
ENSMUSG00000046733	Gprc5a
ENSMUSG00000046793	Gpr61
ENSMUSG00000046794	Ppp1r3b
ENSMUSG00000046804	Phgr1
ENSMUSG00000046808	Atp10d
ENSMUSG00000046840	0610008F07Rik
ENSMUSG00000046958	4930432E11Rik
ENSMUSG00000047104	Pbp2
ENSMUSG00000047146	Tet1
ENSMUSG00000047180	Neurl3
ENSMUSG00000047227	Gm527
ENSMUSG00000047228	BC048546
ENSMUSG00000047246	Hist1h2be
ENSMUSG00000047253	Krtap1-5
ENSMUSG00000047261	Gap43
ENSMUSG00000047281	Sfn
ENSMUSG00000047298	Kcnv2
ENSMUSG00000047414	Flrt2
ENSMUSG00000047473	Zfp30
ENSMUSG00000047501	Cldn4
ENSMUSG00000047515	BC049715
ENSMUSG00000047586	Nccrp1
ENSMUSG00000047641	Krt83
ENSMUSG00000047654	Tssk6
ENSMUSG00000047907	Tshz2
ENSMUSG00000047938	4930483J18Rik
ENSMUSG00000047986	Palm3
ENSMUSG00000048078	Tenm4
ENSMUSG00000048120	Entpd1
ENSMUSG00000048142	Nat8l
ENSMUSG00000048163	Selplg
ENSMUSG00000048200	Efcab4a
ENSMUSG00000048216	Gpr85
ENSMUSG00000048218	Amigo2
ENSMUSG00000048251	Bcl11b
ENSMUSG00000048373	Fgfbp1
ENSMUSG00000048387	Osr1
ENSMUSG00000048388	Fam171b
ENSMUSG00000048406	B330016D10Rik
ENSMUSG00000048450	Msx1
ENSMUSG00000048485	Zbtb8b
ENSMUSG00000048503	Tmem136
ENSMUSG00000048534	Amica1
ENSMUSG00000048583	Igf2
ENSMUSG00000048655	Ccdc169
ENSMUSG00000048763	Hoxb3
ENSMUSG00000048764	Tmprss11f
ENSMUSG00000048776	Pthlh
ENSMUSG00000048865	Arhgap30

ENSMUSG00000048992	Prss32
ENSMUSG00000049036	Tmem121
ENSMUSG00000049176	Frmpd4
ENSMUSG00000049265	Kcnk3
ENSMUSG00000049387	Cox7b2
ENSMUSG00000049476	1700104B16Rik
ENSMUSG00000049493	Pls1
ENSMUSG00000049536	Tceal1
ENSMUSG00000049538	Adamts16
ENSMUSG00000049555	Tmie
ENSMUSG00000049588	Ccdc69
ENSMUSG00000049593	Lce1h
ENSMUSG00000049604	Hoxb13
ENSMUSG00000049670	Morn4
ENSMUSG00000049687	Fam109b
ENSMUSG00000049699	Ucn2
ENSMUSG00000049721	Gal3st1
ENSMUSG00000049723	Mmp12
ENSMUSG00000049848	Ceacam19
ENSMUSG00000049892	Rasd1
ENSMUSG00000049916	2610318N02Rik
ENSMUSG00000050035	Fhl4
ENSMUSG00000050071	Bex1
ENSMUSG00000050105	Grrp1
ENSMUSG00000050150	Slc9b1
ENSMUSG00000050192	Eif5a2
ENSMUSG00000050212	Eva1b
ENSMUSG00000050232	Cxcr3
ENSMUSG00000050296	Abca12
ENSMUSG00000050424	Pnma5
ENSMUSG00000050425	Mrgprb2
ENSMUSG00000050463	Krt78
ENSMUSG00000050538	B230217C12Rik
ENSMUSG00000050556	Kcnb1
ENSMUSG00000050612	Txndc2
ENSMUSG00000050747	Trim15
ENSMUSG00000050777	Tmem37
ENSMUSG00000050821	Fam131a
ENSMUSG00000050854	Tmem125
ENSMUSG00000051048	P4ha3
ENSMUSG00000051065	Mb21d2
ENSMUSG00000051074	4930579K19Rik
ENSMUSG00000051113	Fam71e1
ENSMUSG00000051228	Nyx
ENSMUSG00000051335	Gfod1
ENSMUSG00000051373	Ppapdc3
ENSMUSG00000051379	Flrt3
ENSMUSG00000051397	Tacstd2
ENSMUSG00000051431	Gpr87
ENSMUSG00000051506	Wdfy4
ENSMUSG00000051648	Kctd19
ENSMUSG00000051769	Wfdc15a
ENSMUSG00000051910	Sox6
ENSMUSG00000052117	D630039A03Rik
ENSMUSG00000052131	Akr1b7
ENSMUSG00000052160	Pld4
ENSMUSG00000052180	Serpinb6c
ENSMUSG00000052212	Cd177
ENSMUSG00000052276	Ostn
ENSMUSG00000052281	Gm15024
ENSMUSG00000052334	1700024B05Rik
ENSMUSG00000052384	Lrrc33
ENSMUSG00000052468	Pmp2
ENSMUSG00000052572	Dlg2
ENSMUSG00000052616	Ccdc79
ENSMUSG00000052631	Sh2d6
ENSMUSG00000052632	Asap2
ENSMUSG00000052658	5830454E08Rik
ENSMUSG00000052760	A630001G21Rik
ENSMUSG00000052819	Best2
ENSMUSG00000052821	Cysltr1
ENSMUSG00000052825	Gm9892

ENSMUSG00000052921	Arhgef15
ENSMUSG00000053024	Cntn2
ENSMUSG00000053062	Jam2
ENSMUSG00000053121	Gm5129
ENSMUSG00000053141	Ptprt
ENSMUSG00000053158	Fes
ENSMUSG00000053182	Gm609
ENSMUSG00000053358	Gm9905
ENSMUSG00000053522	Lgals7
ENSMUSG00000053675	Tgm5
ENSMUSG00000053687	Dpep2
ENSMUSG00000053702	Nebl
ENSMUSG00000053819	Camk2d
ENSMUSG00000053825	Ppfia2
ENSMUSG00000053846	Lipg
ENSMUSG00000053889	A130066N16Rik
ENSMUSG00000053909	Rhox10
ENSMUSG00000053964	Lgals4
ENSMUSG00000054006	D630008O14Rik
ENSMUSG00000054065	Pkp3
ENSMUSG00000054146	Krt15
ENSMUSG00000054150	Syne3
ENSMUSG00000054181	A930012O16Rik
ENSMUSG00000054215	Sprr2k
ENSMUSG00000054385	Ceacam2
ENSMUSG00000054404	Slfn5
ENSMUSG00000054426	A930005H10Rik
ENSMUSG00000054537	Tmprss11e
ENSMUSG00000054580	Pla2r1
ENSMUSG00000054598	9130230L23Rik
ENSMUSG00000054622	D730045B01Rik
ENSMUSG00000054626	Xlr
ENSMUSG00000054675	Tmem119
ENSMUSG00000054753	AU018091
ENSMUSG00000054850	Ncrna00086
ENSMUSG00000054889	Dsp
ENSMUSG00000054892	Txk
ENSMUSG00000054901	Arhgef33
ENSMUSG00000054932	Afp
ENSMUSG00000054945	Gm9958
ENSMUSG00000055148	Klf2
ENSMUSG00000055159	4930583K01Rik
ENSMUSG00000055271	9330161L09Rik
ENSMUSG00000055313	Pgbd1
ENSMUSG00000055333	Fat2
ENSMUSG00000055415	Atp10b
ENSMUSG00000055446	A630091E08Rik
ENSMUSG00000055497	Gm9974
ENSMUSG00000055523	Gucy2g
ENSMUSG00000055560	Zfp459
ENSMUSG00000055691	Gja6
ENSMUSG00000055733	Nap1l3
ENSMUSG00000055865	Fam19a3
ENSMUSG00000055882	Abhd16b
ENSMUSG00000055960	Skint4
ENSMUSG00000055976	Cldn23
ENSMUSG00000055978	Fut2
ENSMUSG00000056054	S100a8
ENSMUSG00000056071	S100a9
ENSMUSG00000056089	Gm5468
ENSMUSG00000056130	Ticam2
ENSMUSG00000056203	Tas2r135
ENSMUSG00000056271	Lman1l
ENSMUSG00000056457	Prl2c3
ENSMUSG00000056481	Cd248
ENSMUSG00000056498	Tmem154
ENSMUSG00000056508	1700001K19Rik
ENSMUSG00000056632	Dsg3
ENSMUSG00000056671	Prelid2
ENSMUSG00000056673	Kdm5d
ENSMUSG00000056753	C330011M18Rik
ENSMUSG00000056771	Gm10010

ENSMUSG00000056895	Hist3h2ba
ENSMUSG00000056899	Immp2l
ENSMUSG00000056947	Mab21l1
ENSMUSG00000057000	Nxf3
ENSMUSG00000057060	Slc35f3
ENSMUSG00000057092	Fxyd3
ENSMUSG00000057116	AF366264
ENSMUSG00000057123	Gja5
ENSMUSG00000057163	Prss2
ENSMUSG00000057286	St6galnac2
ENSMUSG00000057337	Chst3
ENSMUSG00000057454	Lypd3
ENSMUSG00000057457	Phex
ENSMUSG00000057836	Xlr3a
ENSMUSG00000057880	Abat
ENSMUSG00000058063	Trim31
ENSMUSG00000058126	Tpm3-rs7
ENSMUSG00000058147	Xlr3c
ENSMUSG00000058186	Gm13242
ENSMUSG00000058354	Krt6a
ENSMUSG00000058498	Rnf207
ENSMUSG00000058589	Anks1b
ENSMUSG00000058626	Capn11
ENSMUSG00000058656	Samd12
ENSMUSG00000058794	Nfe2
ENSMUSG00000058831	Opn1sw
ENSMUSG00000059108	Ifitm6
ENSMUSG00000059213	Ddn
ENSMUSG00000059237	Ppp1r2-ps4
ENSMUSG00000059327	Eda
ENSMUSG00000059334	Zfp36l3
ENSMUSG00000059336	Slc14a1
ENSMUSG00000059395	Nkapl
ENSMUSG00000059430	Actg2
ENSMUSG00000059461	Gm7331
ENSMUSG00000059565	Gm5292
ENSMUSG00000059588	Calcrl
ENSMUSG00000059639	Clec4a4
ENSMUSG00000059659	Gm10069
ENSMUSG00000059668	Krt4
ENSMUSG00000059832	Kprp
ENSMUSG00000059900	Tmem40
ENSMUSG00000059974	Ntm
ENSMUSG00000059994	Fcrl1
ENSMUSG00000060188	Cxcl17
ENSMUSG00000060224	Pyroxd2
ENSMUSG00000060256	Tdpoz4
ENSMUSG00000060314	Zfp941
ENSMUSG00000060509	Xcr1
ENSMUSG00000060579	Fhit
ENSMUSG00000060639	Hist1h4i
ENSMUSG00000060716	Plekhh1
ENSMUSG00000060794	Tssk5
ENSMUSG00000060878	Olfr1420
ENSMUSG00000060923	Acyp2
ENSMUSG00000060962	Dmkn
ENSMUSG00000061048	Cdh3
ENSMUSG00000061119	Prcp
ENSMUSG00000061356	Nuggc
ENSMUSG00000061414	Efcab4b
ENSMUSG00000061510	Gm10101
ENSMUSG00000061517	Sox21
ENSMUSG00000061544	Zfp229
ENSMUSG00000061577	Gpr114
ENSMUSG00000061654	
ENSMUSG00000061728	Btnl7
ENSMUSG00000061769	Klra6
ENSMUSG00000061897	Gm14292
ENSMUSG00000062012	Zfp13
ENSMUSG00000062278	Gm11562
ENSMUSG00000062345	Serpinb2
ENSMUSG00000062432	Cyp26c1

ENSMUSG00000062515	Fabp4
ENSMUSG00000062518	Zfp534
ENSMUSG00000062611	Rps3a2
ENSMUSG00000062713	Sim2
ENSMUSG00000062937	Mtap
ENSMUSG00000062980	Cped1
ENSMUSG00000063011	Msln
ENSMUSG00000063314	Gm12657
ENSMUSG00000063376	Ifna13
ENSMUSG00000063458	1700112E06Rik
ENSMUSG00000063522	2010109I03Rik
ENSMUSG00000063531	Sema3e
ENSMUSG00000063651	Cnfn
ENSMUSG00000063689	Hist2h2ab
ENSMUSG00000063767	S100a7a
ENSMUSG00000063804	Lin28b
ENSMUSG00000063971	1700011A15Rik
ENSMUSG00000064125	BC068157
ENSMUSG00000064262	Gimap8
ENSMUSG00000064267	Hvcn1
ENSMUSG00000064315	Rpl21-ps10
ENSMUSG00000064380	Gm26448
ENSMUSG00000064390	Rnu73b
ENSMUSG00000064427	Gm22748
ENSMUSG00000064493	Snora28
ENSMUSG00000064500	Gm25296
ENSMUSG00000064513	Gm22457
ENSMUSG00000064600	Gm25636
ENSMUSG00000064637	Snora20
ENSMUSG00000064655	Gm25788
ENSMUSG00000064672	Gm22806
ENSMUSG00000064696	Gm24148
ENSMUSG00000064702	Gm24950
ENSMUSG00000064791	Snord14e
ENSMUSG00000064856	Gm23444
ENSMUSG00000064899	Snord118
ENSMUSG00000064925	Snora62
ENSMUSG00000064952	Gm24920
ENSMUSG00000065016	Snora3
ENSMUSG00000065036	Gm22362
ENSMUSG00000065061	Gm22884
ENSMUSG00000065089	Gm26493
ENSMUSG00000065094	Snord1a
ENSMUSG00000065118	Gm23297
ENSMUSG00000065176	Rnu12
ENSMUSG00000065196	Snord85
ENSMUSG00000065232	Gm22973
ENSMUSG00000065251	Gm23971
ENSMUSG00000065262	Gm23451
ENSMUSG00000065273	Gm25128
ENSMUSG00000065362	Gm24411
ENSMUSG00000065555	Mir219-1
ENSMUSG00000065628	Snord33
ENSMUSG00000065649	Snora74a
ENSMUSG00000065686	Snora5c
ENSMUSG00000065701	Rny1
ENSMUSG00000065725	Gm26165
ENSMUSG00000065738	Gm24494
ENSMUSG00000065750	Gm23346
ENSMUSG00000065767	Gm23849
ENSMUSG00000065773	Rnu1b6
ENSMUSG00000065778	Gm22154
ENSMUSG00000065820	Gm26316
ENSMUSG00000065847	Gm25188
ENSMUSG00000065870	Rnu3a
ENSMUSG00000065899	Gm24523
ENSMUSG00000065904	Gm26109
ENSMUSG00000065944	Rnu2-10
ENSMUSG00000065952	C330021F23Rik
ENSMUSG00000066000	2610305D13Rik
ENSMUSG00000066027	Gm10436
ENSMUSG00000066175	2510046G10Rik

ENSMUSG00000066176	Gm12511
ENSMUSG00000066178	6030445D17Rik
ENSMUSG00000066720	Cldn9
ENSMUSG00000066721	Zfp575
ENSMUSG00000066861	Oas1g
ENSMUSG00000067006	Serpinb5
ENSMUSG00000067206	Lrrc66
ENSMUSG00000067219	Nipal1
ENSMUSG00000067231	Cyp2c65
ENSMUSG00000067276	Capn6
ENSMUSG00000067370	B3galt4
ENSMUSG00000067389	Gm17080
ENSMUSG00000067455	Hist1h4j
ENSMUSG00000067714	Lpar5
ENSMUSG00000067736	Gm10222
ENSMUSG00000067768	Xlr4b
ENSMUSG00000067771	Gm14685
ENSMUSG00000067780	Pi15
ENSMUSG00000067795	4930444P10Rik
ENSMUSG00000067813	Xkr9
ENSMUSG00000067818	Myl9
ENSMUSG00000067855	Speer3
ENSMUSG00000067889	Sptbn2
ENSMUSG00000067928	Zfp760
ENSMUSG00000068011	2510049J12Rik
ENSMUSG00000068129	Cst7
ENSMUSG00000068154	Insm1
ENSMUSG00000068196	Col8a1
ENSMUSG00000068303	Spr-ps1
ENSMUSG00000068327	Tlx2
ENSMUSG00000068399	Gm7247
ENSMUSG00000068457	Uty
ENSMUSG00000068617	Efcab1
ENSMUSG00000068699	Flnc
ENSMUSG00000068745	Mybphl
ENSMUSG00000068762	Gstm6
ENSMUSG00000068854	Hist2h2be
ENSMUSG00000068855	Hist2h2ac
ENSMUSG00000068888	Lce1i
ENSMUSG00000068893	Sprr2a2
ENSMUSG00000069045	Ddx3y
ENSMUSG00000069049	Eif2s3y
ENSMUSG00000069170	Gpr98
ENSMUSG00000069171	Nr2f1
ENSMUSG00000069270	Hist1h2ac
ENSMUSG00000069302	Hist1h2ah
ENSMUSG00000069307	Hist1h2bq
ENSMUSG00000069308	Hist1h2bp
ENSMUSG00000069554	I830134H01Rik
ENSMUSG00000069622	Gm10273
ENSMUSG00000069713	4933406P04Rik
ENSMUSG00000069814	E130309D14Rik
ENSMUSG00000069825	Spata22
ENSMUSG00000069833	Ahnak
ENSMUSG00000069835	Sat2
ENSMUSG00000069873	4930438A08Rik
ENSMUSG00000069917	Hba-a2
ENSMUSG00000069920	B3gnt9
ENSMUSG00000070111	Gm10286
ENSMUSG00000070287	Slc35g2
ENSMUSG00000070305	Mpzl3
ENSMUSG00000070313	A630095E13Rik
ENSMUSG00000070315	4930581F22Rik
ENSMUSG00000070385	Ampd1
ENSMUSG00000070392	Gm20634
ENSMUSG00000070424	Art5
ENSMUSG00000070436	Serpinh1
ENSMUSG00000070498	Tmem132b
ENSMUSG00000070546	Mrgprb3
ENSMUSG00000070661	Rnf186
ENSMUSG00000070687	Htr1d
ENSMUSG00000070720	Tmem200b

ENSMUSG00000070960	Gm10307
ENSMUSG00000070997	1700055D18Rik
ENSMUSG00000071042	Rasgrp3
ENSMUSG00000071113	Mboat4
ENSMUSG00000071141	Rpl36a-ps3
ENSMUSG00000071553	Cpa2
ENSMUSG00000071573	Rnls
ENSMUSG00000071671	Gm10343
ENSMUSG00000071691	Gm960
ENSMUSG00000071893	Vmn1r4
ENSMUSG00000072573	Gm10369
ENSMUSG00000072618	Gm10384
ENSMUSG00000072621	Slfn10-ps
ENSMUSG00000072660	Gm6288
ENSMUSG00000072673	Gm10392
ENSMUSG00000072774	Zfp951
ENSMUSG00000072884	Gm10433
ENSMUSG00000072905	Gm2016
ENSMUSG00000072934	Gm15070
ENSMUSG00000072962	Gm16401
ENSMUSG00000073176	Zfp449
ENSMUSG00000073177	Gm773
ENSMUSG00000073234	Gm8773
ENSMUSG00000073274	Gm14636
ENSMUSG00000073399	Trim40
ENSMUSG00000073400	Trim10
ENSMUSG00000073414	AU023871
ENSMUSG00000073482	Gm10517
ENSMUSG00000073529	F830208F22Rik
ENSMUSG00000073538	E330020D12Rik
ENSMUSG00000073565	Prr16
ENSMUSG00000073568	Arl14epl
ENSMUSG00000073590	3222401L13Rik
ENSMUSG00000073598	1700066B19Rik
ENSMUSG00000073680	Tmem88b
ENSMUSG00000073739	Gm16287
ENSMUSG00000073755	5730409E04Rik
ENSMUSG00000073758	Sh3d21
ENSMUSG00000073795	6430531B16Rik
ENSMUSG00000073802	Cdkn2b
ENSMUSG00000074024	4632427E13Rik
ENSMUSG00000074056	Gm10615
ENSMUSG00000074065	Gm10617
ENSMUSG00000074146	4930579C12Rik
ENSMUSG00000074149	Gm10634
ENSMUSG00000074199	Krtdap
ENSMUSG00000074215	Gm10643
ENSMUSG00000074217	2210011C24Rik
ENSMUSG00000074219	Gm10644
ENSMUSG00000074228	Gm10645
ENSMUSG00000074232	Gm10647
ENSMUSG00000074240	Cib3
ENSMUSG00000074256	Gm10655
ENSMUSG00000074259	Gramd2
ENSMUSG00000074272	Ceacam1
ENSMUSG00000074280	Gm6166
ENSMUSG00000074283	Zfp109
ENSMUSG00000074300	BC030870
ENSMUSG00000074398	Gm15441
ENSMUSG00000074445	Sprr2a3
ENSMUSG00000074489	Bglap3
ENSMUSG00000074527	Gm14296
ENSMUSG00000074579	Lekr1
ENSMUSG00000074625	Arhgap40
ENSMUSG00000074653	Lrrc31
ENSMUSG00000074731	Zfp345
ENSMUSG00000074766	Ism1
ENSMUSG00000074771	Ankef1
ENSMUSG00000074794	Arrdc3
ENSMUSG00000074796	Slc4a11
ENSMUSG00000074881	Mageb3
ENSMUSG00000074916	Chst14

ENSMUSG00000074973	Gm11382
ENSMUSG00000075044	Slc22a29
ENSMUSG00000075224	Lrrc55
ENSMUSG00000075225	Ccdc162
ENSMUSG00000075271	Ttc30a1
ENSMUSG00000075272	Ttc30a2
ENSMUSG00000075325	Gm13582
ENSMUSG00000075394	Hoxc4
ENSMUSG00000075416	Gm14488
ENSMUSG00000075420	Smim6
ENSMUSG00000075465	Gm10837
ENSMUSG00000075480	Gm10840
ENSMUSG00000075502	Zbtbd6
ENSMUSG00000075511	1700001L05Rik
ENSMUSG00000075553	Gm5464
ENSMUSG00000075589	Gm11536
ENSMUSG00000075592	Nynrin
ENSMUSG00000075593	Gal3st4
ENSMUSG00000076434	Wfdc3
ENSMUSG00000076435	Acsf2
ENSMUSG00000076439	Mog
ENSMUSG00000076490	Trbc1
ENSMUSG00000076499	Trbv31
ENSMUSG00000076757	Tcrg-C4
ENSMUSG00000076867	Trdv4
ENSMUSG00000076897	Traj32
ENSMUSG00000076928	Trac
ENSMUSG00000077192	Snora17
ENSMUSG00000077274	Gm22786
ENSMUSG00000077349	Gm24867
ENSMUSG00000077426	Gm26387
ENSMUSG00000077440	Gm23130
ENSMUSG00000077493	Snord91a
ENSMUSG00000077611	Gm23946
ENSMUSG00000077709	Snora64
ENSMUSG00000077711	AF357399
ENSMUSG00000077714	Snord17
ENSMUSG00000077797	Snord19
ENSMUSG00000078087	Rps12l1
ENSMUSG00000078377	Gm4294
ENSMUSG00000078439	2210404O07Rik
ENSMUSG00000078486	2310042D19Rik
ENSMUSG00000078497	Gm13145
ENSMUSG00000078498	Gm13151
ENSMUSG00000078519	2310026L22Rik
ENSMUSG00000078588	Ccdc24
ENSMUSG00000078640	Gm11627
ENSMUSG00000078664	Sprr2a1
ENSMUSG00000078668	Gm11595
ENSMUSG00000078865	Gm14406
ENSMUSG00000078867	Gm14418
ENSMUSG00000078868	Gm14412
ENSMUSG00000078869	Gm14409
ENSMUSG00000078872	Gm14401
ENSMUSG00000078875	Gm14419
ENSMUSG00000078889	Gm14288
ENSMUSG00000078902	Gm14443
ENSMUSG00000078905	Gm14393
ENSMUSG00000078915	Hsp25-ps1
ENSMUSG00000078931	Pdf
ENSMUSG00000078952	Gm2373
ENSMUSG00000078954	Arhgap8
ENSMUSG00000079008	Gm14124
ENSMUSG00000079009	Gm14139
ENSMUSG00000079029	Gm5662
ENSMUSG00000079049	Serpinb1c
ENSMUSG00000079057	Cyp4v3
ENSMUSG00000079069	8430423G03Rik
ENSMUSG00000079092	Prl2c2
ENSMUSG00000079173	Zan
ENSMUSG00000079223	Gm8778
ENSMUSG00000079243	Xirp1

ENSMUSG00000079244	Gm5622
ENSMUSG00000079265	Gm8257
ENSMUSG00000079267	Gm5930
ENSMUSG00000079271	1700049E17Rik1
ENSMUSG00000079304	4933413G19Rik
ENSMUSG00000079330	Lemd1
ENSMUSG00000079356	Mettl4-ps1
ENSMUSG00000079407	1700110I01Rik
ENSMUSG00000079428	Tceal7
ENSMUSG00000079440	Alpi
ENSMUSG00000079442	St6galnac4
ENSMUSG00000079467	Gm14966
ENSMUSG00000079502	1700101E01Rik
ENSMUSG00000079620	Muc4
ENSMUSG00000079627	Rhox2h
ENSMUSG00000079662	Ntn3
ENSMUSG00000079834	
ENSMUSG00000079852	Klra4
ENSMUSG00000080076	Hist1h2aj
ENSMUSG00000080223	Gm13768
ENSMUSG00000080352	Gm26247
ENSMUSG00000080365	Gm25776
ENSMUSG00000080518	Gm22193
ENSMUSG00000080540	Gm22711
ENSMUSG00000080542	Gm22710
ENSMUSG00000080712	Hist3h2bb-ps
ENSMUSG00000080728	Gm13616
ENSMUSG00000080941	Gm16056
ENSMUSG00000080956	Gm16179
ENSMUSG00000080999	Gm11336
ENSMUSG00000081058	Hist2h3c2
ENSMUSG00000081169	Gm12551
ENSMUSG00000081187	3110067C02Rik
ENSMUSG00000081282	Gm13836
ENSMUSG00000081355	Gm15264
ENSMUSG00000081399	Gm14921
ENSMUSG00000081490	Gm11830
ENSMUSG00000081506	Amy2-ps1
ENSMUSG00000081550	Gm15877
ENSMUSG00000081590	Gm14413
ENSMUSG00000081604	Gm11518
ENSMUSG00000081671	Gm13167
ENSMUSG00000081672	Gm13000
ENSMUSG00000081755	Gm12574
ENSMUSG00000081756	Gm12291
ENSMUSG00000081784	Gm13281
ENSMUSG00000081901	Gm15357
ENSMUSG00000082023	Gm14407
ENSMUSG00000082051	Gm16072
ENSMUSG00000082082	Gm13230
ENSMUSG00000082088	Gm15753
ENSMUSG00000082149	Gm13002
ENSMUSG00000082188	Gm2768
ENSMUSG00000082244	Gm8503
ENSMUSG00000082271	Gm12217
ENSMUSG00000082321	Gm14253
ENSMUSG00000082329	Gm14287
ENSMUSG00000082361	Btc
ENSMUSG00000082377	Gm14415
ENSMUSG00000082380	Gm12218
ENSMUSG00000082419	Gm11425
ENSMUSG00000082436	Gm11688
ENSMUSG00000082585	Gm15387
ENSMUSG00000082718	Gm14928
ENSMUSG00000082724	Gm14416
ENSMUSG00000082729	Gm14845
ENSMUSG00000082848	Gm16066
ENSMUSG00000082867	Gm15730
ENSMUSG00000083013	Gm12978
ENSMUSG00000083161	Gm11427
ENSMUSG00000083170	Gm14405
ENSMUSG00000083202	Gm12388

ENSMUSG00000083317	Gm14402
ENSMUSG00000083332	Gm7599
ENSMUSG00000083365	Gm12895
ENSMUSG00000083650	Gm13357
ENSMUSG00000083732	Gm14197
ENSMUSG00000083915	Gm13932
ENSMUSG00000084050	Gm14439
ENSMUSG00000084064	Gm12079
ENSMUSG00000084083	Gm15782
ENSMUSG00000084128	Esrp2
ENSMUSG00000084157	Gm15576
ENSMUSG00000084169	Gm12240
ENSMUSG00000084390	Gm15425
ENSMUSG00000084616	Gm25047
ENSMUSG00000084666	Gm23211
ENSMUSG00000084686	Gm22027
ENSMUSG00000084706	Gm22987
ENSMUSG00000084708	Gm22988
ENSMUSG00000084793	Gm2568
ENSMUSG00000084797	Gm14321
ENSMUSG00000084803	5830444B04Rik
ENSMUSG00000084806	Gm15232
ENSMUSG00000084809	Gm12631
ENSMUSG00000084844	Hoxb3os
ENSMUSG00000084855	Gm9850
ENSMUSG00000084858	Gm1980
ENSMUSG00000084894	Gm13834
ENSMUSG00000084920	Gm15230
ENSMUSG00000084938	BB557941
ENSMUSG00000084945	C030037F17Rik
ENSMUSG00000084947	Gm15594
ENSMUSG00000085006	BC021767
ENSMUSG00000085016	Gm11335
ENSMUSG00000085022	Gm5860
ENSMUSG00000085023	Gm12744
ENSMUSG00000085025	Gm13715
ENSMUSG00000085049	A230071N21Rik
ENSMUSG00000085054	Gm15834
ENSMUSG00000085068	Gm15895
ENSMUSG00000085085	1700086P04Rik
ENSMUSG00000085101	Gm13227
ENSMUSG00000085103	Gm16357
ENSMUSG00000085135	Gm13713
ENSMUSG00000085143	Gm11520
ENSMUSG00000085152	Gm11496
ENSMUSG00000085171	D830026I12Rik
ENSMUSG00000085183	Gm12603
ENSMUSG00000085185	BC028777
ENSMUSG00000085186	Gm14377
ENSMUSG00000085196	Gm14963
ENSMUSG00000085201	Gm13507
ENSMUSG00000085204	Gm15327
ENSMUSG00000085250	Gm15234
ENSMUSG00000085260	1700013G23Rik
ENSMUSG00000085261	Gm13814
ENSMUSG00000085264	Gm15581
ENSMUSG00000085274	BC046401
ENSMUSG00000085282	Gm15663
ENSMUSG00000085285	Gm14968
ENSMUSG00000085303	Gm16751
ENSMUSG00000085329	2810404F17Rik
ENSMUSG00000085354	Gm2044
ENSMUSG00000085363	Gm15478
ENSMUSG00000085395	Gm13056
ENSMUSG00000085397	Gm13524
ENSMUSG00000085412	Gm15055
ENSMUSG00000085449	Gm15520
ENSMUSG00000085524	Gm14224
ENSMUSG00000085526	Gm16083
ENSMUSG00000085542	1700037C06Rik
ENSMUSG00000085547	Gm13767
ENSMUSG00000085569	Gm12602

ENSMUSG00000085573	Gm15418
ENSMUSG00000085584	Rgag1
ENSMUSG00000085589	A430078I02Rik
ENSMUSG00000085611	Ap3s1-ps1
ENSMUSG00000085623	Gm16041
ENSMUSG00000085645	0610040B09Rik
ENSMUSG00000085704	4921531C22Rik
ENSMUSG00000085705	Gm16046
ENSMUSG00000085725	Gm15873
ENSMUSG00000085735	4930550G17Rik
ENSMUSG00000085760	Gm14302
ENSMUSG00000085763	2700081L22Rik
ENSMUSG00000085772	D630024D03Rik
ENSMUSG00000085788	4930470G03Rik
ENSMUSG00000085829	Gm4285
ENSMUSG00000085830	E130003G02Rik
ENSMUSG00000085833	Gm13003
ENSMUSG00000085843	A730085A09Rik
ENSMUSG00000085912	Trp53cor1
ENSMUSG00000085920	Gm12951
ENSMUSG00000085929	Gm13421
ENSMUSG00000085975	Gm13572
ENSMUSG00000085984	1700001G11Rik
ENSMUSG00000085995	Gm2788
ENSMUSG00000086013	Gm15706
ENSMUSG00000086021	Gm15767
ENSMUSG00000086032	Gm15929
ENSMUSG00000086043	Gm12473
ENSMUSG00000086108	Gm5602
ENSMUSG00000086141	9030622O22Rik
ENSMUSG00000086148	Gm15271
ENSMUSG00000086163	Gm14206
ENSMUSG00000086172	2700068H02Rik
ENSMUSG00000086179	Gm14317
ENSMUSG00000086184	Gm12764
ENSMUSG00000086202	1700019E08Rik
ENSMUSG00000086256	Gm12052
ENSMUSG00000086264	Gm15850
ENSMUSG00000086266	Igf2as
ENSMUSG00000086292	Gm16052
ENSMUSG00000086296	D030055H07Rik
ENSMUSG00000086320	Gm12840
ENSMUSG00000086340	1810059C17Rik
ENSMUSG00000086350	B230369F24Rik
ENSMUSG00000086368	Gm13830
ENSMUSG00000086390	1810019D21Rik
ENSMUSG00000086395	A630014C17Rik
ENSMUSG00000086424	Gm15569
ENSMUSG00000086427	Hoxa11as
ENSMUSG00000086443	4933421A08Rik
ENSMUSG00000086467	4930571N24Rik
ENSMUSG00000086478	Gm14102
ENSMUSG00000086527	Gm15856
ENSMUSG00000086549	Gm13648
ENSMUSG00000086552	A730090H04Rik
ENSMUSG00000086554	9530034E10Rik
ENSMUSG00000086578	Gm13583
ENSMUSG00000086605	Gm14290
ENSMUSG00000086638	4930405A21Rik
ENSMUSG00000086646	5133400J02Rik
ENSMUSG00000086657	4921530L18Rik
ENSMUSG00000086677	Gm12284
ENSMUSG00000086681	Gm16178
ENSMUSG00000086741	Gm15816
ENSMUSG00000086742	Gm16201
ENSMUSG00000086762	Gm14546
ENSMUSG00000086775	5730588L14Rik
ENSMUSG00000086820	Gm11465
ENSMUSG00000086825	Gm15675
ENSMUSG00000086827	4930461C15Rik
ENSMUSG00000086828	Gm15579
ENSMUSG00000086847	Gm16065

ENSMUSG00000086852	Gm11374
ENSMUSG00000086860	Gm1720
ENSMUSG00000086867	Gm4577
ENSMUSG00000086899	1600002D24Rik
ENSMUSG00000086905	Gm13716
ENSMUSG00000086914	Gm16124
ENSMUSG00000086918	4930429F24Rik
ENSMUSG00000086938	4930481A15Rik
ENSMUSG00000086943	4732414G09Rik
ENSMUSG00000086961	Gm12946
ENSMUSG00000086969	4930443O20Rik
ENSMUSG00000086993	Gm15623
ENSMUSG00000087107	AI662270
ENSMUSG00000087121	Gm7847
ENSMUSG00000087125	A230108P19Rik
ENSMUSG00000087135	Gm16096
ENSMUSG00000087143	A830082K12Rik
ENSMUSG00000087166	L1td1
ENSMUSG00000087176	D230022J07Rik
ENSMUSG00000087201	Gm15261
ENSMUSG00000087207	Gm13147
ENSMUSG00000087211	1500016L03Rik
ENSMUSG00000087268	Gm14486
ENSMUSG00000087293	Gm14341
ENSMUSG00000087305	A430035B10Rik
ENSMUSG00000087307	Gm12925
ENSMUSG00000087312	Gm13833
ENSMUSG00000087326	Gm12503
ENSMUSG00000087330	Gm11616
ENSMUSG00000087342	Gm12238
ENSMUSG00000087351	Gm11464
ENSMUSG00000087362	Gm13710
ENSMUSG00000087371	Gm15541
ENSMUSG00000087377	AV099323
ENSMUSG00000087389	Gm15592
ENSMUSG00000087397	Gm16256
ENSMUSG00000087408	Cers1
ENSMUSG00000087413	Gm11266
ENSMUSG00000087417	Gm13001
ENSMUSG00000087433	Gm14167
ENSMUSG00000087434	Gm11202
ENSMUSG00000087475	4933406I18Rik
ENSMUSG00000087496	Gm13523
ENSMUSG00000087535	Zmiz1as1
ENSMUSG00000087575	Gm12976
ENSMUSG00000087624	9230111E07Rik
ENSMUSG00000087669	Gm11724
ENSMUSG00000087678	Gm14120
ENSMUSG00000087685	1700122E12Rik
ENSMUSG00000087691	Gm15674
ENSMUSG00000087790	Gm25970
ENSMUSG00000087963	Gm25394
ENSMUSG00000088000	Gm25493
ENSMUSG00000088030	Gm24991
ENSMUSG00000088108	Snora47
ENSMUSG00000088252	Snord13
ENSMUSG00000088254	Gm24289
ENSMUSG00000088308	Gm24507
ENSMUSG00000088326	Gm25667
ENSMUSG00000088420	Gm22551
ENSMUSG00000088459	Mir1941
ENSMUSG00000088524	Snord2
ENSMUSG00000088562	Gm23780
ENSMUSG00000088604	Gm25820
ENSMUSG00000088856	Gm24727
ENSMUSG00000088990	Gm22767
ENSMUSG00000089093	Snord11
ENSMUSG00000089371	Mir1938
ENSMUSG00000089512	Gm26287
ENSMUSG00000089697	Gm15947
ENSMUSG00000089712	Gm15889
ENSMUSG00000089812	Gm15867

ENSMUSG00000089827	1700023H06Rik
ENSMUSG00000089851	Gm16579
ENSMUSG00000089876	Tmem102
ENSMUSG00000089889	0610040B10Rik
ENSMUSG00000089983	2010320O07Rik
ENSMUSG00000089996	Tmsb15b2
ENSMUSG00000090030	A430072P03Rik
ENSMUSG00000090041	Gm16027
ENSMUSG00000090105	Gm15890
ENSMUSG00000090118	Gm16163
ENSMUSG00000090176	Cd200r2
ENSMUSG00000090207	4930524O07Rik
ENSMUSG00000090263	D730045A05Rik
ENSMUSG00000090317	Gm17324
ENSMUSG00000090358	Gm2822
ENSMUSG00000090379	Gm8229
ENSMUSG00000090386	2810055G20Rik
ENSMUSG00000090440	Gm9732
ENSMUSG00000090509	Sfta2
ENSMUSG00000090533	Gm8214
ENSMUSG00000090534	Gm4675
ENSMUSG00000090576	Gm17055
ENSMUSG00000090588	Gm9573
ENSMUSG00000090631	Olfr456
ENSMUSG00000090634	Gm8126
ENSMUSG00000090698	Apold1
ENSMUSG00000090713	Gm8127
ENSMUSG00000090788	Gm9597
ENSMUSG00000090793	Gm6650
ENSMUSG00000091007	D630036H23Rik
ENSMUSG00000091110	Gm2832
ENSMUSG00000091248	Gm17689
ENSMUSG00000091296	Gm4181
ENSMUSG00000091329	1700112D23Rik
ENSMUSG00000091378	Gm4219
ENSMUSG00000091393	5330438I03Rik
ENSMUSG00000091405	Hist2h4
ENSMUSG00000091412	Gm2895
ENSMUSG00000091477	Gm5799
ENSMUSG00000091575	2010016I18Rik
ENSMUSG00000091635	Vmn2r13
ENSMUSG00000091698	Gm6526
ENSMUSG00000091725	Gm8220
ENSMUSG00000091900	Gm4353
ENSMUSG00000091923	Gm8267
ENSMUSG00000091947	6530401F13Rik
ENSMUSG00000091993	B930036N10Rik
ENSMUSG00000091997	Gm6611
ENSMUSG00000092009	Myh15
ENSMUSG00000092071	A230065N10Rik
ENSMUSG00000092165	Gm5624
ENSMUSG00000092200	Tnxa
ENSMUSG00000092239	1700031A10Rik
ENSMUSG00000092275	Gm20465
ENSMUSG00000092305	Prps1l1
ENSMUSG00000092389	Gm20483
ENSMUSG00000092400	Gm20469
ENSMUSG00000092460	Gm8878
ENSMUSG00000092482	Gm20531
ENSMUSG00000092511	Gm20547
ENSMUSG00000092518	Fam71e2
ENSMUSG00000092521	Gm4271
ENSMUSG00000092572	Serpinb10
ENSMUSG00000092574	2810047C21Rik1
ENSMUSG00000092586	Ly6g6c
ENSMUSG00000092602	4931413I07Rik
ENSMUSG00000092618	Btnl6
ENSMUSG00000092916	Gm24082
ENSMUSG00000093030	Gm23632
ENSMUSG00000093107	Mir1839
ENSMUSG00000093355	Snora26
ENSMUSG00000093394	Gm20621

ENSMUSG00000093484	Gm20657
ENSMUSG00000093511	Gm20680
ENSMUSG00000093537	Gm7584
ENSMUSG00000093553	Gm20633
ENSMUSG00000093575	Gm20695
ENSMUSG00000093577	Gm20632
ENSMUSG00000093606	B130034C11Rik
ENSMUSG00000093619	4930535L15Rik
ENSMUSG00000093656	Gm20628
ENSMUSG00000093682	Gm20681
ENSMUSG00000093760	Gm20678
ENSMUSG00000093834	Rnu1b2
ENSMUSG00000093843	Gm25939
ENSMUSG00000093956	Gm24497
ENSMUSG00000094018	S100a2
ENSMUSG00000094050	Gm23472
ENSMUSG00000094131	Gm22265
ENSMUSG00000094248	Hist1h2ao
ENSMUSG00000094271	Gm13290
ENSMUSG00000094328	Gm9125
ENSMUSG00000094378	Gm15097
ENSMUSG00000094405	Gm23143
ENSMUSG00000094446	Gm6344
ENSMUSG00000094590	Gm10251
ENSMUSG00000094627	Gm10182
ENSMUSG00000094655	Gm25360
ENSMUSG00000094668	Gm24871
ENSMUSG00000094690	1600014C23Rik
ENSMUSG00000094732	1500015L24Rik
ENSMUSG00000094775	Gm2759
ENSMUSG00000094786	Gm14403
ENSMUSG00000094935	Gm9726
ENSMUSG00000094954	Gm8011
ENSMUSG00000095180	Rhox5
ENSMUSG00000095205	Snord93
ENSMUSG00000095369	Gm21859
ENSMUSG00000095384	1700001F09Rik
ENSMUSG00000095395	Gm25617
ENSMUSG00000095419	Gm14328
ENSMUSG00000095538	Gm21983
ENSMUSG00000095551	Gm7980
ENSMUSG00000095580	Rnu1b1
ENSMUSG00000095620	2010005H15Rik
ENSMUSG00000095648	Gm2004
ENSMUSG00000095676	Gm25099
ENSMUSG00000095698	Rhox2d
ENSMUSG00000095701	Gm24830
ENSMUSG00000095718	Gm6502
ENSMUSG00000095738	Gm25313
ENSMUSG00000095789	Nupr1l
ENSMUSG00000095799	Gm8332
ENSMUSG00000095836	Gm21960
ENSMUSG00000095872	Gm15128
ENSMUSG00000095887	Gm10096
ENSMUSG00000095892	Rnu5g
ENSMUSG00000096010	Hist4h4
ENSMUSG00000096024	Gm17174
ENSMUSG00000096035	Gm1045
ENSMUSG00000096052	9930004E17Rik
ENSMUSG00000096065	Gm13155
ENSMUSG00000096094	A630095N17Rik
ENSMUSG00000096215	Gm5480
ENSMUSG00000096276	Gm2042
ENSMUSG00000096280	Gm23287
ENSMUSG00000096351	Samd11
ENSMUSG00000096354	Gm8178
ENSMUSG00000096751	Gm21971
ENSMUSG00000096803	Gm6803
ENSMUSG00000096832	Mir3096
ENSMUSG00000096838	Gm26232
ENSMUSG00000096929	mmu-mir-29b-2
ENSMUSG00000096931	AC102693.1

ENSMUSG00000096932	AC125223.1
ENSMUSG00000096938	AC113484.1
ENSMUSG00000096940	AC110382.1
ENSMUSG00000096956	AC137123.1
ENSMUSG00000096959	AC087541.1
ENSMUSG00000096960	AC133967.1
ENSMUSG00000096964	RP23-124N16.2
ENSMUSG00000096971	AC158921.1
ENSMUSG00000096972	AL929563.1
ENSMUSG00000096979	AL627077.2
ENSMUSG00000096987	AL928605.1
ENSMUSG00000096988	AC122487.1
ENSMUSG00000097019	AL772170.1
ENSMUSG00000097021	AC115880.1
ENSMUSG00000097027	AC170188.1
ENSMUSG00000097035	AC117574.1
ENSMUSG00000097040	AC158956.1
ENSMUSG00000097045	AC087229.1
ENSMUSG00000097055	AC140457.1
ENSMUSG00000097056	AC164093.1
ENSMUSG00000097075	AC122863.1
ENSMUSG00000097092	AC132284.1
ENSMUSG00000097098	AC207128.1
ENSMUSG00000097104	AC132304.1
ENSMUSG00000097107	AC158614.1
ENSMUSG00000097117	AL645861.1
ENSMUSG00000097120	RP24-64D24.2
ENSMUSG00000097123	AL833775.1
ENSMUSG00000097139	AL672278.1
ENSMUSG00000097143	AC140071.1
ENSMUSG00000097146	CT009711.1
ENSMUSG00000097149	AC158993.1
ENSMUSG00000097154	CT009715.1
ENSMUSG00000097157	CT573100.1
ENSMUSG00000097173	AL806526.1
ENSMUSG00000097182	AC163292.1
ENSMUSG00000097184	CT030736.1
ENSMUSG00000097187	RP23-399J8.10
ENSMUSG00000097191	AC122546.1
ENSMUSG00000097193	AC145556.1
ENSMUSG00000097208	AC110377.1
ENSMUSG00000097209	AC115763.1
ENSMUSG00000097233	AC109138.1
ENSMUSG00000097243	AC151836.1
ENSMUSG00000097252	AC105155.1
ENSMUSG00000097259	AC152181.1
ENSMUSG00000097261	AC129195.1
ENSMUSG00000097271	AC132233.1
ENSMUSG00000097274	AC113059.1
ENSMUSG00000097282	AC079441.1
ENSMUSG00000097288	AL683799.1
ENSMUSG00000097289	AL607127.1
ENSMUSG00000097292	CT033785.1
ENSMUSG00000097293	D630002J18Rik
ENSMUSG00000097310	AC163280.1
ENSMUSG00000097317	AL691450.1
ENSMUSG00000097318	AC122247.1
ENSMUSG00000097328	Tnfsf12
ENSMUSG00000097339	AC111080.1
ENSMUSG00000097345	AL732557.1
ENSMUSG00000097352	RP24-547D11.3
ENSMUSG00000097357	AC129082.1
ENSMUSG00000097363	AC126686.1
ENSMUSG00000097372	AC117232.1
ENSMUSG00000097373	AC167669.1
ENSMUSG00000097387	AC105947.1
ENSMUSG00000097402	AC159900.1
ENSMUSG00000097416	WI1-2736D10.2
ENSMUSG00000097432	mmu-mir-1900
ENSMUSG00000097460	AC144860.1
ENSMUSG00000097476	AC160929.1
ENSMUSG00000097489	AC123679.1

ENSMUSG00000097501 AC154861.1
ENSMUSG00000097509 B230322F03Rik
ENSMUSG00000097513 RP23-288L22.3
ENSMUSG00000097516 AC122875.1
ENSMUSG00000097540 AC124108.1
ENSMUSG00000097542 AC098877.1
ENSMUSG00000097543 AC159318.2
ENSMUSG00000097561 AC171500.1
ENSMUSG00000097569 AC153842.1
ENSMUSG00000097573 AC133162.1
ENSMUSG00000097576 AC209577.1
ENSMUSG00000097581 RP24-376E16.2
ENSMUSG00000097584 AC121099.1
ENSMUSG00000097587 AC157610.1
ENSMUSG00000097592 AC164430.1
ENSMUSG00000097598 AC168278.1
ENSMUSG00000097612 RP24-339K18.1
ENSMUSG00000097636 AC123702.1
ENSMUSG00000097639 AC104908.1
ENSMUSG00000097641 AC138768.1
ENSMUSG00000097645 AC122397.1
ENSMUSG00000097648 AC122932.1
ENSMUSG00000097666 AC102195.1
ENSMUSG00000097673 AC156021.1
ENSMUSG00000097693 AC161456.1
ENSMUSG00000097694 AC154781.1
ENSMUSG00000097704 AC153525.1
ENSMUSG00000097709 AC134826.1
ENSMUSG00000097720 AC157950.1
ENSMUSG00000097724 AC116732.1
ENSMUSG00000097727 AC165259.1
ENSMUSG00000097731 AL807833.1
ENSMUSG00000097746 AC124336.1
ENSMUSG00000097747 AL596215.1
ENSMUSG00000097748 AC161511.1
ENSMUSG00000097749 AC119880.1
ENSMUSG00000097757 AC160759.1
ENSMUSG00000097759 CT010433.1
ENSMUSG00000097779 AC123702.2
ENSMUSG00000097785 AC131756.1
ENSMUSG00000097786 AC158135.1
ENSMUSG00000097787 AC115752.1
ENSMUSG00000097791 CT030740.1
ENSMUSG00000097812 RP24-286J21.4
ENSMUSG00000097835 AC108434.1
ENSMUSG00000097838 AL513014.1
ENSMUSG00000097848 AC127583.1
ENSMUSG00000097852 AC155158.1
ENSMUSG00000097854 PPP4R2
ENSMUSG00000097855 AC093339.1
ENSMUSG00000097867 AC116484.1
ENSMUSG00000097873 AL928605.2
ENSMUSG00000097875 AC164883.1
ENSMUSG00000097890 AC118733.2
ENSMUSG00000097933 AC154675.1
ENSMUSG00000097944 RP23-441L19.1
ENSMUSG00000097998 RP23-459L15.5
ENSMUSG00000098041 RP23-474B13.4
ENSMUSG00000098055 RP24-189K8.2
ENSMUSG00000098207 Arl14
ENSMUSG00000098221 RP24-365B9.1
ENSMUSG00000098243 RP23-393I12.2
ENSMUSG00000098410
ENSMUSG00000098434
ENSMUSG00000098449
ENSMUSG00000098465
ENSMUSG00000098626
ENSMUSG00000098843
ENSMUSG00000098943
ENSMUSG00000099002
ENSMUSG00000099032
ENSMUSG00000099107

ENSMUSG00000099133
ENSMUSG00000099139
ENSMUSG00000099145
ENSMUSG00000099291
ENSMUSG00000099338
ENSMUSG00000099375
ENSMUSG00000099413
ENSMUSG00000099422
ENSMUSG00000099465
ENSMUSG00000099472
ENSMUSG00000099528
ENSMUSG00000099540
ENSMUSG00000099553
ENSMUSG00000099556
ENSMUSG00000099583
ENSMUSG00000099590
ENSMUSG00000099600
ENSMUSG00000099609
ENSMUSG00000099632
ENSMUSG00000099655
ENSMUSG00000099804
ENSMUSG00000099825
ENSMUSG00000099876
ENSMUSG00000099906
ENSMUSG00000099928
ENSMUSG00000099930
ENSMUSG00000099937
ENSMUSG00000099966
ENSMUSG00000100005
ENSMUSG00000100009
ENSMUSG00000100017
ENSMUSG00000100094
ENSMUSG00000100096
ENSMUSG00000100119
ENSMUSG00000100199
ENSMUSG00000100247
ENSMUSG00000100287
ENSMUSG00000100306
ENSMUSG00000100505
ENSMUSG00000100556
ENSMUSG00000100625
ENSMUSG00000100632
ENSMUSG00000100666
ENSMUSG00000100680
ENSMUSG00000100769
ENSMUSG00000100774
ENSMUSG00000100779
ENSMUSG00000100816
ENSMUSG00000100829
ENSMUSG00000100865
ENSMUSG00000100876
ENSMUSG00000100975
ENSMUSG00000101037
ENSMUSG00000101082
ENSMUSG00000101169
ENSMUSG00000101225
ENSMUSG00000101234
ENSMUSG00000101268
ENSMUSG00000101316
ENSMUSG00000101362
ENSMUSG00000101394
ENSMUSG00000101400
ENSMUSG00000101406
ENSMUSG00000101549
ENSMUSG00000101555
ENSMUSG00000101588
ENSMUSG00000101609
ENSMUSG00000101641
ENSMUSG00000101750
ENSMUSG00000101791
ENSMUSG00000101807
ENSMUSG00000101856

ENSMUSG00000101904
ENSMUSG00000101932
ENSMUSG00000101942
ENSMUSG00000102059
ENSMUSG00000102086
ENSMUSG00000102123
ENSMUSG00000102151
ENSMUSG00000102158
ENSMUSG00000102169
ENSMUSG00000102189
ENSMUSG00000102200
ENSMUSG00000102201
ENSMUSG00000102204
ENSMUSG00000102263
ENSMUSG00000102268
ENSMUSG00000102269
ENSMUSG00000102271
ENSMUSG00000102278
ENSMUSG00000102297
ENSMUSG00000102344
ENSMUSG00000102380
ENSMUSG00000102385
ENSMUSG00000102398
ENSMUSG00000102418
ENSMUSG00000102425
ENSMUSG00000102428
ENSMUSG00000102448
ENSMUSG00000102457
ENSMUSG00000102482
ENSMUSG00000102545
ENSMUSG00000102562
ENSMUSG00000102590
ENSMUSG00000102591
ENSMUSG00000102593
ENSMUSG00000102652
ENSMUSG00000102700
ENSMUSG00000102731
ENSMUSG00000102759
ENSMUSG00000102763
ENSMUSG00000102775
ENSMUSG00000102781
ENSMUSG00000102798
ENSMUSG00000102802
ENSMUSG00000102808
ENSMUSG00000102819
ENSMUSG00000102830
ENSMUSG00000102833
ENSMUSG00000102881
ENSMUSG00000102890
ENSMUSG00000103006
ENSMUSG00000103032
ENSMUSG00000103041
ENSMUSG00000103067
ENSMUSG00000103082
ENSMUSG00000103084
ENSMUSG00000103103
ENSMUSG00000103108
ENSMUSG00000103156
ENSMUSG00000103160
ENSMUSG00000103170
ENSMUSG00000103182
ENSMUSG00000103187
ENSMUSG00000103189
ENSMUSG00000103280
ENSMUSG00000103309
ENSMUSG00000103373
ENSMUSG00000103376
ENSMUSG00000103385
ENSMUSG00000103406
ENSMUSG00000103408
ENSMUSG00000103427
ENSMUSG00000103440

ENSMUSG00000103454
ENSMUSG00000103475
ENSMUSG00000103477
ENSMUSG00000103502
ENSMUSG00000103508
ENSMUSG00000103520
ENSMUSG00000103562
ENSMUSG00000103593
ENSMUSG00000103613
ENSMUSG00000103620
ENSMUSG00000103622
ENSMUSG00000103629
ENSMUSG00000103662
ENSMUSG00000103701
ENSMUSG00000103718
ENSMUSG00000103726
ENSMUSG00000103751
ENSMUSG00000103811
ENSMUSG00000103827
ENSMUSG00000103866
ENSMUSG00000103925
ENSMUSG00000103940
ENSMUSG00000103966
ENSMUSG00000103988
ENSMUSG00000104025
ENSMUSG00000104042
ENSMUSG00000104063
ENSMUSG00000104090
ENSMUSG00000104093
ENSMUSG00000104116
ENSMUSG00000104122
ENSMUSG00000104141
ENSMUSG00000104165
ENSMUSG00000104168
ENSMUSG00000104186
ENSMUSG00000104187
ENSMUSG00000104190
ENSMUSG00000104208
ENSMUSG00000104235
ENSMUSG00000104238
ENSMUSG00000104338
ENSMUSG00000104350
ENSMUSG00000104361
ENSMUSG00000104379
ENSMUSG00000104419
