# Supplementary material for: Computational Identification of Key Regulators in Two Different Colorectal Cancer Cell Lines
Source: Front Genet. 2016 Apr 5;7:42. doi: 10.3389/fgene.2016.00042 (PMC4820448; doi:10.3389/fgene.2016.00042)
Supplement: Supplementary Table S3 — Colorectal cancer-related non-redundant PWM library. [file Table3.PDF]

Table S3. This library contains 229 colorectal cancer-related non-redundant position weight matrices (PWMs) based on the TRANSFAC database (release 2014.1) to minimize the rate of false predictions in promoter analyses. Using the F-MATCH program of the geneXplain platform, this library was used to predict enriched transcription factor binding sites in the promoter sequences (-1000 to 100 bp regions relative to transcription start sites) of the signature genes of the colorectal cell lines 1638N-T1 and CMT-93.

ID
V$AHRARNT_01
V$AHRARNT_02
V$AHRHIF_Q6
V$ALPHACP1_01
V$ALX4_01
V$AML1_01
V$AML1_Q6
V$AML_Q6
V$AP1FJ_Q2
V$AP1_01
V$AP1_Q2_01
V$AP1_Q6_01
V$AP2ALPHA_01
V$AP2ALPHA_02
V$AP2_Q3
V$AP2_Q6
V$AP2_Q6_01
V$AREB6_01
V$AREB6_02
V$AREB6_03
V$AREB6_04
V$ARNT_01
V$ARNT_02
V$AR_01
V$AR_02
V$AR_Q2
V$AR_Q6_01
V$ATF3_Q6
V$ATF4_Q2
V$BETACATENIN_Q6
V$BRACH_01
V$BRCA_01
V$CACCCBINDINGFACTOR_Q6
V$CDPCR1_01
V$CDPCR3HD_01
V$CDPCR3_01
V$CDP_01
V$CDP_02
V$CDX2_Q5
V$CDX_Q5
V$CEBPB_01
V$CEBPB_02
V$CEBP_Q3
V$CETS1P54_01
V$CETS1P54_02
V$CHOP_01
V$CLOCKBMAL_Q6
V$CMYB_01
V$COUP_01
V$CREBP1CJUN_01
V$CREB_Q3
V$CREL_01
V$DEAF1_01
V$DEAF1_02
V$DEC_Q1
V$DELTAEF1_01
V$DR1_Q3
V$DR3_Q4
V$DR4_Q2
V$E12_Q6
V$E2F1DP1_01
V$E2F1DP2_01
V$E2F1_Q3
V$E2F1_Q3_01

V$E2F1_Q4
V$E2F4DP1_01
V$E2F_01
V$E2F_02
V$E2F_03
V$E2F_Q2
V$E2F_Q3_01
V$E2F_Q4_01
V$E47_01
V$E47_02
V$EBF_Q6
V$EBOX_Q6_01
V$EFC_Q6
V$EGR1_01
V$EGR_Q6
V$EN1_01
V$ER_Q6
V$ETS1_B
V$ETS_Q4
V$FOXM1_01
V$FOXO1_02
V$FOXO3_01
V$FOXP3_Q4
V$FOX_Q2
V$FXR_Q3
V$GATA4_Q3
V$GATA6_01
V$GLI_Q2
V$HAND1E47_01
V$HEB_Q6
V$HES1_Q2
V$HFH8_01
V$HIC1_02
V$HIC1_03
V$HIF1_Q5
V$HMGIY_Q3
V$HMGIY_Q6
V$HNF1_01
V$HNF1_C
V$HNF1_Q6
V$HNF3ALPHA_Q6
V$HNF3B_01
V$HNF3_Q6
V$HNF4ALPHA_Q6
V$HNF4_01
V$HNF4_01_B
V$HNF4_Q6_01
V$HNF6_Q6
V$HOX13_01
V$HSF1_01
V$HSF1_Q6
V$HSF_Q6
V$IK1_01
V$IK2_01
V$IK3_01
V$IPF1_Q4
V$IPF1_Q4_01
V$IRF_Q6
V$KAISO_01
V$KROX_Q6
V$LEF1_Q2
V$LXR_Q3
V$LYF1_01
V$MAF_Q6_01
V$MEIS1AHOXA9_01
V$MEIS1BHOXA9_02
V$MYB_Q3
V$MYB_Q6
V$MYCMAX_01
V$MYCMAX_02
V$MYCMAX_B
V$MYC_Q2

V$MYOD_01
V$MYOD_Q6
V$MYOD_Q6_01
V$MZF1_01
V$MZF1_02
V$NANOG_01
V$NFAT_Q6
V$NFKAPPAB50_01
V$NFKAPPAB65_01
V$NFKAPPAB_01
V$NFKB_C
V$NFKB_Q6
V$NFKB_Q6_01
V$NFY_01
V$NFY_C
V$NFY_Q6
V$NRF2_Q4
V$NRSE_B
V$NRSF_01
V$NRSF_Q4
V$OCT4_01
V$OCT4_02
V$OCT_Q6
V$OSF2_Q6
V$OTX_Q1
V$P300_01
V$P53_01
V$P53_02
V$PAX2_01
V$PAX2_02
V$PAX3_01
V$PAX3_B
V$PAX6_01
V$PAX6_Q2
V$PAX_Q6
V$PBX_Q3
V$PPARA_02
V$PPARG_01
V$PPARG_02
V$PPARG_03
V$RBPJK_01
V$RBPJK_Q4
V$RFX_Q6
V$RORA1_01
V$RORA2_01
V$RUSH1A_02
V$SMAD3_Q6
V$SMAD4_Q6
V$SMAD_Q6
V$SMAD_Q6_01
V$SOX9_B1
V$SOX_Q6
V$SP1_01
V$SP1_Q4_01
V$SP1_Q6
V$SP1_Q6_01
V$SP3_Q3
V$SREBP1_01
V$SREBP1_02
V$SREBP1_Q6
V$SREBP_Q3
V$SRF_01
V$SRF_C
V$SRF_Q4
V$SRF_Q5_01
V$SRF_Q6
V$STAT1_01
V$STAT3_01
V$STAT5A_01
V$STAT5A_02
V$STAT_01
V$STAT_Q6

V$TAL1BETAE47_01
V$TATA_01
V$TATA_C
V$TBP_01
V$TBP_Q6
V$TBX5_01
V$TBX5_02
V$TBX5_Q5
V$TCF4_Q5
V$TEF1_Q6
V$TFE_Q6
V$USF2_Q6
V$USF_01
V$USF_02
V$USF_C
V$USF_Q6
V$USF_Q6_01
V$VDR_Q3
V$VDR_Q6_01
V$WT1_Q6
V$ZIC1_01
