# Supplementary material for: Computational Identification of Key Regulators in Two Different Colorectal Cancer Cell Lines
Source: Front Genet. 2016 Apr 5;7:42. doi: 10.3389/fgene.2016.00042 (PMC4820448; doi:10.3389/fgene.2016.00042)
Supplement: Supplementary Table S4 — 1638N-T1-specific TF set. [file Table4.PDF]

Table S4. Using the geneXplain platform and a colorectal cancer-specific PWM library (Table S3), the signature genes from the Tables S1 and S2 were subjected as foreground sets to search for enriched TFBSs in their promoter regions. For each foreground set, a list of significant enriched TFBSs was generated- one for each cell line.
The TFBSs were then mapped to their corresponding TFs, and subsequently, the TFs were intersected. This table contains the transcription factors which were only found for 1638N-T1, but not for CMT-93.

| Gene description | Gene symbol | Site model ID |
|---|---|---|
| SRY-box containing gene 9 | Sox9 | V$SOX_Q6 |
| helicase-like transcription factor | Hltf | V$RUSH1A_02 |
| interferon regulatory factor 3 | Irf3 | V$IRF_Q6 |
| GATA binding protein 6 | Gata6 | V$GATA6_01 |
| catenin (cadherin associated protein), beta 1 | Ctnnb1 | V$BETACATENIN_Q6 |
| TATA box binding protein | Tbp | V$TATA_C |
| interferon regulatory factor 1 | Irf1 | V$IRF_Q6 |
| Meis homeobox 1 | Meis1 | V$MEIS1BHOXA9_02 |
| HNF1 homeobox B | Hnf1b | V$HNF1_Q6 |
| interferon regulatory factor 4 | Irf4 | V$IRF_Q6 |
| heat shock factor 1 | Hsf1 | V$HSF1_01 |
| nuclear factor of activated T cells, cytoplasmic, calcineurin dependent 4 | Nfatc4 | V$NFAT_Q6 |
| SRY-box containing gene 8 | Sox8 | V$SOX_Q6 |
| caudal type homeobox 1 | Cdx1 | V$CDX_Q5 |
| interferon regulatory factor 7 | Irf7 | V$IRF_Q6 |
| interferon regulatory factor 6 | Irf6 | V$IRF_Q6 |
| nuclear factor of activated T cells, cytoplasmic, calcineurin dependent 2 | Nfatc2 | V$NFAT_Q6 |
| HNF1 homeobox A | Hnf1a | V$HNF1_C |
| pancreatic and duodenal homeobox 1 | Pdx1 | V$IPF1_Q4 |
| caudal type homeobox 2 | Cdx2 | V$CDX2_Q5 |
| interferon regulatory factor 5 | Irf5 | V$IRF_Q6 |
| interferon regulatory factor 2 | Irf2 | V$IRF_Q6 |
| SRY-box containing gene 10 | Sox10 | V$SOX_Q6 |
| forkhead box H1 | Foxh1 | V$FOX_Q2 |
| forkhead box J1 | Foxj1 | V$FOX_Q2 |
| forkhead box A1 | Foxa1 | V$HNF3ALPHA_Q6 |
| forkhead box A2 | Foxa2 | V$HNF3B_01 |
| homeobox A9 | Hoxa9 | V$MEIS1BHOXA9_02 |
| homeobox A5 | Hoxa5 | V$HOX13_01 |
| forkhead box F2 | Foxf2 | V$FOX_Q2 |
| forkhead box P3 | Foxp3 | V$FOXP3_Q4 |
| aristaless-like homeobox 4 | Alx4 | V$ALX4_01 |
| forkhead box A3 | Foxa3 | V$HNF3_Q6 |
| SRY-box containing gene 15 | Sox15 | V$SOX_Q6 |
| interferon regulatory factor 8 | Irf8 | V$IRF_Q6 |
| SRY-box containing gene 5 | Sox5 | V$SOX_Q6 |
| forkhead box F1 | Foxf1 | V$HFH8_01 |
| one cut domain, family member 1 | Onecut1 | V$HNF6_Q6 |
| one cut domain, family member 2 | Onecut2 | V$HNF6_Q6 |
| SRY-box containing gene 18 | Sox18 | V$SOX_Q6 |
| high mobility group AT-hook 1 | Hmga1 | V$HMGIY_Q3 |
| nuclear receptor subfamily 1, group H, member 4 | Nr1h4 | V$FXR_Q3 |
| forkhead box O3 | Foxo3 | V$FOXO3_01 |
| SRY-box containing gene 6 | Sox6 | V$SOX_Q6 |
| high mobility group AT-hook 2 | Hmga2 | V$HMGIY_Q3 |
| SRY-box containing gene 11 | Sox11 | V$SOX_Q6 |
| forkhead box D3 | Foxd3 | V$FOX_Q2 |
| sex determining region of Chr Y | Sry | V$SOX_Q6 |
| SRY-box containing gene 13 | Sox13 | V$SOX_Q6 |
| SRY-box containing gene 4 | Sox4 | V$SOX_Q6 |
| high mobility group AT-hook I, related sequence 1 | Hmga1-rs1 | V$HMGIY_Q3 |
