# Supplementary material for: Computational Identification of Key Regulators in Two Different Colorectal Cancer Cell Lines
Source: Front Genet. 2016 Apr 5;7:42. doi: 10.3389/fgene.2016.00042 (PMC4820448; doi:10.3389/fgene.2016.00042)
Supplement: Supplementary Table S5 — CMT-93-specific TF set. [file Table5.PDF]

Table S4. Table S4. Using the geneXplain platform and a colorectal cancer-specific PWM library (Table S3), the signature genes from the Tables S1 and S2 were subjected as foreground sets to search for enriched TFBSs in their promoter regions. For each foreground set, a list of significant enriched TFBSs was generated- one for each cell line.
The TFBSs were then mapped to their corresponding TFs, and subsequently, the TFs were intersected. This table contains the transcription factors which were only found for CMT-93, but not for 1638N-T1.

Gene description	Gene symbol	Site model ID
signal transducer and activator of transcription 6	Stat6	V$STAT_Q6
signal transducer and activator of transcription 3	Stat3	V$STAT_Q6
signal transducer and activator of transcription 5A	Stat5a	V$STAT_Q6
paired box gene 2	Pax2	V$PAX2_01
ELK1, member of ETS oncogene family	Elk1	V$ETS_Q4
early B cell factor 3	Ebf3	V$EBF_Q6
Friend leukemia integration 1	Fli1	V$ETS_Q4
sterol regulatory element binding transcription factor 1	Srebf1	V$SREBP1_02
signal transducer and activator of transcription 5B	Stat5b	V$STAT_Q6
GLI-Kruppel family member GLI3	Gli3	V$GLI_Q2
transcription factor AP-2, alpha	Tfap2a	V$AP2ALPHA_01
early B cell factor 2	Ebf2	V$EBF_Q6
sterol regulatory element binding factor 2	Srebf2	V$SREBP_Q3
zinc finger protein 148	Zfp148	V$CACCCBINDINGFACTOR_Q6
E26 avian leukemia oncogene 2, 3' domain	Ets2	V$ETS_Q4
zinc finger E-box binding homeobox 1	Zeb1	V$AREB6_01
v-rel reticuloendotheliosis viral oncogene homolog A (avian)	Rela	V$NFKB_Q6
GLI-Kruppel family member GLI1	Gli1	V$GLI_Q2
ELK4, member of ETS oncogene family	Elk4	V$ETS_Q4
activating transcription factor 3	Atf3	V$ATF3_Q6
trans-acting transcription factor 3	Sp3	V$SP3_Q3
nuclear factor of kappa light polypeptide gene enhancer in B cells 1, p105	Nfkb1	V$NFKB_Q6
fos-like antigen 2	Fosl2	V$AP1_Q6_01
RE1-silencing transcription factor	Rest	V$NRSF_01
E26 avian leukemia oncogene 1, 5' domain	Ets1	V$ETS_Q4
GLIS family zinc finger 1	Glis1	V$GLI_Q2
E74-like factor 1	Elf1	V$ETS_Q4
E74-like factor 2	Elf2	V$ETS_Q4
avian erythroblastosis virus E-26 (v-ets) oncogene related	Erg	V$ETS_Q4
Ets2 repressor factor	Erf	V$ETS_Q4
GLI-Kruppel family member GLI2	Gli2	V$GLI_Q2
early B cell factor 1	Ebf1	V$EBF_Q6
transformation related protein 53	Trp53	V$P53_02
