# Supplementary material for: Computational Identification of Key Regulators in Two Different Colorectal Cancer Cell Lines
Source: Front Genet. 2016 Apr 5;7:42. doi: 10.3389/fgene.2016.00042 (PMC4820448; doi:10.3389/fgene.2016.00042)
Supplement: Supplementary Table S6 — Intersection-specific TF set between 1638N-T1 and CMT-93. [file Table6.PDF]

Table S6. Table S4. Using the geneXplain platform and a colorectal cancer-specific PWM library (Table S3), the signature genes from the Tables S1 and S2 were subjected as foreground sets to search for enriched TFBSs in their promoter regions. For each foreground set, a list of significant enriched TFBSs was generated- one for each cell line. The TFBSs were then mapped to their corresponding TFs, and subsequently, the TFs were intersected. This table contains the intersection-specific transcription factors between both cell lines.

Gene description	Gene symbol	Site model ID
transcription factor E3	Tfe3	V$TFE_Q6
myogenic factor 5	Myf5	V$EBOX_Q6_01
peroxisome proliferator activated receptor gamma	Pparg	V$DR1_Q3
FBJ osteosarcoma oncogene B	Fosb	V$AP1_01
nuclear receptor subfamily 1, group I, member 3	Nr1i3	V$DR3_Q4
Pbx/knotted 1 homeobox	Pknox1	V$PBX_Q3
POU domain, class 2, transcription factor 2	Pou2f2	V$OCT_Q6
myogenic differentiation 1	Myod1	V$MYOD_Q6_01
Nanog homeobox	Nanog	V$NANOG_01
serum response factor	Srf	V$SRF_01
nuclear factor, erythroid derived 2, like 2	Nfe2l2	V$MAF_Q6_01
retinoid X receptor alpha	Rxra	V$DR1_Q3
hepatocyte nuclear factor 4, gamma	Hnf4g	V$DR1_Q3
hepatic nuclear factor 4, alpha	Hnf4a	V$DR1_Q3
v-maf musculoaponeurotic fibrosarcoma oncogene family, protein K (avian)	Mafk	V$MAF_Q6_01
T-box 5	Tbx5	V$TBX5_Q5
achaete-scute complex homolog 1 (Drosophila)	Ascl1	V$EBOX_Q6_01
transcription factor 3	Tcf3	V$TAL1BETAE47_01
FBJ osteosarcoma oncogene	Fos	V$AP1_01
Max dimerization protein 3	Mxd3	V$EBOX_Q6_01
SMAD family member 5	Smad5	V$SMAD_Q6_01
myelocytomatosis oncogene	Myc	V$EBOX_Q6_01
peroxisome proliferator activated receptor alpha	Ppara	V$DR1_Q3
vitamin D receptor	Vdr	V$DR3_Q4
nuclear receptor subfamily 1, group I, member 2	Nr1i2	V$DR3_Q4
transcription factor EB	Tfeb	V$TFE_Q6
POU domain, class 5, transcription factor 1	Pou5f1	V$OCT4_01
SMAD family member 4	Smad4	V$SMAD_Q6_01
SMAD family member 2	Smad2	V$SMAD_Q6_01
fos-like antigen 1	Fosl1	V$AP1_01
Max interacting protein 1	Mxi1	V$EBOX_Q6_01
BTB and CNC homology 1	Bach1	V$MAF_Q6_01
SMAD family member 7	Smad7	V$SMAD_Q6_01
myogenin	Myog	V$MYOD_Q6_01
POU domain, class 2, transcription factor 1	Pou2f1	V$OCT_Q6
upstream transcription factor 1	Usf1	V$EBOX_Q6_01
SMAD family member 9	Smad9	V$SMAD_Q6_01
T cell acute lymphocytic leukemia 2	Tal2	V$EBOX_Q6_01
T cell acute lymphocytic leukemia 1	Tal1	V$TAL1BETAE47_01
transcription factor EC	Tfec	V$TFE_Q6
nuclear factor, erythroid derived 2, like 3	Nfe2l3	V$MAF_Q6_01
basic helix-loop-helix family, member e40	Bhlhe40	V$EBOX_Q6_01
basic helix-loop-helix family, member e41	Bhlhe41	V$EBOX_Q6_01
nuclear receptor subfamily 2, group F, member 2	Nr2f2	V$DR1_Q3
SMAD family member 1	Smad1	V$SMAD_Q6_01
core binding factor beta	Cbfb	V$AML_Q6
POU domain, class 2, associating factor 1	Pou2af1	V$OCT_Q6
transcription factor 12	Tcf12	V$EBOX_Q6_01
SMAD family member 3	Smad3	V$SMAD3_Q6
pre B cell leukemia homeobox 2	Pbx2	V$PBX_Q3
CCAAT/enhancer binding protein (C/EBP), alpha	Cebpa	V$CEBP_Q3
microphthalmia-associated transcription factor	Mitf	V$TFE_Q6
myogenic factor 6	Myf6	V$MYOD_Q6_01
SMAD family member 6	Smad6	V$SMAD_Q6_01
v-myc myelocytomatosis viral related oncogene, neuroblastoma derived (avian)	Mycn	V$EBOX_Q6_01
heart and neural crest derivatives expressed transcript 1	Hand1	V$EBOX_Q6_01
heart and neural crest derivatives expressed transcript 2	Hand2	V$EBOX_Q6_01
pre B cell leukemia homeobox 3	Pbx3	V$PBX_Q3
runt related transcription factor 2	Runx2	V$OSF2_Q6
retinoid X receptor beta	Rxrb	V$DR3_Q4
BTB and CNC homology 2	Bach2	V$MAF_Q6_01
v-maf musculoaponeurotic fibrosarcoma oncogene family, protein F (avian)	Maff	V$MAF_Q6_01
POU domain, class 3, transcription factor 3	Pou3f3	V$OCT_Q6
zinc finger and BTB domain containing 33	Zbtb33	V$KAISO_01

```
POU domain, class 4, transcription factor 1     Pou4f1  V$OCT_Q6
nescient helix loop helix 1     Nhlh1   V$EBOX_Q6_01
v-maf musculoaponeurotic fibrosarcoma oncogene family, protein G (avian)        Mafg    V$MAF_Q6_01
CCAAT/enhancer binding protein (C/EBP), epsilon Cebpe   V$CEBP_Q3
pre B cell leukemia homeobox 1  Pbx1    V$PBX_Q3
Jun oncogene    Jun     V$AP1_01
Jun-B oncogene  Junb    V$AP1_01
transcription factor 4  Tcf4    V$EBOX_Q6_01
TEA domain family member 1      Tead1   V$TEF1_Q6
avian musculoaponeurotic fibrosarcoma (v-maf) AS42 oncogene homolog     Maf     V$MAF_Q6_01
CCAAT/enhancer binding protein (C/EBP), gamma   Cebpg   V$CEBP_Q3
CCAAT/enhancer binding protein (C/EBP), beta    Cebpb   V$CEBPB_02
POU domain, class 3, transcription factor 4     Pou3f4  V$OCT_Q6
upstream transcription factor 2 Usf2    V$EBOX_Q6_01
nuclear factor, erythroid derived 2     Nfe2    V$MAF_Q6_01
runt related transcription factor 3     Runx3   V$AML_Q6
Jun proto-oncogene related gene d       Jund    V$AP1_01
v-maf musculoaponeurotic fibrosarcoma oncogene family, protein B (avian)        Mafb    V$MAF_Q6_01
POU domain, class 3, transcription factor 1     Pou3f1  V$OCT_Q6
POU domain, class 3, transcription factor 2     Pou3f2  V$OCT_Q6
```
