# Supplementary material for: Computational Identification of Key Regulators in Two Different Colorectal Cancer Cell Lines
Source: Front Genet. 2016 Apr 5;7:42. doi: 10.3389/fgene.2016.00042 (PMC4820448; doi:10.3389/fgene.2016.00042)
Supplement: Supplementary Table S7 — Master regulatory network based on the intersection-specific TF set in Pair Graph File format. [file Table7.PDF]

Table S7A. This Pair Graph File contains the pathway reactions including the TRANSPATH molecule IDs for the master regulatory network based on the intersection-specific TF set (Figure 2). Figure S7B depicts the corresponding network as a Scalable Vector Graphics (SVG) image. Please see Figure 2 for the image description.

```
//XN000000746: Cdc25B -> Cdk1
MO000137154     XN000000746
XN000000746     MO000087611

//XN000001626: PDK1 -> PKACA-isoform1
MO000059185     XN000001626
XN000001626     MO000102335

//XN000001668: PDK1 -> PKCbeta
MO000059185     XN000001668
XN000001668     MO000086659

//XN000001857: SARA -> Smad5 + Smad8
MO000083057     XN000001857
XN000001857     MO000081400
XN000001857     MO000137808

//XN000001897: RhoA -> FAK1-isoform3
MO000087899     XN000001897
XN000001897     MO000058753

//XN000001977: BMPR-IA -> Smad5
MO000134939     XN000001977
XN000001977     MO000081400

//XN000002017: Smad7-A -> BMPR-IB
MO000058700     XN000002017
XN000002017     MO000081402

//XN000002080: JNK1 -> JunB
MO000057427     XN000002080
XN000002080     MO000078297

//XN000002135: RhoA -> p38alpha-isoform1
MO000087899     XN000002135
XN000002135     MO000059917

//XN000003320: Smad3 -> Smad4
MO000082956     XN000003320
XN000003320     MO000088883

//XN000003450: AKT-1 -> IKK-alpha-isoform1
MO000057082     XN000003450
XN000003450     MO000090848

//XN000003650: ERK2 -> Tal-2
MO000056722     XN000003650
XN000003650     MO000095519

//XN000003676: Abl-isoform1 -> PDK1
MO000060201     XN000003676
XN000003676     MO000059185

//XN000004136:  -Smad6-> Smad2 + Smad2-Short
XN000004136     MO000083052
XN000004136     MO000257487
MO000020401     XN000004136

//XN000004139:  -Smad6-> Smad1
XN000004139     MO000081398
MO000020401     XN000004139

//XN000004682: Smad4 + Smad8 -BMPR-IA->
MO000088883     XN000004682
MO000137808     XN000004682
MO000134939     XN000004682
```

```
//XN000004683: Smad8 -BMPR-IB->
MO000137808     XN000004683
MO000081402     XN000004683

//XN000004705:  -Smad7-A-> Smad1
XN000004705     MO000081398
MO000058700     XN000004705

//XN000005174: PKACA-isoform1 -> (PKAr)2:(PKAc)2
MO000102335     XN000005174
XN000005174     MO000058482

//XN000006877:  -Cdc25B-> Cdk2-alpha
XN000006877     MO000023199
MO000137154     XN000006877

//XN000011638:  -p53as-> mdm2-isoform1
XN000011638     MO000078228
MO000026166     XN000011638

//XN000011691: MyoD -Cdk2-alpha->
MO000084139     XN000011691
MO000023199     XN000011691

//XN000014588: MITF-M + MITF-A + MITF-A1 + MITF-H + MITF-H1 + MITF-H2 + MITF-H3 + MITF-M1 + MITF-A2 -
RSK1->
MO000086132     XN000014588
MO000086136     XN000014588
MO000086138     XN000014588
MO000086140     XN000014588
MO000086143     XN000014588
MO000086144     XN000014588
MO000086145     XN000014588
MO000086149     XN000014588
MO000172372     XN000014588
MO000059507     XN000014588

//XN000015258: HNF-4alpha2 + HNF-4alpha-Short + HNF-4alpha-Long -PKACA-isoform1->
MO000026409     XN000015258
MO000255545     XN000015258
MO000313395     XN000015258
MO000102335     XN000015258

//XN000015281: PKACA-isoform1 -> HNF-4gamma
MO000102335     XN000015281
XN000015281     MO000150458

//XN000015286: CBP -> HNF-4gamma
MO000059021     XN000015286
XN000015286     MO000150458

//XN000015411: PPARgamma1 -ERK2->
MO000026509     XN000015411
MO000023651     XN000015411

//XN000015485: PPARalpha -PKACA-isoform1->
MO000057686     XN000015485
MO000102335     XN000015485

//XN000015505: p300 -> PPARgamma1 + PPARgamma2
MO000056523     XN000015505
XN000015505     MO000026509
XN000015505     MO000033685

//XN000015559: p300 -> c-Fos
MO000056523     XN000015559
XN000015559     MO000060047

//XN000016564:  -PRK2-isoform1-> AKT-1
XN000016564     MO000057082
MO000103817     XN000016564
```

```
//XN000016662: RhoA -> PRK2-isoform1
MO000087899     XN000016662
XN000016662     MO000103817

//XN000016736: C/EBPalpha -GSK3beta->
MO000002634     XN000016736
MO000057073     XN000016736

//XN000017615: Rad23A -> p300
MO000108601     XN000017615
XN000017615     MO000056523

//XN000017893: RhoA -> ROCK-II
MO000087899     XN000017893
XN000017893     MO000090320

//XN000021573: MAPKAPK2 -p38alpha-isoform1->
MO000123092     XN000021573
MO000059917     XN000021573

//XN000021745: MITF-M + MITF-A + MITF-A1 + MITF-H + MITF-H1 + MITF-H2 + MITF-H3 + MITF-M1 + MITF-A2 -
p38alpha-isoform1->
MO000086132     XN000021745
MO000086136     XN000021745
MO000086138     XN000021745
MO000086140     XN000021745
MO000086143     XN000021745
MO000086144     XN000021745
MO000086145     XN000021745
MO000086149     XN000021745
MO000172372     XN000021745
MO000059917     XN000021745

//XN000021818: SRC-1-isoform2 -> CAR2 + CAR
MO000084278     XN000021818
XN000021818     MO000026729
XN000021818     MO000056528

//XN000023759: C/EBPalpha(p30) + C/EBPalpha(p20) -GSK3beta->
MO000026105     XN000023759
MO000026349     XN000023759
MO000057073     XN000023759

//XN000024153: Smad3 -> HRS:PtdIns(3)P:SARA:Smad3
MO000082956     XN000024153
XN000024153     MO000039026

//XN000024154: HRS:PtdIns(3)P:SARA:Smad3 -> SARA
MO000039026     XN000024154
XN000024154     MO000083057

//XN000024967:  -AKT-1-> MKK7beta1 + JNK1
XN000024967     MO000041368
XN000024967     MO000057427
MO000057082     XN000024967

//XN000025584: p38alpha-isoform1 -> MRF4
MO000059917     XN000025584
XN000025584     MO000025749

//XN000025587: MRF4 -p38alpha->
MO000025749     XN000025587
MO000022214     XN000025587

//XN000033754: c-Fos -RSK1->
MO000060047     XN000033754
MO000059507     XN000033754

//XN000042655: E1 -Cdk1->
MO000081629     XN000042655
MO000087611     XN000042655
```

```
//XN000045387: HNF-4alpha2 + HNF-4alpha-Short + HNF-4alpha-Long -CBP->
MO000026409     XN000045387
MO000255545     XN000045387
MO000313395     XN000045387
MO000059021     XN000045387

//XN000046042: IKK-alpha-isoform1 -AKT-2->
MO000090848     XN000046042
MO000057079     XN000046042

//XN000049332: p300 -> myogenin
MO000056523     XN000049332
XN000049332     MO000080384

//XN000049334: p300 -> NF-E2 p45
MO000056523     XN000049334
XN000049334     MO000097606

//XN000049341: p300 -> Oct3-xbb1
MO000056523     XN000049341
XN000049341     MO000093729

//XN000049356: p300 -> USF2a + usf1 + USF2b
MO000056523     XN000049356
XN000049356     MO000056615
XN000049356     MO000084434
XN000049356     MO000116400

//XN000049379: p38alpha-isoform1 -MKK7beta1->
MO000059917     XN000049379
MO000041368     XN000049379

//XN000049975: PKC-delta-I -PDK1->
MO000057524     XN000049975
MO000059185     XN000049975

//XN000050187: POU2F1b + POU2F1c + POU2F1a + POU2F1-isoform4 + POU2F1-isoform5 + POU2F1-isoform6 +
POU2F1-isoform7 + POU2F1-isoform8 + POU2F1-isoform9 + POU2F1-isoform10 + POU2F1-isoform11 + POU2F1-
isoform12 -DNA-PKcs->
MO000025984     XN000050187
MO000025985     XN000050187
MO000046030     XN000050187
MO000256865     XN000050187
MO000256866     XN000050187
MO000256867     XN000050187
MO000256868     XN000050187
MO000256869     XN000050187
MO000256870     XN000050187
MO000259088     XN000050187
MO000259089     XN000050187
MO000259090     XN000050187
MO000084852     XN000050187

//XN000050881: Rad23A -> CBP
MO000108601     XN000050881
XN000050881     MO000059021

//XN000051539: RSK2 -PDK1->
MO000084411     XN000051539
MO000059185     XN000051539

//XN000052133: Smad3 -> AML3 + PEBP2alphaA2 + AML3-isoform2 + AML3-G1 + AML3-U1 + AML3-Y1 + AML3-
isoform1 + AML3-G2 + AML3-Y2
MO000082956     XN000052133
XN000052133     MO000025371
XN000052133     MO000025372
XN000052133     MO000026272
XN000052133     MO000026315
XN000052133     MO000026316
XN000052133     MO000026317
XN000052133     MO000089847
XN000052133     MO000089850
```

```
XN000052133     MO000089851

//XN000052135: Smad3 -> C/EBPbeta-FL + C/EBPbeta-LIP + C/EBPbeta-isoform2 + C/EBPbeta-isoform3
MO000082956     XN000052135
XN000052135     MO000002639
XN000052135     MO000026350
XN000052135     MO000327631
XN000052135     MO000327632

//XN000052160: Smad3 -> proCaspase-3
MO000082956     XN000052160
XN000052160     MO000059519

//XN000052169: Smad3 -> VDR
MO000082956     XN000052169
XN000052169     MO000088509

//XN000053333: Tal-1 -p300->
MO000025936     XN000053333
MO000056523     XN000053333

//XN000056066: ERK2 -> JunD
MO000056722     XN000056066
XN000056066     MO000060057

//XN000056676: Melk -> Cdc25B
MO000236765     XN000056676
XN000056676     MO000137154

//XN000057131: proCaspase-3 -> DNA-PKcs
MO000059519     XN000057131
XN000057131     MO000084852

//XN000057760: sumo1 -> C/EBPepsilon
MO000080909     XN000057760
XN000057760     MO000155634

//XN000060711: MafG -CBP->
MO000096278     XN000060711
MO000059021     XN000060711

//XN000061400: RXR-alpha -ERK1->
MO000056800     XN000061400
MO000056725     XN000061400

//XN000061431: RXR-alpha -JNK1->
MO000056800     XN000061431
MO000057427     XN000061431

//XN000061753: ERK2 + ERK1 + ERK5-isoform1 -MEK1-> ERK2
MO000056722     XN000061753
MO000056725     XN000061753
MO000082097     XN000061753
XN000061753     MO000023651
MO000060055     XN000061753

//XN000062867: C/EBPalpha(p30) + C/EBPalpha(p20) -ERK2->
MO000026105     XN000062867
MO000026349     XN000062867
MO000056722     XN000062867

//XN000063379: MEK1 -Raf-1-isoform1->
MO000060055     XN000063379
MO000058621     XN000063379

//XN000063381: MEK1 -PDK1->
MO000060055     XN000063381
MO000059185     XN000063381

//XN000064003: E12 + E47 -ERK2->
MO000025923     XN000064003
MO000025925     XN000064003
```

```
MO000056722     XN000064003

//XN000083963: USF2a + usf1 + USF2b -p38alpha-isoform1->
MO000056615     XN000083963
MO000084434     XN000083963
MO000116400     XN000083963
MO000059917     XN000083963

//XN000086293: Smad3 -> SRF-L
MO000082956     XN000086293
XN000086293     MO000025159

//XN000087214: Fra-1 -ERK5-isoform1->
MO000060043     XN000087214
MO000082097     XN000087214

//XN000090571: SRF-L -PKC-delta-I->
MO000025159     XN000090571
MO000057524     XN000090571

//XN000091701: GCN5 -DNA-PKcs->
MO000086602     XN000091701
MO000084852     XN000091701

//XN000091706: p300 -> MASH-1
MO000056523     XN000091706
XN000091706     MO000086288

//XN000091815: DNA-PKcs -PARP-long->
MO000084852     XN000091815
MO000088856     XN000091815

//XN000093242: RSK1 -PDK1->
MO000059507     XN000093242
MO000059185     XN000093242

//XN000094418: Raf-1-isoform1 -AKT-1->
MO000058621     XN000094418
MO000057082     XN000094418

//XN000097512: JunB -itch-xbb1->
MO000078297     XN000097512
MO000082501     XN000097512

//XN000097642: Smad3 -> itch-xbb1
MO000082956     XN000097642
XN000097642     MO000082501

//XN000103046: p/CAF -mdm2-isoform1->
MO000081626     XN000103046
MO000078228     XN000103046

//XN000104428: c-Myc -p300->
MO000082521     XN000104428
MO000056523     XN000104428

//XN000104831: Smad3 -> c-Myc
MO000082956     XN000104831
XN000104831     MO000082521

//XN000105517: TEF-1 -(PKAr)2:(PKAc)2->
MO000082676     XN000105517
MO000058482     XN000105517

//XN000106649: MyoD -p300->
MO000084139     XN000106649
MO000056523     XN000106649

//XN000108063: JunD -JNK1->
MO000060057     XN000108063
MO000057427     XN000108063
```

```
//XN000108197: Cot -AKT-1->
MO000103015     XN000108197
MO000057082     XN000108197

//XN000108821: N-Myc -GCN5->
MO000082621     XN000108821
MO000086602     XN000108821

//XN000109699: c-Jun -AKT-1->
MO000078288     XN000109699
MO000057082     XN000109699

//XN000110137: C/EBPbeta-LIP + C/EBPbeta-isoform2 + C/EBPbeta-isoform3 -Cdk2-alpha->
MO000026350     XN000110137
MO000327631     XN000110137
MO000327632     XN000110137
MO000023199     XN000110137

//XN000112797: p300 -ROCK-II->
MO000056523     XN000112797
MO000090320     XN000112797

//XN000118722: p300 -AKT-1->
MO000056523     XN000118722
MO000057082     XN000118722

//XN000124396: Abl-isoform1 -p300->
MO000060201     XN000124396
MO000056523     XN000124396

//XN000127233: C/EBPbeta-LIP + C/EBPbeta-isoform2 + C/EBPbeta-isoform3 -p300->
MO000026350     XN000127233
MO000327631     XN000127233
MO000327632     XN000127233
MO000056523     XN000127233

//XN000127261: C/EBPbeta-FL -p300->
MO000002639     XN000127261
MO000056523     XN000127261

//XN000128962: PKC-delta-I -Abl-isoform1-> PKCdelta
MO000057524     XN000128962
XN000128962     MO000057589
MO000060201     XN000128962

//XN000128964: p38alpha-isoform1 -PKCdelta->
MO000059917     XN000128964
MO000057589     XN000128964

//XN000130304: PARP-long -p300->
MO000088856     XN000130304
MO000056523     XN000130304

//XN000131022: CBP -IKK-alpha-isoform1->
MO000059021     XN000131022
MO000090848     XN000131022

//XN000138502: PPARgamma2 -ERK1->
MO000033685     XN000138502
MO000056725     XN000138502

//XN000140183: PPARalpha -p38alpha-isoform1->
MO000057686     XN000140183
MO000059917     XN000140183

//XN000147704: VDR -PKCbeta->
MO000088509     XN000147704
MO000086659     XN000147704

//XN000150272: GSK3beta -AKT-1->
MO000057073     XN000150272
MO000057082     XN000150272
```

```
//XN000150751: Smad7-A + Smad7-B -p300->
MO000058700     XN000150751
MO000058714     XN000150751
MO000056523     XN000150751

//XN000151118: proCaspase-3 -PKC-delta-I->
MO000059519     XN000151118
MO000057524     XN000151118

//XN000151149: E12 + E47 -MAPKAPK2->
MO000025923     XN000151149
MO000025925     XN000151149
MO000123092     XN000151149

//XN000151151: AML2beta + AML2alpha -p300->
MO000085924     XN000151151
MO000085926     XN000151151
MO000056523     XN000151151

//XN000151153: Smad2 + Smad2-Short -p300->
MO000083052     XN000151153
MO000257487     XN000151153
MO000056523     XN000151153

//XN000151155: Smad3 -p300->
MO000082956     XN000151155
MO000056523     XN000151155

//XN000151159: Tal-1 -AKT-1->
MO000025936     XN000151159
MO000057082     XN000151159

//XN000151202: c-Jun -p300->
MO000078288     XN000151202
MO000056523     XN000151202

//XN000151238: PP2ACalpha -PKC-delta-I->
MO000085313     XN000151238
MO000057524     XN000151238

//XN000153858: Nrf2 -KEAP1->
MO000058424     XN000153858
MO000112213     XN000153858

//XN000159087: PIAS1 -IKK-alpha-isoform1->
MO000078606     XN000159087
MO000090848     XN000159087

//XN000161636: C/EBPalpha + C/EBPalpha(p30) + C/EBPalpha(p20) -p38alpha-isoform1->
MO000002634     XN000161636
MO000026105     XN000161636
MO000026349     XN000161636
MO000059917     XN000161636

//XN000167451: c-Fos -> FosB + deltaFosB
MO000060047     XN000167451
XN000167451     MO000024840
XN000167451     MO000026218

//XN000181967: Smad3 -> Smad6
MO000082956     XN000181967
XN000181967     MO000020401

//XN000197776: VDR -> RXR-alpha
MO000088509     XN000197776
XN000197776     MO000056800

//XN000197780: VDR -> RXR-beta2 + RXR-beta1
MO000088509     XN000197780
XN000197780     MO000025622
XN000197780     MO000026751
```

```
//XN000197887: p300 -> SRC-1-isoform2
MO000056523     XN000197887
XN000197887     MO000084278

//XN000197888: SRC-1-isoform2 -> VDR
MO000084278     XN000197888
XN000197888     MO000088509

//XN000219717: AML3 + PEBP2alphaA2 + AML3-isoform2 + AML3-G1 + AML3-U1 + AML3-Y1 + AML3-isoform1 +
AML3-G2 + AML3-Y2 -p300->
MO000025371     XN000219717
MO000025372     XN000219717
MO000026272     XN000219717
MO000026315     XN000219717
MO000026316     XN000219717
MO000026317     XN000219717
MO000089847     XN000219717
MO000089850     XN000219717
MO000089851     XN000219717
MO000056523     XN000219717

//XN000225361:  -RSK1-> PIAS3-isoform1
XN000225361     MO000087374
MO000059507     XN000225361

//XN000227655: p300 -> MITF-M + MITF-A + MITF-A1 + MITF-H + MITF-H1 + MITF-H2 + MITF-H3 + MITF-M1 +
MITF-A2
MO000056523     XN000227655
XN000227655     MO000086132
XN000227655     MO000086136
XN000227655     MO000086138
XN000227655     MO000086140
XN000227655     MO000086143
XN000227655     MO000086144
XN000227655     MO000086145
XN000227655     MO000086149
XN000227655     MO000172372

//XN000238873: Skp2-xbb1 -AKT-1->
MO000059253     XN000238873
MO000057082     XN000238873

//XN000252227: ERK1 -> ERK1
MO000166469     XN000252227
XN000252227     MO000056725

//XN000266390: N-Myc -E1->
MO000082621     XN000266390
MO000081629     XN000266390

//XN000267063: AKT-1 -PDK1->
MO000057082     XN000267063
MO000059185     XN000267063

//XN000267302: ERK2 -Cot->
MO000056722     XN000267302
MO000103015     XN000267302

//XN000270598: Nrf2 -GSK3beta->
MO000058424     XN000270598
MO000057073     XN000270598

//XN000275544: C/EBPbeta-FL -RSK2->
MO000002639     XN000275544
MO000084411     XN000275544

//XN000289438: p38alpha -p/CAF->
MO000022214     XN000289438
MO000081626     XN000289438

//XN000369441: sumo1 -PIAS3-isoform1->
```

```
MO000080909     XN000369441
MO000087374     XN000369441

//XN000371881: FosB + deltaFosB -ERK1->
MO000024840     XN000371881
MO000026218     XN000371881
MO000056725     XN000371881

//XN000372283: Abl-isoform1 -> p38alpha
MO000060201     XN000372283
XN000372283     MO000022214

//XN000372356: nanog-isoform2 + nanog-isoform1 -FAK1-isoform3->
MO000079786     XN000372356
MO000079788     XN000372356
MO000058753     XN000372356

//XN000375762: p53as -Melk->
MO000026166     XN000375762
MO000236765     XN000375762

//XN000375803: PDK1 -Melk->
MO000059185     XN000375803
MO000236765     XN000375803

//XN000375949: Smad2 + Smad2-Short -Melk->
MO000083052     XN000375949
MO000257487     XN000375949
MO000236765     XN000375949

//XN000375954: Smad3 -Melk->
MO000082956     XN000375954
MO000236765     XN000375954

//XN000375956: Smad4 -Melk->
MO000088883     XN000375956
MO000236765     XN000375956

//XN000375958: Smad7-A + Smad7-B -Melk->
MO000058700     XN000375958
MO000058714     XN000375958
MO000236765     XN000375958

//XN000378844: Oct3-xbb1 -PKACA-isoform1->
MO000093729     XN000378844
MO000102335     XN000378844

//XN000383850: TGFbetaR-I-isoform1 -Smad7-A->
MO000021275     XN000383850
MO000058700     XN000383850

//XN000384888: BMPR-IA -Abl-isoform1->
MO000134939     XN000384888
MO000060201     XN000384888

//XN000385024: AKT-1 -p300->
MO000057082     XN000385024
MO000056523     XN000385024

//XN000392284: Smad7-A -> myogenin
MO000058700     XN000392284
XN000392284     MO000080384

//XN000393961:  -ERK1-> KEAP1
XN000393961     MO000112213
MO000166469     XN000393961

//XN000401173: c-Jun -ERK1->
MO000078288     XN000401173
MO000166469     XN000401173

//XN000401182: c-Fos -ERK2->
```

```
MO000060047     XN000401182
MO000166473     XN000401182

//XN000401434: AKT-2 -PDK1->
MO000057079     XN000401434
MO000059185     XN000401434

//XN000401941: Smad5 -TGFbetaR-I-isoform1->
MO000081400     XN000401941
MO000021275     XN000401941

//XN000441962: Smad3 -> ERK1
MO000082956     XN000441962
XN000441962     MO000166469

//XN000441963: Smad3 -> ERK2
MO000082956     XN000441963
XN000441963     MO000166473

//XN000441967: Smad3 -> RhoA
MO000082956     XN000441967
XN000441967     MO000087899

//XN000523327: DEC2 -PIAS3-isoform1->
MO000117588     XN000523327
MO000087374     XN000523327

//XN000532108: FAK1-isoform3 -AKT-1->
MO000058753     XN000532108
MO000057082     XN000532108

//XN000535864: c-Myc -Skp2-xbb1->
MO000082521     XN000535864
MO000059253     XN000535864

//XN000538734: POU3F2 -PKACA-isoform1->
MO000095109     XN000538734
MO000102335     XN000538734

//XN000540614: tfeb -PKCbeta->
MO000130592     XN000540614
MO000086659     XN000540614

//XN000551680:  -PP2ACalpha-> CAR2 + CAR
XN000551680     MO000026729
XN000551680     MO000056528
MO000085313     XN000551680

//XN000566680: DEC1 -PIAS1->
MO000180063     XN000566680
MO000078606     XN000566680

//XN000566681: DEC1 -PIAS3-isoform1->
MO000180063     XN000566681
MO000087374     XN000566681

//XN000567229: Oct3 -AKT-1->
MO000042436     XN000567229
MO000057082     XN000567229
```
