# Supplementary material for: Computational Identification of Key Regulators in Two Different Colorectal Cancer Cell Lines
Source: Front Genet. 2016 Apr 5;7:42. doi: 10.3389/fgene.2016.00042 (PMC4820448; doi:10.3389/fgene.2016.00042)
Supplement: Supplementary Table S8 — Master regulatory network based on the 1638NT-1-specific TF set in Pair Graph File format. [file Table8.PDF]

Table S8A. This Pair Graph File contains the pathway reactions including the TRANSPATH molecule IDs for the master regulatory network based on the 1638NT-1-specific TF set (Figure 3). Figure S8B depicts the corresponding network as a Scalable Vector Graphics (SVG) image. Please see Figure 3 for the image description.

```
//XN000000777: MLK3 -> MKK3-isoform1
MO000150298     XN000000777
XN000000777     MO000131967

//XN000000778: MLK3 -> MKK6
MO000150298     XN000000778
XN000000778     MO000102241

//XN000005432:  -MLK3-> MKK4
XN000005432     MO000022206
MO000150298     XN000005432

//XN000005438: p38alpha-isoform1 -MKK4->
MO000059917     XN000005438
MO000022206     XN000005438

//XN000005439: IKK-alpha-isoform1 -MEKK1->
MO000090848     XN000005439
MO000075867     XN000005439

//XN000005443: JNK1 -MKK4-> JNK1
MO000057427     XN000005443
XN000005443     MO000036658
MO000022206     XN000005443

//XN000006712: mdm2-isoform1 -Ubc5A->
MO000078228     XN000006712
MO000103526     XN000006712

//XN000015238: HMGI-C -Cdk1->
MO000153986     XN000015238
MO000087611     XN000015238

//XN000020019: Hsp70-3 -> JNK1
MO000036619     XN000020019
XN000020019     MO000057427

//XN000020144: MKK3-isoform1 -> Dyrk1B-isoform1
MO000131967     XN000020144
XN000020144     MO000121359

//XN000020147: Dyrk1B-isoform1 -> HNF-1alpha
MO000121359     XN000020147
XN000020147     MO000082731

//XN000024757: IRF-3 -TBK1->
MO000120165     XN000024757
MO000149650     XN000024757

//XN000024762: IRF-3 -IKK-beta->
MO000120165     XN000024762
MO000102991     XN000024762

//XN000025127: HSF1short + HSF1long -JNK1->
MO000006879     XN000025127
MO000041093     XN000025127
MO000036658     XN000025127

//XN000025314: c-Jun -Ubc5C->
MO000078288     XN000025314
MO000107910     XN000025314

//XN000028270:  -Siah-2-> Ubc5A + Ubc5C + Ubc5B
XN000028270     MO000103526
XN000028270     MO000107910
XN000028270     MO000107924
MO000021587     XN000028270
```

```
//XN000032499: alpha-synuclein-isoform1 -> 26S proteasome
MO000103350     XN000032499
XN000032499     MO000020202

//XN000039596: beta-catenin -ErbB1->
MO000057343     XN000039596
MO000082356     XN000039596

//XN000039601: beta-catenin -IKK-alpha-isoform1->
MO000057343     XN000039601
MO000090848     XN000039601

//XN000039602: beta-catenin -IKK-beta->
MO000057343     XN000039602
MO000102991     XN000039602

//XN000040484: c-Jun -> TBP
MO000078288     XN000040484
XN000040484     MO000081778

//XN000042655: E1 -Cdk1->
MO000081629     XN000042655
MO000087611     XN000042655

//XN000043432:  -RIP2-> ERK2
XN000043432     MO000023651
MO000103158     XN000043432

//XN000045323: HMGIY-isoform1 + HMGIY-isoform2 -CBP->
MO000111213     XN000045323
MO000153002     XN000045323
MO000059021     XN000045323

//XN000047072:  -MKP-5-> JNK1
XN000047072     MO000057427
MO000126135     XN000047072

//XN000049393: p38alpha-isoform1 -> ipf1
MO000059917     XN000049393
XN000049393     MO000083163

//XN000052403: Sox9 -> Sox5 + L-Sox5 + Sox5-isoform3
MO000088868     XN000052403
XN000052403     MO000108687
XN000052403     MO000171557
XN000052403     MO000257527

//XN000052404: Sox9 -> SOX6-Isoform2 + Sox6-Isoform1
MO000088868     XN000052404
XN000052404     MO000025973
XN000052404     MO000026865

//XN000053104: STAT6 -> NF-AT1 + NF-AT1-isoformC + NF-AT1-isoformB + NF-AT1-isoformD
MO000079083     XN000053104
XN000053104     MO000026042
XN000053104     MO000151281
XN000053104     MO000151283
XN000053104     MO000151284

//XN000057991: p/CAF -> YY1
MO000081626     XN000057991
XN000057991     MO000105093

//XN000061913: HNF-6 -CBP->
MO000057514     XN000061913
MO000059021     XN000061913

//XN000081181: HMGIY-isoform1 + HMGIY-isoform2 -Cdk1->
MO000111213     XN000081181
MO000153002     XN000081181
MO000087611     XN000081181
```

```
//XN000083592: NF-AT1 + NF-AT1-isoformC + NF-AT1-isoformB + NF-AT1-isoformD -p38alpha-isoform1->
MO000026042     XN000083592
MO000151281     XN000083592
MO000151283     XN000083592
MO000151284     XN000083592
MO000059917     XN000083592

//XN000085683: FOXO3a -IKK-alpha-isoform1->
MO000110284     XN000085683
MO000090848     XN000085683

//XN000085833: IKK-alpha-isoform1 -MLK3->
MO000090848     XN000085833
MO000150298     XN000085833

//XN000086070: IKK-beta -TBK1->
MO000102991     XN000086070
MO000149650     XN000086070

//XN000086721: HMGI-C -Nek2->
MO000153986     XN000086721
MO000117415     XN000086721

//XN000087367: FOXO3a -IKK-beta->
MO000110284     XN000087367
MO000102991     XN000087367

//XN000088089: p/CAF -> IRF-2
MO000081626     XN000088089
XN000088089     MO000087463

//XN000088937: YY1 -PARP-long->
MO000105093     XN000088937
MO000088856     XN000088937

//XN000089422: IKK-beta -MLK3->
MO000102991     XN000089422
MO000150298     XN000089422

//XN000091477: TBP -DNA-PKcs->
MO000081778     XN000091477
MO000084852     XN000091477

//XN000091815: DNA-PKcs -PARP-long->
MO000084852     XN000091815
MO000088856     XN000091815

//XN000096393: HSF1short + HSF1long -JNK1->
MO000006879     XN000096393
MO000041093     XN000096393
MO000057427     XN000096393

//XN000098795: NF-AT3-isoform1 + NF-AT3-isoform2 -p38alpha-isoform1->
MO000105046     XN000098795
MO000256370     XN000098795
MO000059917     XN000098795

//XN000098796: NF-AT3-isoform1 + NF-AT3-isoform2 -JNK1->
MO000105046     XN000098796
MO000256370     XN000098796
MO000057427     XN000098796

//XN000103201: p/CAF -Ubc5B->
MO000081626     XN000103201
MO000107924     XN000103201

//XN000118722: p300 -AKT-1->
MO000056523     XN000118722
MO000057082     XN000118722

//XN000119435: Sox9 -PIAS1->
```

```
MO000088868     XN000119435
MO000078606     XN000119435

//XN000130320: PARP-long -CBP->
MO000088856     XN000130320
MO000059021     XN000130320

//XN000131022: CBP -IKK-alpha-isoform1->
MO000059021     XN000131022
MO000090848     XN000131022

//XN000132169: ErbB1 -Ubc5C->
MO000082356     XN000132169
MO000107910     XN000132169

//XN000148436: A20 -IKK-beta->
MO000121772     XN000148436
MO000102991     XN000148436

//XN000150868: Cdk1 -pkmyt1->
MO000087611     XN000150868
MO000146523     XN000150868

//XN000159087: PIAS1 -IKK-alpha-isoform1->
MO000078606     XN000159087
MO000090848     XN000159087

//XN000160177:  -A20-> RIP2
XN000160177     MO000103158
MO000121772     XN000160177

//XN000164305: 26S proteasome -> Nek2
MO000020202     XN000164305
XN000164305     MO000117415

//XN000164340: Ubc5C -> brca1
MO000107910     XN000164340
XN000164340     MO000085226

//XN000178026: p300 -ERK2->
MO000056523     XN000178026
MO000023651     XN000178026

//XN000185837: IRF-7 -TBK1->
MO000088546     XN000185837
MO000149650     XN000185837

//XN000188030: IRF-8 -E1->
MO000088390     XN000188030
MO000081629     XN000188030

//XN000188903: ipf1 -26S proteasome->
MO000083163     XN000188903
MO000020202     XN000188903

//XN000195710: MST1 -> MST1
MO000118776     XN000195710
XN000195710     MO000333178

//XN000232008: IRF-7 -IKK-alpha-isoform1->
MO000088546     XN000232008
MO000090848     XN000232008

//XN000237283: IFNgamma -> IRF-1
MO000081647     XN000237283
XN000237283     MO000087377

//XN000237294: IFNgamma -> IRF-8
MO000081647     XN000237294
XN000237294     MO000088390

//XN000237648: IKK-beta -> JNK3 + JNK1
```

```
MO000102991     XN000237648
XN000237648     MO000030894
XN000237648     MO000036658

//XN000237650: IKK-beta -> MKP-5
MO000102991     XN000237650
XN000237650     MO000126135

//XN000237672: IKK-beta -> PARP-long
MO000102991     XN000237672
XN000237672     MO000088856

//XN000238110: IRF-5 -TBK1->
MO000088834     XN000238110
MO000149650     XN000238110

//XN000240694:  -A20-> traf6-isoform1
XN000240694     MO000086151
MO000121772     XN000240694

//XN000243820: IRF-5 -traf6-isoform1->
MO000088834     XN000243820
MO000086151     XN000243820

//XN000246583: MST1 -JNK1->
MO000118776     XN000246583
MO000036658     XN000246583

//XN000246658: MEKK1 -Ubc5A->
MO000075867     XN000246658
MO000103526     XN000246658

//XN000261216: IRF-1 -IKK-alpha-isoform1->
MO000087377     XN000261216
MO000090848     XN000261216

//XN000371141: alpha-synuclein-isoform1 -Siah-2->
MO000103350     XN000371141
MO000021587     XN000371141

//XN000373192: MKK6 -> ERK2
MO000102241     XN000373192
XN000373192     MO000023651

//XN000374805: hltf -E1->
MO000132131     XN000374805
MO000081629     XN000374805

//XN000379566: Chfr-isoform1 -26S proteasome->
MO000083017     XN000379566
MO000020202     XN000379566

//XN000384117: pkmyt1 -JNK3->
MO000146523     XN000384117
MO000030894     XN000384117

//XN000384119: pkmyt1 -JNK1->
MO000146523     XN000384119
MO000057427     XN000384119

//XN000385717: AKT-1 -26S proteasome->
MO000057082     XN000385717
MO000020202     XN000385717

//XN000504878: STAT6 -TBK1->
MO000079083     XN000504878
MO000149650     XN000504878

//XN000523684: Hsp70-3 -Ubc5A->
MO000036619     XN000523684
MO000103526     XN000523684
```

```
//XN000523698: HSF1short + HSF1long -Ubc5A->
MO000006879     XN000523698
MO000041093     XN000523698
MO000103526     XN000523698

//XN000529428: NF-AT1 + NF-AT1-isoformC + NF-AT1-isoformB + NF-AT1-isoformD -mdm2-isoform1->
MO000026042     XN000529428
MO000151281     XN000529428
MO000151283     XN000529428
MO000151284     XN000529428
MO000078228     XN000529428

//XN000530112: FOXO3a -26S proteasome->
MO000110284     XN000530112
MO000020202     XN000530112

//XN000531488: IRF-3 -> IFNgamma
MO000120165     XN000531488
XN000531488     MO000081647

//XN000531584: HNF-3beta -MST1->
MO000092844     XN000531584
MO000333178     XN000531584

//XN000537719: TBK1 -Ubc5B->
MO000149650     XN000537719
MO000107924     XN000537719

//XN000538238: Sox9 -Ubc5C->
MO000088868     XN000538238
MO000107910     XN000538238

//XN000539261: GATA-6-isoform1 + GATA-6-isoform2 -26S proteasome->
MO000097976     XN000539261
MO000255379     XN000539261
MO000020202     XN000539261

//XN000559520: brca1 -> hoxa9 + hoxa9T
MO000085226     XN000559520
XN000559520     MO000025886
XN000559520     MO000028035

//XN000559521: brca1 -> FOXJ1
MO000085226     XN000559521
XN000559521     MO000117650

//XN000566977: YY1 -> FXR-isoform2 + FXR-isoform1
MO000105093     XN000566977
XN000566977     MO000086345
XN000566977     MO000091320

//XN000570617: FOXP3 -p300->
MO000137560     XN000570617
MO000056523     XN000570617
```
