# Supplementary material for: Computational Identification of Key Regulators in Two Different Colorectal Cancer Cell Lines
Source: Front Genet. 2016 Apr 5;7:42. doi: 10.3389/fgene.2016.00042 (PMC4820448; doi:10.3389/fgene.2016.00042)
Supplement: Supplementary Table S9 — Master regulatory network based on the CMT-93-specific TF set in Pair Graph File format. [file Table9.PDF]

Table S9A. This Pair Graph File contains the pathway reactions including the TRANSPATH molecule IDs for the master regulatory network based on the on the CMT-93-specific TF set (Figure 4). Figure S9B depicts the corresponding network as a Scalable Vector Graphics (SVG) image. Please see Figure 4 for the image description.

```
//XN000000224: ERK2 + ERK1 -> SAP-1a
MO000056722     XN000000224
MO000056725     XN000000224
XN000000224     MO000117427

//XN000000532: gamma-c -> Jak3-isoform1
MO000082567     XN000000532
XN000000532     MO000102629

//XN000000553: Jak3-isoform1 -> STAT3-isoform1 + STAT3-isoform2 + STAT3-isoform3
MO000102629     XN000000553
XN000000553     MO000033431
XN000000553     MO000033432
XN000000553     MO000079462

//XN000000554: STAT5A -> STAT5B
MO000084528     XN000000554
XN000000554     MO000079450

//XN000006966: p53 -ERK2->
MO000056633     XN000006966
MO000023651     XN000006966

//XN000011589: p53 -ERK1->
MO000056633     XN000011589
MO000043191     XN000011589

//XN000015259: STAT3-isoform1 + STAT3-isoform2 + STAT3-isoform3 -ERK1->
MO000033431     XN000015259
MO000033432     XN000015259
MO000079462     XN000015259
MO000056725     XN000015259

//XN000015419: STAT6 -Jak2 -Jak3-isoform1->
MO000079083     XN000015419
MO000059450     XN000015419
MO000102629     XN000015419

//XN000015552: ACLP-isoform1/2 -> ERK1
MO000033710     XN000015552
XN000015552     MO000056725

//XN000017699: c-Ets-2 -ERK1->
MO000025645     XN000017699
MO000056725     XN000017699

//XN000017701: c-Ets-2 -ERK2->
MO000025645     XN000017701
MO000056722     XN000017701

//XN000017706: c-Ets-1 -ERK1->
MO000057516     XN000017706
MO000056725     XN000017706

//XN000020033: MSK1-isoform2 -ERK2->
MO000057429     XN000020033
MO000056722     XN000020033

//XN000021765: Pax-2b -JNK1->
MO000025956     XN000021765
MO000057427     XN000021765

//XN000025344: huntingtin -> REST-isoform1 + REST-isoform2 + REST-isoform3
MO000104304     XN000025344
XN000025344     MO000086592
XN000025344     MO000086770
XN000025344     MO000257093
```

```
//XN000025475: huntingtin -calpain-2->
MO000104304     XN000025475
MO000117099     XN000025475

//XN000026672: calpain-2 -ERK1->
MO000117099     XN000026672
MO000043191     XN000026672

//XN000026673: ERK1 -> calpain-2
MO000056725     XN000026673
XN000026673     MO000117099

//XN000040671: C/EBPalpha -> Elf-1
MO000002634     XN000040671
XN000040671     MO000056620

//XN000042917: Elk-1 -> Elf-1
MO000087492     XN000042917
XN000042917     MO000056620

//XN000043397:  -ERK1-> ERK1
XN000043397     MO000043191
MO000056725     XN000043397

//XN000043398: ERK1 -Jak3-isoform1-> ERK1
MO000056725     XN000043398
XN000043398     MO000043191
MO000102629     XN000043398

//XN000043430: ERK2 -Jak3-isoform1-> ERK2
MO000056722     XN000043430
XN000043430     MO000023651
MO000102629     XN000043430

//XN000046975:  -SHP2-isoform1-> Jak2
XN000046975     MO000059450
MO000056995     XN000046975

//XN000049637: Pax-2a + pax-2short -JNK1->
MO000025100     XN000049637
MO000256654     XN000049637
MO000057427     XN000049637

//XN000050921: Raf-1-isoform1 -ERK1->
MO000058621     XN000050921
MO000056725     XN000050921

//XN000050946: Raf-1-isoform1 -> Fra-2-isoform1 + Fra-2-isoform2
MO000058621     XN000050946
XN000050946     MO000060051
XN000050946     MO000255323

//XN000051944: SHP2-isoform1 -ERK1->
MO000056995     XN000051944
MO000056725     XN000051944

//XN000053922: TNF-alpha -> AP-2alpha-isoform2 + AP-2alpha-isoform3 + AP-2alpha-isoform4 + AP-2alpha-
isoform1
MO000086680     XN000053922
XN000053922     MO000026469
XN000053922     MO000026470
XN000053922     MO000026471
XN000053922     MO000046020

//XN000061219: ERK2 + ERK1 -> ERK2
MO000056722     XN000061219
MO000056725     XN000061219
XN000061219     MO000023651

//XN000061930: c-Ets-1 -ERK2->
MO000057516     XN000061930
```

```
MO000056722     XN000061930

//XN000062408: Elk-1 -ERK1->
MO000087492     XN000062408
MO000056725     XN000062408

//XN000062862: C/EBPalpha -ERK1->
MO000002634     XN000062862
MO000056725     XN000062862

//XN000080479: Elk-1 -ERK2->
MO000087492     XN000080479
MO000077694     XN000080479

//XN000084276: SREBP-2-isoform1 + SREBP-2-isoform2 -ERK1->
MO000130186     XN000084276
MO000257563     XN000084276
MO000056725     XN000084276

//XN000084277: SREBP-2-isoform1 + SREBP-2-isoform2 -ERK2->
MO000130186     XN000084277
MO000257563     XN000084277
MO000056722     XN000084277

//XN000087009: STAT3-isoform1 + STAT3-isoform2 + STAT3-isoform3 -ERK2->
MO000033431     XN000087009
MO000033432     XN000087009
MO000079462     XN000087009
MO000056722     XN000087009

//XN000097457: ERK2 -> ERK2
MO000077694     XN000097457
XN000097457     MO000056722

//XN000100218: Elk-1 -ERK2->
MO000087492     XN000100218
MO000023651     XN000100218

//XN000102498: RelA-p65 + p65 delta -GSK3beta->
MO000083492     XN000102498
MO000090852     XN000102498
MO000057073     XN000102498

//XN000105362: RelA-p65 + p65 delta -p300->
MO000083492     XN000105362
MO000090852     XN000105362
MO000056523     XN000105362

//XN000105487: RelA-p65 + p65 delta -MSK1-isoform2->
MO000083492     XN000105487
MO000090852     XN000105487
MO000057429     XN000105487

//XN000106562: STAT5A -Jak3-isoform1->
MO000084528     XN000106562
MO000102629     XN000106562

//XN000150905: STAT5B -Jak3-isoform1->
MO000079450     XN000150905
MO000102629     XN000150905

//XN000150949: MKP-1 -ERK1->
MO000161717     XN000150949
MO000056725     XN000150949

//XN000151060: p53 -ERK2->
MO000056633     XN000151060
MO000056722     XN000151060

//XN000151259: SREBP-1 + SREBP-1C + SREBP-1A + SREBP-1A-W42 + SREBP-1C-W42 -p300->
MO000028399     XN000151259
MO000081059     XN000151259
```

```
MO000140446     XN000151259
MO000257561     XN000151259
MO000257562     XN000151259
MO000056523     XN000151259

//XN000160912: RelA-p65 -> RelA-p35
MO000083492     XN000160912
XN000160912     MO000129435

//XN000163626: NF-kappaB1-isoform6 + NF-kappaB1-isoform7 + NF-kappaB1-isoform5 + NF-kappaB1-isoform1 +
NF-kappaB1-isoform2 + NF-kappaB1-isoform3 + NF-kappaB1-isoform4 -GSK3beta->
MO000016622     XN000163626
MO000016623     XN000163626
MO000019317     XN000163626
MO000019360     XN000163626
MO000118260     XN000163626
MO000118263     XN000163626
MO000118267     XN000163626
MO000057073     XN000163626

//XN000173487: STAT5A -ERK2 -ERK1->
MO000084528     XN000173487
MO000056722     XN000173487
MO000056725     XN000173487

//XN000178026: p300 -ERK2->
MO000056523     XN000178026
MO000023651     XN000178026

//XN000220983: SHP2-isoform1 -fgfr1-isoform1->
MO000056995     XN000220983
MO000059629     XN000220983

//XN000232963: GSK3beta -ERK2 -ERK1->
MO000057073     XN000232963
MO000056722     XN000232963
MO000056725     XN000232963

//XN000277090: ACLP-isoform1/2 -> TNF-alpha
MO000033710     XN000277090
XN000277090     MO000086680

//XN000287931: Gli2 -ERK2->
MO000168064     XN000287931
MO000056722     XN000287931

//XN000288345: ERF -ERK2->
MO000161875     XN000288345
MO000056722     XN000288345

//XN000289152:  -MKP-1-> JNK1
XN000289152     MO000057427
MO000161717     XN000289152

//XN000374807: ERK2 -Raf-1-isoform1->
MO000056722     XN000374807
MO000058621     XN000374807

//XN000400945: fgfr1-isoform1 -ERK2->
MO000059629     XN000400945
MO000056722     XN000400945
```
